# Bringing biocatalytic deuteration into the toolbox of asymmetric isotopic labelling techniques

**DOI:** 10.1038/s41467-020-15310-z

**Published:** 2020-03-19

**Authors:** J. S. Rowbotham, M. A. Ramirez, O. Lenz, H. A. Reeve, K. A. Vincent

**Affiliations:** 1 Department of Chemistry, University of Oxford, Inorganic Chemistry Laboratory, South Parks Road, Oxford, OX1 3QR UK; 2 Department of Chemistry, Technische Universität Berlin, Strasse des 17. Juni 135, 10623 Berlin, Germany

**Keywords:** Asymmetric catalysis, Biocatalysis, Asymmetric synthesis

## Abstract

Enzymes dependent on nicotinamide cofactors are important components of the expanding range of asymmetric synthetic techniques. New challenges in asymmetric catalysis are arising in the field of deuterium labelling, where compounds bearing deuterium (^2^H) atoms at chiral centres are becoming increasingly desirable targets for pharmaceutical and analytical chemists. However, utilisation of NADH-dependent enzymes for ^2^H-labelling is not straightforward, owing to difficulties in supplying a suitably isotopically-labelled cofactor ([4-^2^H]-NADH). Here we report on a strategy that combines a clean reductant (H_2_) with a cheap source of ^2^H-atoms (^2^H_2_O) to generate and recycle [4-^2^H]-NADH. By coupling [4-^2^H]-NADH-recycling to an array of C=O, C=N, and C=C bond reductases, we demonstrate asymmetric deuteration across a range of organic molecules under ambient conditions with near-perfect chemo-, stereo- and isotopic selectivity. We demonstrate the synthetic utility of the system by applying it in the isolation of the heavy drug (1*S*,3’*R*)-[2’,2’,3’-^2^H_3_]-solifenacin fumarate on a preparative scale.

## Abstract (web summary)

Deuterated chemicals are widely exploited in analytical chemistry and increasingly in the synthesis of pharmaceutical compounds. Here, the authors developed a mild biocatalytic method for the selective asymmetric reductive deuteration of organic compounds, by using H_2_ and 2H_2_O to generate 2H-NADH.

## Introduction

Deuteration has long been used to alter the properties of molecules across a broad range of disciplines, from physical and life sciences, to forensics and environmental monitoring^1–4^. Indeed, the presence of deuterium can have implications for molecular properties far beyond the augmented mass, including the alteration of spin characteristics (particularly valuable in nuclear magnetic resonance (NMR) spectroscopy), vibrational states, optical properties, neutron scattering and even solubility^4^. One of the most significant impacts of deuteration is manifested by the so-called deuterium kinetic isotope effect (DKIE), where cleavage of a C-^2^H bond can be many times slower than for the C-^1^H counterpart^3^. For decades, DKIEs have been exploited to provide insight into the mechanisms of chemical and biological processes^1,4,5^. More recently, there has been interest in exploiting potential therapeutic benefits of the DKIE to alter the pharmacokinetic properties of drug candidates and their associated metabolic profiles^3,6–9^. Here, deuterium atoms serve to stabilise compounds against physiological degradation routes, such as racemisation and oxidation, leading to improved pharmacokinetic profiles, which allow for lower doses, as well as minimising or avoiding the production of potentially toxic metabolites^3,8,10^.

**Fig. 1:**
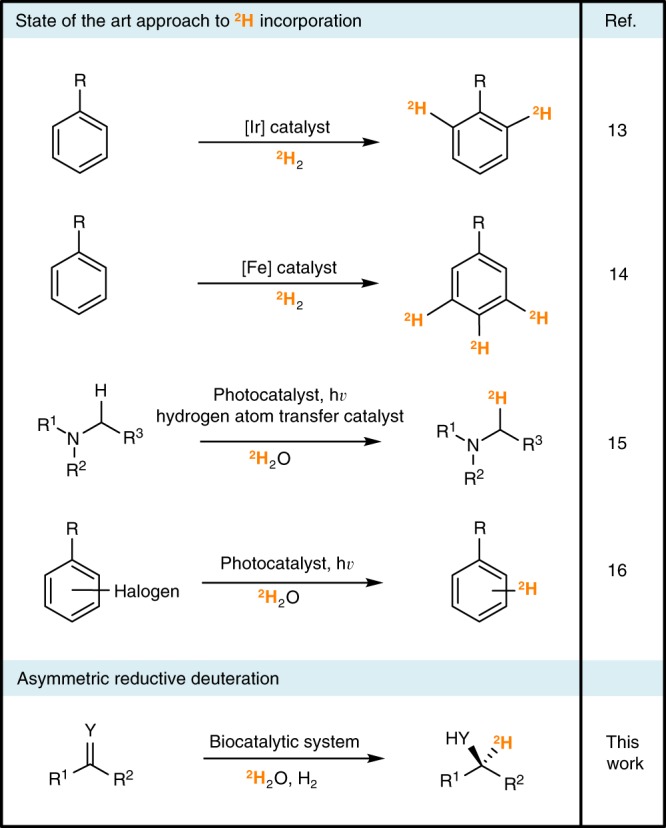
Methods for introduction of deuterium into organic molecules and drug candidates. State-of-the-art approaches for carbon–hydrogen or carbon–halogen hydrogen isotope exchange; these methods use either ^2^H_2_ or ^2^H_2_O as the deuterium source. The biocatalytic reductive deuteration strategy demonstrated in this work uses ^2^H_2_O as the deuterium source and H_2_ as a clean reductant.

The interest in deutero-drugs has increased the demand for catalysts that are capable of precision deuteration^8^, installing deuterium atoms at well-controlled molecular positions. Hydrogen isotope exchange (HIE) and halogen–deuterium exchange catalysts allow for post-synthetic deuteration by treating the unlabelled compound in ^2^H_2_O or under ^2^H_2_ gas^8,11,12^. Developments in these catalysts have greatly improved the regio-selectivity of deuteration, with notable examples highlighted in Fig. 1^13–16^. However, synthetic strategies for the enantioselective installation of deuterium are not well established. While a limited number of catalysts have been demonstrated for asymmetric HIE^17–19^, these post-synthetic methodologies can deteriorate the enantiomeric purity of the compound in question^8^. Similarly, there are limited examples of chemocatalysts for asymmetric reductive deuteration, and challenges remain around their enantio- and isotopic selectivity^20–22^.

**Fig. 2:**
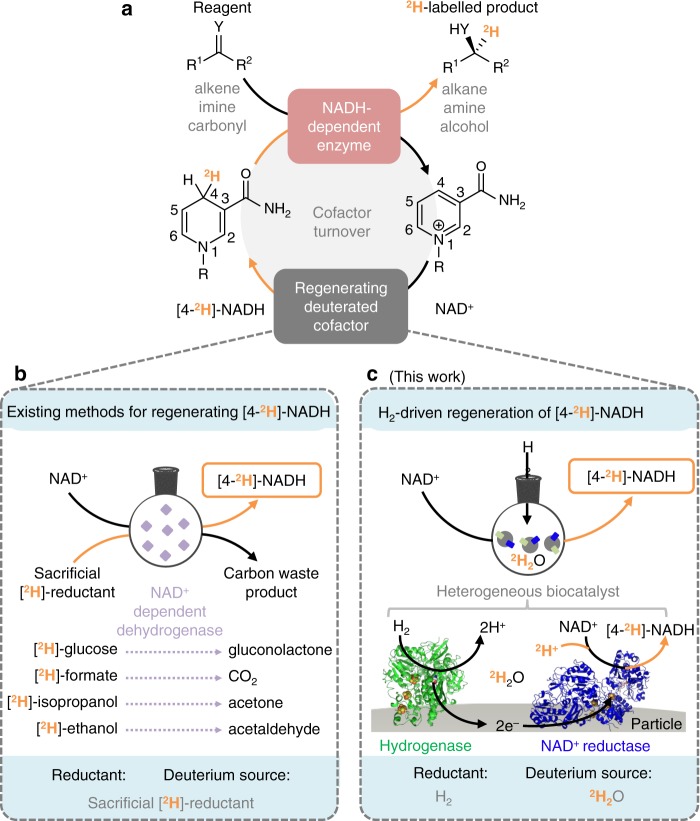
Biocatalytic reductive deuteration via recycling of [4-^2^H]-NADH. **a** Biocatalytic reductive deuteration requires a supply of [4-^2^H]-NADH. **b** Conventional [4-^2^H]-NADH-recycling strategies are driven by a sacrificial deuterated reductant. **c** Schematic representation of the heterogeneous biocatalytic system demonstrated in this work for H_2_-driven generation of the labelled cofactor [4-^2^H]-NADH, with ^2^H_2_O supplying the deuterium atoms. For complete structures of the cofactors, see Supplementary Fig. 1 and for details of the heterogeneous biocatalyst, see Supplementary Methods.

In the field of asymmetric synthesis, biocatalysis has profoundly expanded the toolbox available to organic chemists, with enzymatic transformations offering near-perfect selectivity and mild operating conditions^23^. Advanced enzyme evolution techniques have dramatically expanded the scope of biocatalysis, with Sitagliptin providing a notable case study in biocatalytic pharmaceutical synthesis^24^. However, despite the growing importance of NADH-dependent reductase enzymes in chiral fine chemical synthesis^25^, significant hurdles have impeded their use in deuteration chemistry due to the requirement for deuterated, reduced cofactor, [4-^2^H]-NADH, which must be continually regenerated in situ (Fig. 2a). Demonstrations, at laboratory scale, have relied on a super-stoichiometric supply of a sacrificial deuterated reductant, [^2^H]-ethanol, [^2^H]-isopropanol, [^2^H]-glucose or [^2^H]-formate, for example, in conjunction with the appropriate dehydrogenase enzyme (Fig. 2b)^26–28^. Such compounds are labour intensive and expensive to prepare, adding cost and complexity to the process^27^. Conversely, researchers in the field of chemocatalytic deuteration have cited the desirability to use ^2^H_2_O as both the solvent and source of isotopes, owing to the inherent safety, cost efficiency, availability and easy handling of this material^16,29^. As such, we aimed to develop a method for conducting biocatalytic deuteration reactions whereby ^2^H could be incorporated directly from a heavy water reaction medium.

We have previously demonstrated H_2_-driven NADH recycling using hydrogenase and NAD^+^ reductase (both produced in-house, Supplementary Methods) co-immobilised on a carbon support^30,31^. This heterogeneous biocatalyst system couples H_2_ oxidation by a nickel-iron hydrogenase to the reduction of NAD^+^ to NADH by a flavin-containing NAD^+^ reductase moiety via electron transfer through the carbon^31^. Since the H_2_ oxidation and NAD^+^ reduction catalytic sites are spatially separated, we hypothesised that operation of this system in ^2^H_2_O, instead of H_2_O, would enable the NAD^+^ reductase to transfer deuterium ions from solution to NAD^+^, thereby forming [4-^2^H]-NADH (Fig. 2c) even under H_2_ gas. This suggested the possibility of a route to recycling the deuterated cofactor, suitable for application in the biocatalysed synthesis of selectively deuterated chemicals, in which the heavy atom label is provided solely by the solvent, ^2^H_2_O. Here we report on the application of the H_2_-driven biocatalytic system for forming and recycling [4-^2^H]-NADH in an array of C=O, C=N and C=C bond reductions. The resulting products illustrate the high chemo-, regio-, stereo- and isotopic selectivity that can be achieved through enzymatic deuteration, all under mild reaction conditions, and with ^2^H_2_O as the only source of ^2^H. We then illustrate the synthetic utility of this biocatalytic approach in the preparation of deuterium-labelled asymmetric molecules, including deutero-drugs and drug fragments.

## Results

### H_2_-driven biocatalytic system for generation of [4-^2^H]-NADH

To test our hypothesis that the heterogeneous biocatalyst system would form deuterated NADH, we supplied it with 4 mM NAD^+^ in ^2^H_2_O under an atmosphere of H_2_ gas (see Supplementary Methods for details). Subsequent analysis by ultraviolet (UV)–visible and proton NMR (^1^H NMR) spectroscopy (Supplementary Fig. 4) confirmed that only the biologically relevant [4]-NADH was generated (at 97% conversion), with the anticipated incorporation of ^2^H at the [4]-position on the nicotinamide ring ([4-^2^H]-NADH)^31^.

### Scope of biocatalytic reductive deuteration

**Fig. 3:**
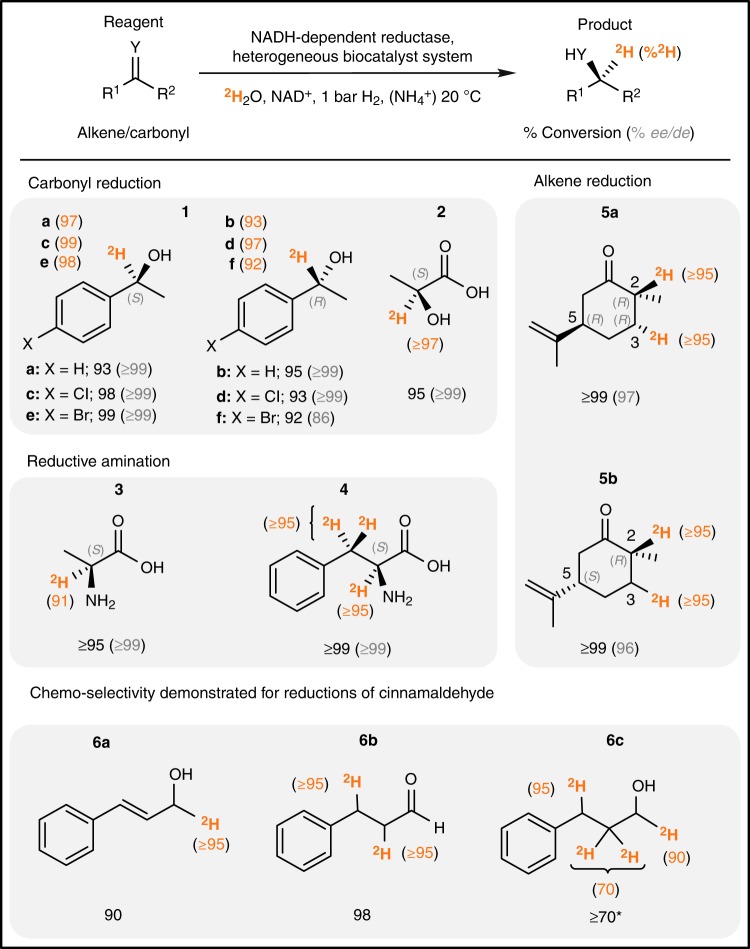
Scope of asymmetric biocatalytic reductive deuteration. Typical reaction conditions: 0.5 mL, 5 mM reagent, 0.5 mM NAD^+^, ^2^H_2_O (98 %), Tris-^2^HCl (p^2^H 8.0, 100 mM), 1–5 vol.% ^2^H_6_-DMSO, 16 h, 20 ^o^C, 1 bar H_2_ and shaking at 500 r.p.m. Catalysts: Excess NADH-dependent reductase, 400 μg carbon (160 pmol hydrogenase, 260 pmol NAD^+^ reductase). For reductive amination reactions: 25 mM NH_4_Cl. *All transformations were fully selective for the reduction of the desired functional group except **6c**, for which a known enzymatic side reaction yielded an extra product (see Supplementary Fig. 47 and associated discussion). Full experimental and analytical details are provided in Supplementary Methods.

Following this successful demonstration, we then sought to use H_2_-dependent biocatalysis to recycle [4-^2^H]-NADH to drive a range of NADH-dependent reductases. The selected reductases were all from commercial enzyme suppliers, and were chosen to cover three of the most prominent classes of reductive biotransformations: carbonyl reduction, carbonyl reductive amination, and alkene reduction. By operating the heterogeneous biocatalyst system with the relevant reductase in ^2^H_2_O, under mild reaction conditions (p^2^H 8.0, 1 bar H_2_, 20 °C), we were able to demonstrate reactions to form a diverse array of molecules bearing deuterium atoms in carefully controlled positions (Fig. 3, full reaction schemes and product characterisation in Supplementary Methods). A steady flow of H_2_ across the reaction headspace was found to be sufficient to drive the reactions, but conventional balloons and sealed pressure vessels were also shown to be suitable alternative setups. In all cases, the chemo-, stereo- and regio-selectivity known for the enzymatic reactions in natural abundance H_2_O was fully retained upon translation to the deuteration conditions.

The reductive deuteration of a suite of reagents, via H_2_-driven recycling of [4-^2^H]-NADH, was achieved by combining the heterogeneous biocatalytic cofactor recycling system with an appropriate reductase, either co-immobilised or in solution. The products in Fig. 3 demonstrate the versatility of this approach. For carbonyl reduction, for example, it was possible to generate either (*S*)-[1-^2^H]- or (*R*)-[1-^2^H]-1-phenylethanol (**1a** and **1b**) from acetophenone, with ≥93% isotopic selectivity and ≥99% enantioselectivity, by choosing an (*S*)- or (*R*)-selective alcohol dehydrogenase (keto reductase), respectively. Similarly, 4-chloroacetophenone and 4-bromoacetophenone could be reduced to give the corresponding chlorinated (**1c** and **1d**) and brominated (**1e** and **1f**) products with similar selectivity, and preserving the aryl halide for potential onward cross-coupling chemistry. Pyruvate could be converted to l-[2-^2^H]-lactate (**2**) by the action of l-lactate dehydrogenase, or to l-[2-^2^H]-alanine (**3**) by the action of l-alanine dehydrogenase in the presence of an amine source (NH_4_^+^). Reductive amination was also carried out on phenylpyruvate using l-phenylalanine dehydrogenase; prior exchange of the β-protons with ^2^H_2_O yields a triply deuterated product: l-[2,2,3-^2^H_3_]-phenylalanine (**4**). Deuterated amino acids have been identified as particularly valuable targets owing to their pharmacological applications (such as [^2^H_3_]-d-serine and [^2^H_3_]-l-DOPA)^32^, and their use in protein NMR spectroscopy^33^.

Asymmetric alkene reduction was demonstrated by the use of an ene-reductase on (5*R*)- and (5*S*)-carvone reagents, yielding *trans-*(2 *R*,5*R*)- and *cis-*(2*R*,5*S*)-dideuterocarvone (**5a** and **5b**), respectively. Here, the enzyme generates (*R*)-stereochemistry at the prochiral 2-position of the two parent carvones, with equally high *de* in each case. ^1^H NMR spectroscopic analysis of product **5a** (see Supplementary Methods) demonstrates that the deuterium atom at the 3-position of the dideuterocarvone is also added asymmetrically, giving (*R*)-stereochemistry at this position, in accordance with the established mechanism of ene reductases in reducing double bonds in an anti-manner^34,35^. The ability to introduce isotopically induced chirality by selective biocatalytic deuteration may have further important implications in drug design and generation of deuterated chemicals for analytical applications^36^.

In a final screening experiment, we demonstrated the potential for chemo-selective deuteration of another molecule containing both alkene and carbonyl functionalities, αβ-unsaturated cinnamaldehyde. Reduction of cinnamaldehyde by means of conventional heterogeneous catalysis can suffer from poor chemoselectivity, giving rise to products from mixed reduction and over-reduction^37^. However, by utilising an ene-reductase and alcohol dehydrogenase in isolation or sequentially, the singly deuterated cinnamyl alcohol (**6a**), doubly deuterated hydrocinnamaldehyde (**6b**) or multiply deuterated 3-phenyl-1-propanol (**6c**) can all be separately prepared.

### Application of catalyst for preparing deuterated drugs

**Fig. 4:**
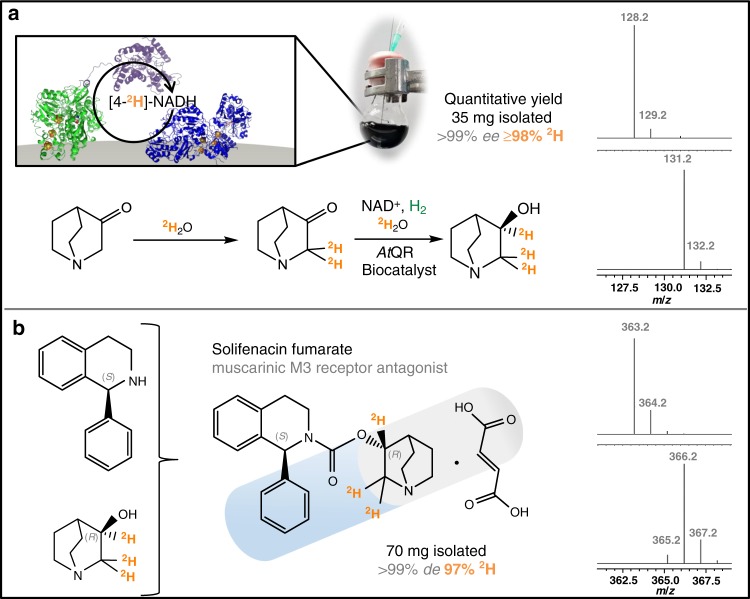
Scalable biocatalytic deuteration for preparation of heavy solifenacin fumarate. **a** Preparation of trideuterated (*R*)-3-quinuclidinol via biocatalytic reductive deuteration and **b** incorporation into the pharmaceutical compound (1*S*,3′*R*)-solifenacin fumarate. In each case, the mass spectroscopy traces show the expected +3 shift in *m*/*z* values of the isolated product (lower plot) relative to their natural abundance standards (upper plot). Full experimental and analytical details are provided in Supplementary Methods.

Following confirmation of the scope of the biocatalytic system for C=O, C=C and C=N bond reductive deuteration, the catalyst was tested for its applicability under standard benchtop synthesis conditions (10 mL reaction solution, round bottom flask, under gentle flow of H_2_). The chiral alcohol (*R*)-3-quinuclidinol was selected as the target compound for these studies, as it is a key component in a number of drug molecules, such as talsaclidine, cevimeline and solifenacin^38,39^. In order to synthesise the chiral alcohol, a quinuclidinone reductase from *Agrobacterium tumefaciens* (*At*QR) was expressed in *Escherichia coli* and purified accordingly (Supplementary Methods)^40,41^. When supplied with NADH and 3-quinuclidinone, the *At*QR is capable of producing (*R*)-3-quinuclidinol with >99% *ee*. As such, the *At*QR was combined with the H_2_-driven deuteration catalyst in order to transfer a ^2^H-atom to the (3*R*)-chiral centre with equally high selectivity (see Fig. 4a). Here, the protons α to the amine and the carbonyl group in the substrate freely exchange with the ^2^H_2_O solvent, resulting in (*R*)-[2,2,3-^2^H_3_]-3-quinuclidinol as the final product. The reaction was conducted on a preparative scale (35 mg), and proceeded with the anticipated >99% *ee* and >98% ^2^H incorporation on the two carbon atoms (Supplementary Methods). The heterogeneous catalyst could simply be filtered off and, following basification and extraction, the product was obtained in a crude form in a near quantitative yield. The only contaminant at this stage was a trace amount of glycerol, which could likely be eliminated by exchanging the storage buffer of the enzymes prior to use. While precautions were taken to exclude O_2_, the use of the robust, O_2_-tolerant hydrogenase 1 from *E. coli* meant that the reaction did not need to be rigorously anaerobic to succeed^42,43^.

Having isolated sufficient quantities of (*R*)-[2,2,3-^2^H_3_]-3-quinuclidinol, the compound was used to generate a deuterated form of the antimuscarinic drug, solifenacin fumarate, on a scale suitable for onward biochemical investigation^44,45^. Conventional synthetic routes to the drug typically rely on crystallographic resolution of a racemate; however, that step was avoided here owing to the enantiomeric purity of the quinuclidinol moiety utilised^44^. Indeed, the simultaneous installation of the deuterium with the creation of the chiral centre is preferable to a post-synthetic deuteration strategy with a HIE catalyst, where the *ee* may be diminished. It is worth noting that the other chiral building block, (*S*)-1-phenyl-1,2,3,4-tetrahydroisoquinoline, which we obtained commercially, has recently been shown to be accessible by different enzymatic strategies, and may also be targeted for asymmetric deuteration in a similar manner^46–48^. Following the synthesis, it was established that the stereo- and isotopic purity of the starting quinuclidinol was carried through to the final solifenacin fumarate product (see Fig. 4b and Supplementary Methods).

In summary, we have demonstrated a versatile approach to asymmetric reductive deuteration that exploits the near-perfect selectivity of NADH-dependent reductases. The system enables the formation of a deuterium-labelled chiral centre in a single step with high chemo-, stereo- and isotopic selectivity. The catalyst operates under mild conditions using a simple reductant (H_2_), coupled with a cheap and abundant source of deuterium (^2^H_2_O), and therefore represents a major advance compared to existing bio- and chemocatalytic deuteration strategies.

Initially, we showed high %^2^H incorporation across diverse functional group space to highlight that the major classes of NADH-dependent biocatalytic transformations (ketone reduction, ene reduction and reductive amination) can be achieved with retention of the original enzyme selectivity. The subsequent library of asymmetric [^2^H]-labelled molecules generated will be valuable to researchers across a diverse range of fields. In particular, asymmetric deuterium centres are increasingly desirable to synthetic medicinal chemists, so we further demonstrated the applicability of our system for the preparative-scale deuteration of the chiral drug fragment (*R*)-3-quinuclidinol. Here it was shown that the biocatalytic deuteration approach was fully compatible with standard benchtop synthetic techniques and hydrogenation protocols, with the advantage of relying on mild temperatures and H_2_ pressures. Furthermore, the use of H_2_ and ^2^H_2_O as reagents, catalytic quantities of cofactor and the use of carbon-supported enzymes mean that only very simple purification strategies are required, simplifying downstream transformations. This point was illustrated by the subsequent synthesis and isolation of a deuterated analogue of the incontinence drug, solifenacin. Notably, this strategy of introducing the isotope label in the same step as the formation of the chiral centre, allowed for the formation of the final product without the necessity of post-synthetic HIE.

Hence, by coupling the system reported here with the increasing range of available NADH-dependent enzymes, we anticipate that biocatalysis will provide a powerful complementary addition to the growing toolbox of deuterium incorporation techniques.

## Supplementary information

Supplementary Information

## Data Availability

All data are available from the authors upon reasonable request.
