# Supplementary material for: Bringing biocatalytic deuteration into the toolbox of asymmetric isotopic labelling techniques
Source: Nat Commun. 2020 Mar 19;11:1454. doi: 10.1038/s41467-020-15310-z (PMC7081218; doi:10.1038/s41467-020-15310-z)
Supplement: Supplementary file 1 — Supplementary Information [file 41467_2020_15310_MOESM1_ESM.pdf]

Supplementary Information

Bringing biocatalytic deuteration into the toolbox of asymmetric isotopic labelling techniques

J. S. Rowbotham *et al.*

Table of contents

Supplementary Figures 3
Supplementary Tables 3
Supplementary Methods 4
Solvents, reagents, and catalysts 4
Conducting and analysing ^{2}H-labelling reactions 8
Conversion and characterisation of NAD$^+$ to [4*S*-^{2}H]-NADH 11
Reductive deuteration of acetophenone (**1a**, **1b**) 13
Reductive deuteration of 4′-chloroacetophenone (**1c**, **1d**) 17
Reductive deuteration of 4′-bromoacetophenone (**1e**, **1f**) 21
Reductive deuteration of pyruvate (**2**) 24
Reductive amination and deuteration of pyruvate (**3**) 28
Reductive amination and deuteration of phenylpyruvate (**4**) 33
Reductive deuteration of carvones (**5a**, **5b**) 38
Reductive deuteration of cinnamaldehyde (**6a**, **6b**, **6c**) 44
Synthesis of (3*R*)-[2,2,3-$^2H_3$]-quinuclidinol 53
Synthesis of (1*S*,3′*R*)-[2′,2′,3′-$^2H_3$]-solifenacin fumarate 57
Supplementary References 62

Supplementary Figures

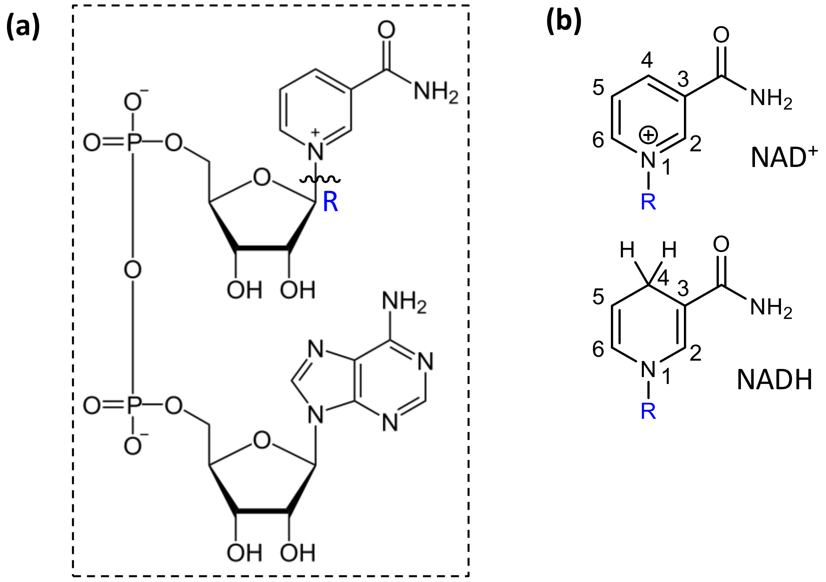

Supplementary Figure 1: Structure of the nicotinamide cofactors. (a) Full structure of cofactor (oxidised only) and **(b)** abbreviated structure of cofactor (oxidised and reduced).

Supplementary Tables

Supplementary Table 1: NMR spectroscopic parameters used in this work

Machine	Avance III - 400	Avance III - 500	Avance III - 500	Avance III - 500
Nucleus	^{1}H	^{1}H	^{2}H	^{13}C
RF pulse energy (MHz)	400.13	499.9	76.7	125.7
Temperature (K)	298 ± 2	298 ± 2	298 ± 2	298 ± 2
Number of scans	*As required*	*As required*	64	1024
Pulse width (μs)	14.0	10.3	407.5	9.3
Spectral width (Hz)	8000	8000	2300	30000
Acquisition time (s)	4.09	2.04	1.78	1.10
Relaxation delay (s)	1.00	2.00	0.3	2.00

Supplementary Methods

Solvents, reagents, and catalysts

General reagents: General reagents and buffer salts (Sigma Aldrich), NAD^+ and NADH (Prozomix), and carbon black particles (Black Pearls 2000, BP2000, Cabot Corporation), were all used as received without further purification. All non-deuterated solutions were prepared with MilliQ water (Millipore, 18 MΩcm), and deuterated solutions with ^2H_2O (99.98 %, Sigma Aldrich). All solvents were deoxygenated by sparging with dry N_2 for 60 minutes prior to use. All other reagents and solvents were purchased from Sigma Aldrich and used as received, unless specified otherwise.

Deuterated buffers: ($^2H_5$)-Tris-^{2}HCl was prepared by dissolving the required amount of Trizma® base in ^2H_2O and then evaporating to dryness. After repeating twice more, the pD (p^{2}H) of the Tris solution was adjusted to 8.0 by the addition of small aliquots of ^{2}HCl (3.0 M) prior to deoxygenation by sparging with dry N_2. Solutions of fully deuterated ($^2H_{11}$)-Tris-^{2}HCl were also used for analytical purposes, and were prepared in a similar manner. ^{1}H NMR spectroscopy indicated that the final %^2H_2O of deuterated buffer solutions was not below 98 mol.%.

Enzymes: Commercial samples of alcohol dehydrogenases (*R*)-ADH (ADH101) and (*S*)-ADH (ADH105), and an ene-reductase (ENE101) were obtained from Johnson Matthey (JM). *L*-Phenylalanine dehydrogenase (from *Sporosarcina sp.*), *L*-alanine dehydrogenase (recombinant from *Escherichia coli*) and *L*-lactate dehydrogenase (from rabbit muscle) were all purchased from Sigma Aldrich. All commercial enzymes were received from the vendors in their lyophilised forms and used without further purification.

The hydrogenase (O_2-tolerant *Escherichia coli* hydrogenase 1, molecular weight 100 kDa) was expressed using *E. coli* strain FTH004. *E. coli* FTH004 is a strain previously engineered[1] to facilitate hydrogenase purification. Its chromosome encodes a 6×Histidine tag fused at the C-terminus of the small structural subunit of the hydrogenase. A pre-inoculum of *E. coli* FTH004 was grown aerobically from a single colony in 10 mL of Luria-Bertani (LB) medium and incubated for 15 hours at 37 ˚C, 220 rpm. This culture was used to inoculate 6 L of LB medium supplemented with 0.5% wv^{-1} glycerol and 0.4% wv^{-1} sodium fumarate inside a glass bottle. The culture was incubated anaerobically at 37 ˚C for 17 hours without shaking. Subsequently, cells were harvested by centrifugation at 6000 × g, 4 ˚C for 20 minutes. Cell pellets were frozen and stored at -80 ˚C. To isolate the enzyme, frozen cell pellets were thawed at room temperature and resuspended in Buffer A (100 mM Tris, 350 mM NaCl, 3% TritonX-100, pH 7.5). The buffer was supplemented with DNase at 30 mg L^{-1}, and lysozyme at 120 mg L^{-1}, as well as Complete EDTA-free protease inhibitor mixture tablets (Roche Molecular Biochemicals) using one table per 20 mL of buffer. Cells were disrupted by sonication (Fisherbrand™ Q500 Sonicator fitted with standard 0.5-inch probe; Amp 30, 2-second pulse, 5-second pause, total sonication of 5 minutes) and centrifuged at 18,000 × g for 1 hour at 4 ˚C. Next, the supernatant was filtered through a 0.45 μm syringe filter unit, and the resulting filtrate was passed through a previously equilibrated 5 mL Ni-NTA HisTrap FF column (GE Healthcare Life Sciences) using an ÄKTA Start protein purification system (GE Healthcare Life

Sciences). The column was washed with Buffer B (20 mM Tris, 350 mM NaCl, 60 mM imidazole, 0.02% Triton X-100, 1 mM DTT, at pH 7.2). The target protein was eluted with an imidazole gradient between 60 mM and 750 mM imidazole in Buffer B. Purified protein was buffer exchanged into Storage Buffer (50 mM Tris, 350 mM NaCl, 1 mM DTT, at pH 7.2) using a 50-kDa ultra-filtration spin column (Amicon Ultra, Millipore, Billerica, MA). Aliquots of purified protein were flash-frozen in liquid nitrogen and stored at −80 ˚C until use.

The NAD^+ reductase (HoxH-I64A variant of the soluble hydrogenase from *Ralstonia eutropha*, in which the inherent hydrogenase moiety is inactivated[2], molecular weight 170 kDa) was expressed using the *Ralstonia eutropha* strain HF918. *R. eutropha* HF918 is the mega-plasmid free *R. eutropha* HF210 harbouring an overexpression plasmid for the production of the HoxH-I64A mutant enzyme fused to a strep-tag sequence at the N-terminus of the HoxF subunit[3]. The strain HF918 was grown from a frozen glycerol stock followed by heterotrophic growth as follows. A pre-inoculum was grown for 2 days in 5 mL of FN medium (mineral salts[4] containing 25 mM Na_2HPO_4, 11 mM KH_2PO_4, at pH 7.0, 0.2% wv^{-1} NH_4Cl, 0.8 mM $MgSO_4$, 77.5 μM $CaCl_2$, 1 μM $NiCl_2$, 40 μM $FeCl_3$, and 1 μM $ZnCl_2$) with 0.4% wv^{-1} fructose as carbon source, supplemented with 10 μg mL^{-1} tetracycline. The pre-inoculum was then added to 1.8 L of FGN medium (mineral salts as described above) with 0.05% wv^{-1} fructose and 0.4% wv^{-1} glycerol as carbon sources supplemented with10 μg mL^{-1} tetracycline inside a 2 L glass bottle capped with aluminium foil. The culture was incubated at 30 ˚C, 150 rpm until optical density at 436 nm reached approximately 10 absorbance units. Incubation took approximately 7 days. After incubation, cells were harvested by centrifugation at 6000 × g, 4 ˚C for 20 minutes and frozen at -80 ˚C. To isolate the enzyme, frozen cell pellets were thawed at room temperature and resuspended in Buffer M (50 mM Tris, 350 mM KCl, pH 7.5). The buffer was supplemented with DNase at 30 mg L^{-1}, and lysozyme at 120 mg L^{-1}, as well as Complete EDTA-free protease inhibitor mixture tablets (Roche Molecular Biochemicals) using one table per 20 mL of buffer. Cells were disrupted by sonication (Fisherbrand™ Q500 Sonicator fitted with standard 0.5-inch probe; Amp 30, 2-second pulse, 5-second pause, total sonication of 10 minutes) and centrifuged at 18,000 × g for 2 hours at 4 ˚C. Subsequently, the supernatant was filtered through a 0.45 μm syringe filter unit, and the resulting filtrate was passed through a previously equilibrated 10-mL Strep-Tactin Superflow gravity column (IBA). The column was washed with 50 mL of Buffer M. The target protein was eluted with Buffer M containing 2.5 mM desthio-biotin (IBA). Purified protein was concentrated using a 50-kDa ultra-filtration spin column (Amicon Ultra, Millipore, Billerica, MA). Aliquots of purified protein were flash-frozen in liquid nitrogen and stored at −80 ˚C until use.

The NADH-dependent 3-quinuclidinone reductase from *Agrobacterium tumefaciens* (*At*QR) was overexpressed in *E. coli*.[5] The plasmid encoding this enzyme was obtained as follows: Protein sequence from NCBI accession No. AB469148 was used to generate a DNA sequence with optimised codons for *E. coli* over-expression using GeneArt Gene Synthesis services (ThermoFisher Scientific UK). The gene was purchased as an insertion within the pET100 vector (harbouring an ampicillin resistant gene) that included an N-terminal 6×His tag, a protease site and a Western-blot epitope sequence at the N-terminus. Below is the translated protein sequence. Underlined letters correspond to the aforementioned fused amino acids.

MRGSHHHHHHGMASMTGGQQMGRDLYDDDDKDHPFTMAGIFDLSGRKAIVTGGSKGIGAAIARAL
DKAGATVAIADLDVMAAQAVVAGLENGGFAVEVDVTKRASVDAAMQKAIDALGGFDLLCANAGVST
MRPAVDITDEEWDFNFDVNARGVFLANQIACRHFLASNTKGVIVNTASLAAKVGAPLLAHYSASKFAVF
GWTQALAREMAPKNIRVNCVCPGFVKTAMQEREIIWEAELRGMTPEAVRAEYVSLTPLGRIEEPEDVA
DVVVFLASDAARFMTGQGINVTGGVRMD

The resulting plasmid, pET100_*At*QR, was used as received from the vendor without further DNA modifications or cloning. The plasmid pET100_*At*QR was used to transform *E.coli* BL21(DE3) cells (Agilent Technologies LDA UK Limited). Transformed cells were plated on a LB-agar solid medium containing 100 μg mL^{-1} ampicillin. The following day, a single colony was inoculated into 10 mL of LB medium containing 100 μg mL^{-1} ampicillin and incubated overnight at 37 ˚C, 250 rpm. The overnight culture was transferred to an Erlenmeyer flask containing 1 L of LB medium supplemented with 100 μg mL^{-1} ampicillin and grown at 37 ˚C, 220 rpm until the optical absorbance at 600 nm reached 0.8 absorbance units. At this point, the culture was cooled in a water bath at room temperature for 20 minutes. Protein expression was induced by the addition of isopropyl β-D-1-thiogalactopyranoside (IPTG, 0.4 mM) and incubated at 16 ˚C for 18 hours. After expression, cells were harvested by centrifugation at 6,000 × g, 4 ˚C for 15 minutes and stored at -80 ˚C. For protein isolation, frozen cell pellets were thawed in ice, resuspended in Lysis Buffer (10 mM Tris-HCl, pH 8.0, 500 mM NaCl) and disrupted by sonication (Fisherbrand™ Q500 Sonicator fitted with standard 0.5-inch probe; Amp 30, 2-second pulse, 5-second pause, total sonication of 5 minutes). After sonication, the crude lysate was centrifuged at 18,000 × g for 30 minutes at 4 ˚C. Subsequently, the supernatant was filtered through a 0.45 μm syringe filter unit, and the resulting filtrate was passed through a previously equilibrated 1 mL Ni-NTA HisTrap FF column (GE Healthcare Life Sciences) using an ÄKTA Start protein purification system (GE Healthcare Life Sciences). The column was washed with Lysis Buffer containing 20 mM imidazole. The target protein was eluted with an imidazole gradient between 20 and 300 mM imidazole in Lysis Buffer, eluting as a single peak around 200 mM imidazole. Purified protein was buffer exchanged into Lysis Buffer using a 10-kDa ultra-filtration spin column (Amicon Ultra, Millipore, Billerica, MA). Aliquots of purified protein were flash-frozen in liquid nitrogen and stored at −80 ˚C until use.

Preparation of the heterogeneous biocatalyst (bio system) for H_2-driven cofactor recycling: All catalysts were prepared in a glovebox (Glove Box Technology) under a protective N_2 atmosphere (O_2 < 0.1 ppm), and in deoxygenated Tris-HCl buffer (100 mM, pH 8.0). Catalysts were not originally prepared in deuterated conditions but, when required, were exchanged into deuterated solution by centrifugation (10,000 × g, 5 min), removing the supernatant, and resuspending in an equal volume of ($^2H_5$)-Tris-^{2}HCl (100 mM, p^{2}H 8.0). The heterogeneous biocatalyst has previously been demonstrated to retain activity in the presence of low levels of O_2, and whilst batches were most commonly prepared under anaerobic conditions, they could be frozen and used outside of a glovebox as required.[1]

The heterogeneous catalyst system (depicted in Supplementary Figure 2) was prepared by sonication of a 20 mg mL^{-1} suspension of BP2000 carbon black in Tris-HCl buffer for 5 × 15 minutes (with agitation of the solution in between). Equal volumes of hydrogenase (4 mg mL^{-1}) and NAD$^+$ reductase (1.4 mg mL^{-1}) were then pre-mixed together, and added immediately to an aliquot of sonicated carbon suspension. After standing at 4 ˚C for 60 minutes, the solid catalyst was then removed from the preparatory solution by centrifugation (10,000 × g, 5 min) before being resuspended in deuterated or non-deuterated buffer as required. Enzyme loadings: hydrogenase loading of 39 pmoles per 100 µg of carbon, NAD$^+$ reductase loading of 65 pmoles per 100 μg of carbon.

The disappearance of the brown hue of the starting enzymatic solution, and the near-complete drop in detectable solution enzyme activity (as determined by standard benzyl viologen assays)[2] were taken as signs that virtually all of the enzymes were immobilized.

Heterogeneous biocatalytic system (bio system)

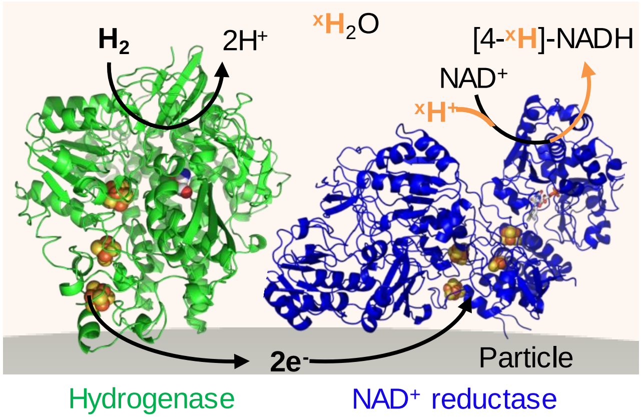

Supplementary Figure 2: Schematic representation of the heterogeneous biocatalyst system (bio system) for H_2-driven cofactor recycling (X = 1 or 2). The hydrogenase has a nickel-iron catalytic site, and the NAD^+ reductase has a flavin mononucleotide active site, and both enzymes possess a relay chain of iron-sulfur clusters to allow rapid electron transfer between the catalytic site and the surface of the protein.

Conducting and analysing ^{2}H-labelling reactions

Typical reaction conditions: Unless specified otherwise, all reactions were set up in a glovebox under a protective N_2 atmosphere (O_2 < 0.1 ppm) and were conducted on a 500 μL scale in sealed 0.5 mL micro-centrifuge tubes (Eppendorf) punctured with five holes in the lid (Ø 1.0 mm). The hydrogenase and NAD^+ reductase modified carbon particles (heterogeneous biocatalytic system) were added at a loading of 200–800 μg carbon mL^{-1}. Reaction solutions were pre-saturated with H_2 gas and the cofactor dissolved prior to the addition of the catalyst, to ensure that both hydrogenase and NAD^+ reductase enzymes were exposed to their respective substrates simultaneously. In order to help dissolve organic compounds ($^2H_6$)-dimethylsulfoxide was included in the reactions as required (1-5 vol%).

The punctured tubes were then transferred to a specially adapted shaker plate, which allowed for a steady flow of H_2 across the headspace (abbreviated as "atm H_2" throughout). Alternatively, if a pressurized H_2-atomsphere was required, a tinyclave steel pressure vessel (Buchi) was used, and rocked back and forth at 15 rpm whilst the reactions took place. In the case of volatile substrates, the H_2-saturated reaction solution was held in a sealed tube with a balloon to maintain the atmosphere.

Following the reaction, solid catalyst was removed by two rounds of centrifugation (10,000 × g, 5 min), and the solution was analysed by the methods described below. Conversions were calculated based upon the molar ratio of product(s) to substrate.

For each reaction, a control experiment in the absence of catalyst was prepared to ensure there was no background loss of reactant.

Overview of techniques for analysing ^{2}H-labelling reactions: A suite of analytical techniques was employed in order to ascertain the amount of desired deuterated product that been formed in each reaction:

- The extent of conversion was calculated by one or more of the following: ^{1}H NMR spectroscopy, GC-FID, HPLC, UV-vis spectroscopy.
- For chiral products, enantiomeric excess (*ee*) was determined by either chiral HPLC or chiral GC-FID.
- The %^{2}H incorporation was determined from ^{1}H NMR spectroscopic analysis (from the loss of signal intensity and collapse of coupling associated with incorporation of a ^{2}H unit) and/or GC-MS or LC/MS (from the changes in m/z value associated with installation of the heavy ^{2}H unit).

The specific details of such arguments are explained for each individual case in the following sections, with the associated reaction schemes and supporting data being shown in Supplementary Figures 3 - 58. The experimental procedures required to perform each of the analytical techniques were as follows:

- ***Procedure for analysing reactions using UV-vis spectroscopy***

Reactions which resulted in changes in the relative concentration of NAD^+/NADH could be followed be UV-vis spectroscopy. Firstly, solid particulates were removed from the solution by centrifugation (as described above) and the sample diluted in MilliQ (^1H_2O) water so that the cofactors were in the range 0.1–0.2 mM. A background spectrum of pure MilliQ was subtracted from that acquired for the sample. Measurements were made in a quartz cuvette (path length 1 cm, Hellma) on a Cary 60 UV/Vis spectrophotometer (Agilent).

- ***General procedure for analysing reactions by ^{1}H NMR spectroscopy***

Following removal of the catalyst particles by centrifugation, 450 μL of the sample solution was transferred to a Norell® SelectSeries™ 5 mm 400 MHz NMR sample tube. A further 50 μL of ^2H_2O was added to make up the volume to 500 μL and, in the case of non-deuterated solutions, for field locking purposes.

^{1}H NMR spectroscopy was carried out on either a Bruker Avance III HD nanobay (400 MHz) or Bruker Avance III (500 MHz), with samples for direct comparison always being run on the same machine. Both machines were equipped with 5 mm z-gradient broadband multinuclear probes.

Spectra were acquired according to the parameters in Supplementary Table 1. In the first instance, the Bruker *proc_1d* or *proc_1dakps* processing algorithms were applied, followed by additional manual re-phasing where necessary. A multipoint baseline correction was also applied across the entirety of the spectral window and a line broadening corresponding to between 0.3–1.0 Hz was applied to each spectrum to improve the S/N ratio. Signals were referenced against an appropriate signal: acetone (δ = 2.22 ppm), ($^2H_5$)-dimethylsulfoxide (δ = 2.71), or the peak arising from the Tris buffer (δ = 3.70 ppm at pH 8.0), all of which were referenced originally to 4,4-dimethyl-4-silapentane-1-sulfonic acid (δ = 0.00 ppm).

Integration of peaks arising from substrate and product could be used to judge the degree of conversion in cases where intensity was not diminished from ^{2}H-incorporation (such as aromatic protons). Similarly, comparison of the intensity of signals arising from the cofactor, buffer, and/or co-solvent could all be used to verify that a mass balance had been achieved in the conversion of substrate to product.

Additional multinuclear NMR spectroscopic techniques were also employed in the analysis of isolated products, utilising the general parameters in Supplementary Table 1. Further specific information is reported in the case of each spectrum as required.

- ***General procedures for analysing reaction products by GC-FID and GC-MS***

GC-FID was carried out on a ThermoScientific Trace 1310. GC-MS was carried out on an Agilent 7890B GC coupled to an Agilent 7200 Accurate Mass Q-ToF MS operating under EI mode. For both machines, injections were carried out by means of a robotic autosampler. The relevant columns and chromatography conditions are stated as required.

- ***General procedures for analysing reactions by HPLC-UV and HPLC-MS***

HPLC-UV was conducted on a Shimadzu UFLC LC-20AD Prominence liquid chromatograph equipped with a dual wavelength UV-spectrophotometric detector, and HPLC-MS was conducted on an Agilent 1260 system equipped with an Agilent 6120 B mass spectrometer utilising electrospray ionisation (ESI). MilliQ water and HPLC grade solvents were used throughout. The relevant columns and chromatography conditions are stated in each case as required.

- ***Additional techniques for solid state characterization of products***

Compounds isolated on a preparative scale were subjected to additional solid-state characterisation techniques. Elemental analysis was conducted on a Thermo (Carlo Erba) Flash 2000 Elemental Analyser, configured for %CHN. Mass spectrometry was conducted on an Agilent 6120 quadrupole LC-MS system with electrospray ionisation (ESI), direct injection (no column) and a flow rate of 2 mL min^{-1} (90 vol.% EtOH, 10 vol.% MilliQ, 0.1 vol.% formic acid). For high resolution mass spectrometry (HRMS) a Thermo Exactive Mass Spectrometer was used, calibrated on the day of analysis to a mass accuracy of ≤ 5 ppm. Thermogravimetric analysis to determine decomposition temperature was conducted on a Mettler Toledo TGA, with heating at 5 °C min^{-1} under air.

Conversion and characterisation of NAD^+ to [4*S*-^{2}H]-NADH

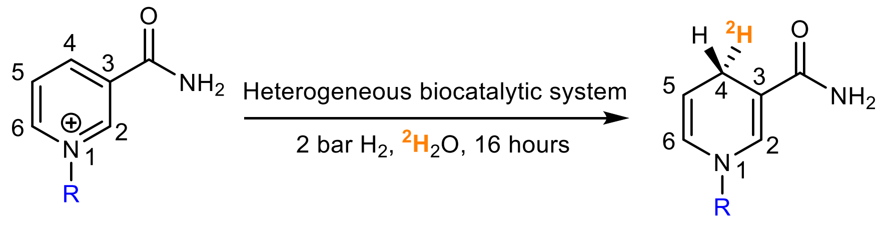

Supplementary Figure 3: Conversion of NAD^+ to deuterated-NADH. The H_2-driven biocatalytic system was used to reduce the oxidized form of the cofactor (NAD^+) to the reduced form bearing a deuterium atom in the 4-position of the nicotinamide ring ([4*S*-^{2}H]-NADH).

Experimental conditions: A sample of the heterogeneous biocatalytic system (100 μg), (prepared according to the previously described methods), was added to 500 μL of [$^2H_5$]-Tris^{2}HCl (100 mM, p^{2}H 8.0) containing 4 mM NAD^+, which was presaturated with $^1H_2$ gas. The reaction solution was sealed under 2 bar $^1H_2$ for 16 hours, whilst rocking at 30 rpm. Following removal of the solid particulates by centrifugation, the reaction was analysed ^{1}H NMR and UV-vis spectroscopies.

Characterisation: The ^{1}H NMR spectra of nicotinamide cofactors and their deuterated analogues were assigned according to well established literature arguments[3,4] using the peak(s) between 2.64–2.84 ppm (corresponding to the 4-position of the dihydronicotinamide ring). The spectrum of the NADH standard shows a multiplet with the expected splitting. The spectrum of the cofactor generated shows only a single peak in this region, demonstrating the introduction of a deuterium atom. The ratio of NAD^+ to NADH (and therefore extent of conversion) could be determined from the UV-vis spectra by measuring the ratio $A_{260\ nm}$: $A_{340\ nm}$, as described previously.[5] Analysis by ^{1}H NMR spectroscopy and UV-spectrophotometry together, indicated complete conversion of the cofactor to generate only [4-^{2}H]-NADH. By comparison of the ^{1}H NMR spectra to the literature, it can be deduced that the [4*S*-^{2}H]-form of the reduced cofactor is generated (determined from the chemical shift of the remaining proton, and the absence of a 3.5 Hz vicinal coupling).[3,4]

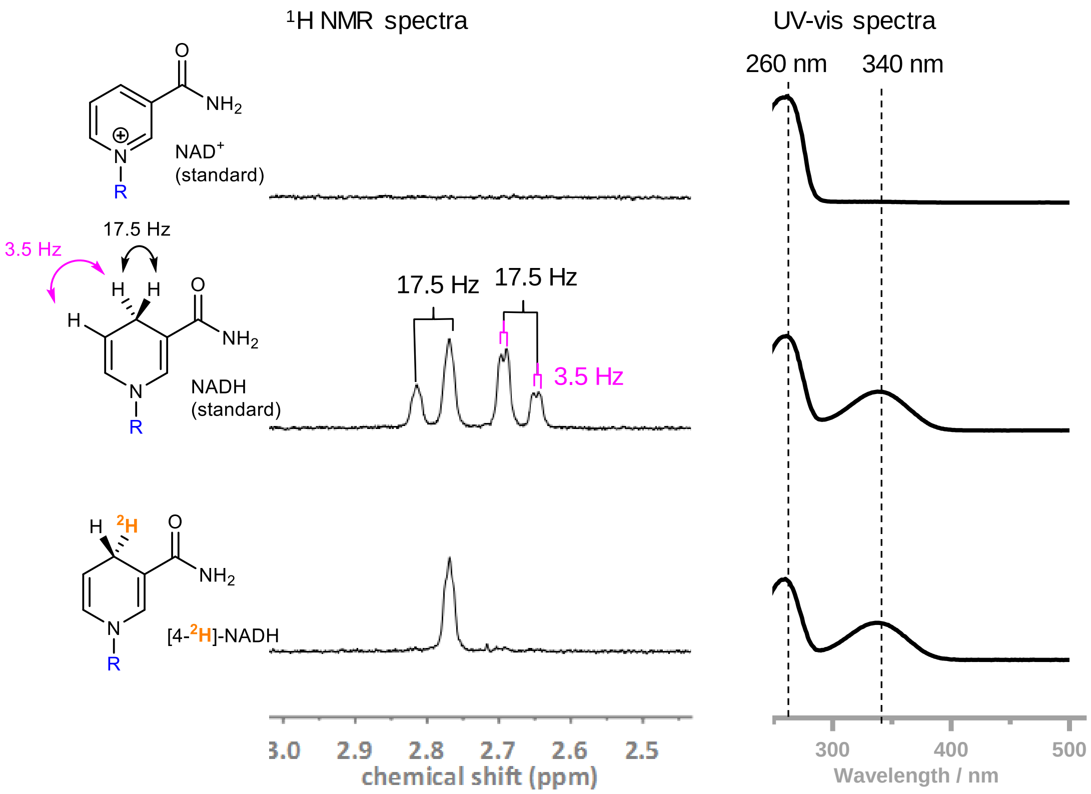

Supplementary Figure 4: Forming [4-^{2}H]-NADH by biocatalytic system in ^2H_2O. The figure shows the ^{1}H NMR and UV-visible spectroscopic analyses of the cofactor product generated by the H_2-driven heterogeneous biocatalytic system operating in ^2H_2O relative to commercial standards of NAD$^+$ and NADH. Only the diagnostic regions of the ^{1}H NMR spectra are shown (^2H_2O, p^{2}H 8.0, 400 MHz, 293 K).

Reductive deuteration of acetophenone (1a, 1b)

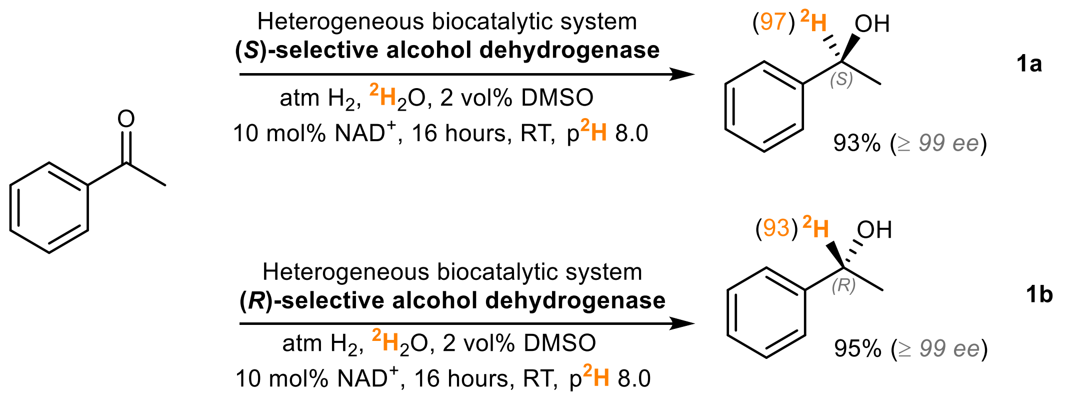

Supplementary Figure 5: Reductive deuteration of acetophenone. The H_2-driven biocatalytic system was used to convert acetophenone to (*S*)-[1-^{2}H]-1-phenylethanol (**1a**) and (*R*)-[1-^{2}H]-1-phenylethanol (**1b**)

<u>**Reaction conditions:**</u> Reaction was conducted in 0.5 mL of ($^2H_5$)-Tris-^{2}HCl (100 mM, p^{2}H 8.0) containing 5 mM acetophenone (AcPh), 2 vol% [$^2H_6$]-DMSO, 0.5 mM NAD$^+$, and 500 μg of (*S*)- or (*R*)-alcohol dehydrogenase (ADH) in solution. The mixture was presaturated with H_2 gas prior to addition of the heterogeneous bio system at a loading of 400 μg. The reactions were shaken at 500 rpm under a steady flow of H_2 for 16 hours.

<u>**Chiral HPLC:**</u> The *ee* of the 1-phenylethanol (1-PhEtOH) product was determined by chiral-phase HPLC, by comparison of the reaction mixture with relevant commercial standards. The results are depicted in Supplementary Figure 6. Reactions containing AcPh/PhEtOH were extracted with a 2 × volume of heptane:2-propanol (99:1 vol/vol), and then centrifuged at 18,800 × g for 5 minutes before being transferred to glass vials for HPLC according to the following conditions:
Column: Chiralpak IA column (15 cm × 4.6 mm, 5 μm particle size) with a 20 × 2.1 mm guard
Buffer: heptane : 2-propanol (99:1 vol/vol)
Column temperature = 40 ˚C
Flow rate = 1 mL min^{-1} (isocratic)
Injection volume = 10 μL
Detection = 210 nm

<u>**^{1}H NMR spectroscopy:**</u> After removal of the catalyst, the unmodified reaction solutions were analysed by ^{1}H NMR spectroscopy at 500 MHz and 298 K, with the results being shown in Supplementary Figure 7.

<u>**GC-MS:**</u> Following analysis by chiral HPLC (see above) the extracted reaction solutions were analysed by GC-MS to determine the extent of isotopic labelling. The results are depicted in Supplementary Figure 8. A commercial sample of 1-PhEtOH of natural isotopic abundance was extracted and analysed in an identical manner for comparison. The following conditions were used:

Column: DB-1701 (Agilent), 30 m length, 0.25 mm diameter, 0.25 μm (film thickness)
Carrier: He (CP grade), 100 kPa (constant pressure)
Inlet temperature: 250 ˚C

Injection gases: Split (25:1) split flow 30 mL min^{-1}, splitless time 0.7 min, purge 5 mL min^{-1}
Detection: Mass Spec, EI (70 eV), source temperature 230 ˚C. Scan range 50 – 650 amu with a scan rate of 5 Hz. Transfer line 300 ˚C
Oven heating profile:

0 → 5 mins	Hold at 50˚C
5 → 16.3 mins	Ramp to 275 ˚C at 20 ˚C min^{-1}
16.3 → 18.8 mins	Hold at 275 ˚C for 2.5 min

<u>**Analysis of Reactions 1a and 1b:**</u> The extent of reaction was calculated by ^{1}H NMR spectroscopy, by comparing the integrals of peaks arising from the aromatic protons of AcPh with those of 1-PhEtOH (Supplementary Figure 7). Here it was found that the conversion was very high (> 93%) in both. Chiral-phase HPLC demonstrated that only a single enantiomer had been formed in each case, with the (*R*)-ADH giving only (*R*)-1-PhEtOH (RT = 11.9 min), and (*S*)-ADH giving only (*S*)-1-PhEtOH (RT = 12.6 min), within the limits of detection (Supplementary Figure 6).

The site and extent of ^{2}H labelling in the 1-PhEtOH product was determined through a combination of ^{1}H NMR spectroscopy and GC-MS (Supplementary Figure 7 and Supplementary Figure 8). In the ^{1}H NMR spectrum, the signal corresponding to the –CH_3 protons could be used to detect ^{2}H labelling on the α-carbon, giving a doublet (δ = 1.47 ppm, J = 6.3 Hz) when a ^{1}H nucleus was attached to the α-carbon, and a broad singlet (δ = 1.46 ppm) when a ^{2}H moiety was present. Similarly, a quartet (δ = 4.91 Hz, J = 6.3 Hz) corresponding to the α-^{1}H signal was present in non-deuterated samples, but absent in those with a ^{2}H-label. Low levels of deuterium exchange on the –CH_3 group to give –$CH_2{}^{2}H$ was also evidenced by a small, broad shoulder with δ = 1.44 ppm (further ^{2}H exchange on the –CH_3 groups was not detected). Deconvolution of these peaks could be used to determine the relative proportion of each species in solution. In the mass spectrum of the 1-PhEtOH product extracted from the GC-MS trace, m/z values between 107–109 (corresponding to the [$C_7H_7O^{+\bullet}$] fragment) could also be used to determine the extent of ^{2}H incorporation at the α-position (Supplementary Figure 8). Similarly, the signals corresponding to the molecular ion (m/z 120–122) could be used to determine deuteration occurring on the methyl carbon. Whilst results arising from both the NMR and MS measurements were generally in good agreement, the latter was found to be more accurate for determining ^{1}H:^{2}H at the α-position, and the former more accurate for determining %^{2}H exchange on the –CH_3 group. In all experiments reported, the extent of secondary ^{2}H labelling on the β-carbon was less than 4% of the [α -^{2}H]-PhEtOH product.

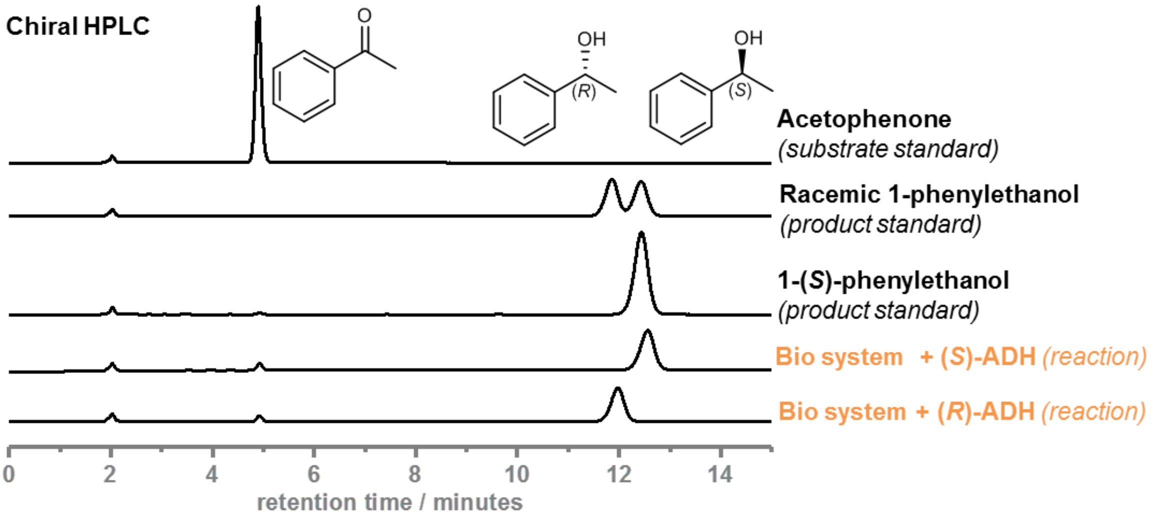

Supplementary Figure 6: Chiral HPLC-UV analysis of products of reductive deuteration of acetophenone. Comparison of the reaction products against standards of racemic 1-PhEtOH and (*S*)-1-PhEtOH demonstrates that only a single enantiomer is made by each biocatalyst.

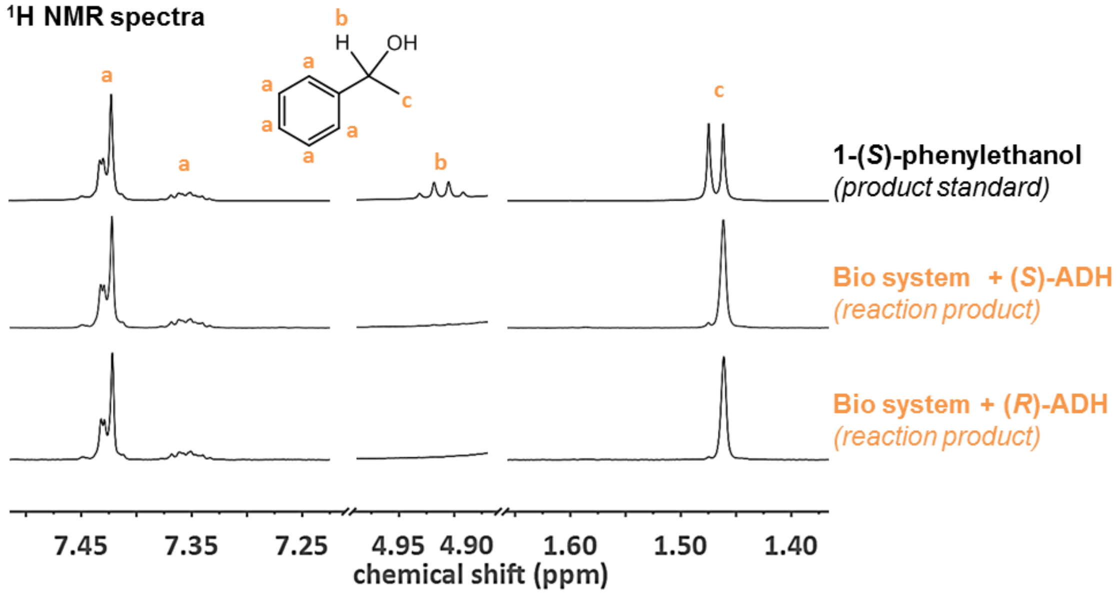

Supplementary Figure 7: ^{1}H NMR analysis of products of reductive deuteration of acetophenone (^2H_2O, p^{2}H 8.0, 500 MHz, 293 K). Comparison of the reaction products with a commercial standard of isotopically unenriched PhEtOH shows that high %^{2}H incorporation was achieved by both of the biocatalysts.

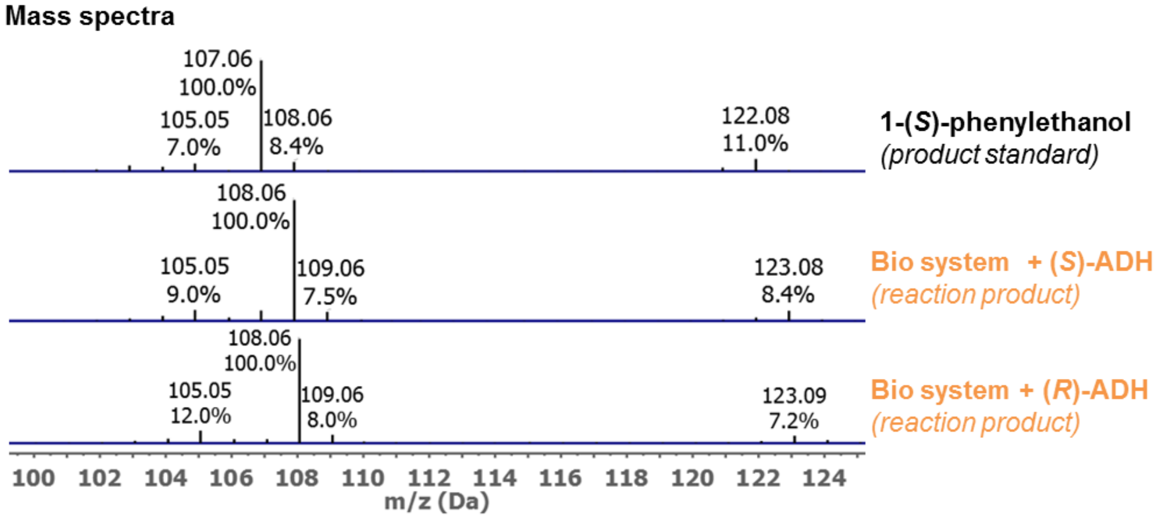

Supplementary Figure 8: Mass spectra of products of reductive deuteration of acetophenone (EI, 70 eV). The increase in molecular weight by +1.0 of the PhEtOH products (compared to a commercial standard of isotopically unenriched PhEtOH) demonstrates the expected ^{2}H incorporation.

Reductive deuteration of 4'-chloroacetophenone (1c, 1d)

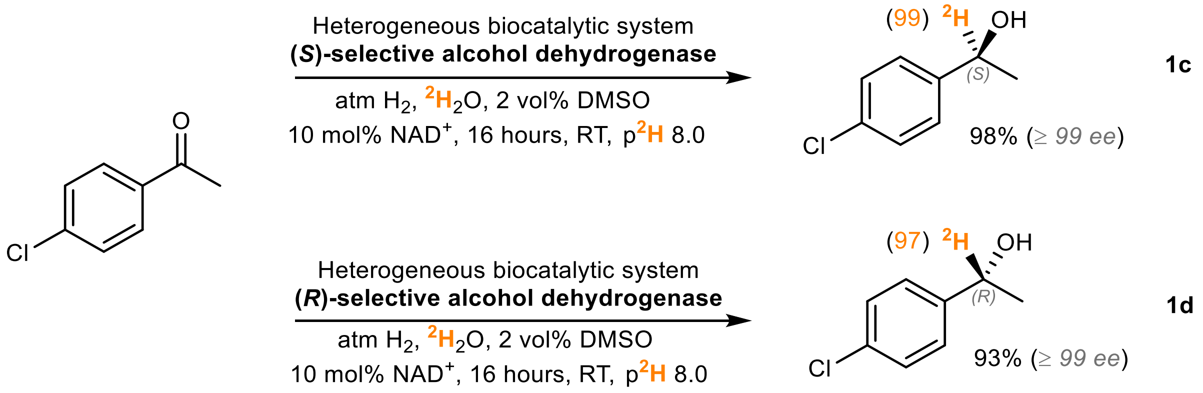

Supplementary Figure 9: Reductive deuteration of 4'chloroacetophenone. The H_2-driven biocatalytic system was used to convert 4'-chloroacetophenone to (*S*)-[1-^{2}H]-4'-chloro-1-phenylethanol (**1c**) or (*R*)-[1-^{2}H]-4'-chloro-1-phenylethanol (**1d**).

Reaction conditions: The reaction was conducted in 0.5 mL of ($^2H_5$)-Tris-^{2}HCl (100 mM, p^{2}H 8.0) containing 5 mM 4'-chloroacetophenone (Cl-AcPh), 2 vol% [$^2H_6$]-DMSO, 0.5 mM NAD$^+$, and 500 μg of (*S*)- or (*R*)-alcohol dehydrogenase (ADH) in solution. The mixture was presaturated with H_2 gas prior to addition of the heterogeneous biocatalytic system at a loading of 500 μg. The reactions were shaken at 500 rpm under a steady flow of H_2 for 16 hours. A racemic standard of the product 4'-chloro-1-phenylethanol was also prepared by shaking a 500 μL aqueous solution containing 5 mM Cl-AcPh and 2 vol% [$^2H_6$]-DMSO with $NaBH_4$ (5 mg).

Chiral GC: The *ee* of the 4'-chloro-1-phenylethanol (Cl-PhEtOH) product was determined by chiral-phase GC-FID, by comparison of the reaction mixture with a racemic standard and a commercial enantiomerically pure standard. The results are depicted in Supplementary Figure 10. Reactions containing Cl-AcPh/Cl-PhEtOH were extracted with a 2 × volume of EtOAc, and then centrifuged at 18,800 × g for 5 minutes before being transferred to glass vials for chiral GC-FID according to the following method:

Column: CP-Chirasil-Dex CB (Agilent), 25 m length, 0.25 mm diameter, 0.25 μm (film thickness), fitted with a guard of 10 m deactivated fused silica of the same diameter
Carrier: He (CP grade), 170 kPa (constant pressure)
Inlet temperature: 200 ˚C
Injection gases: Splitless with split flow 60 mL min^{-1}, splitless time 0.8 min, purge 5 mL min^{-1}
Injection volume: 0.1 μL
Detection: FID
Detector temperature: 250 ˚C
Detection gases: H_2 (35 mL min^{-1}), air (350 mL min^{-1}), makeup N_2 (40 mL min^{-1})
Oven heating profile:

0 → 5 mins	Hold at 70 ˚C
5 → 14.5 mins	Ramp to 165 ˚C at 10 ˚C min^{-1}
14.5 → 15.2 mins	Ramp to 200 ˚C at 50 ˚C min^{-1}
15.2 → 20.2 mins	Hold at 200 ˚C

^{1}H NMR spectroscopy: After removal of the catalyst, the unmodified reaction solutions were analysed by ^{1}H NMR spectroscopy at 500 MHz and 298 K, with the results being shown in Supplementary Figure 11.

LC-MS: The solutions prepared for NMR analysis were subsequently subjected to analysis by LC-MS to verify the isotopic labelling of the Cl-PhEtOH. The results are shown in Supplementary Figure 12. To the aqueous samples was added 50 vol.% MeCN, prior to analysis on an Agilent 1260 system equipped with an Agilent 6120 B mass spectrometer, according to the following method:

Column: Chromolith® Performance RP-18 endcapped 100-4.6
Buffers: A= MilliQ with 0.01 vol.% formic acid, B = MeCN with 0.01 vol.% formic acid
Column temperature = 40 ˚C
Detection = Electrospray ionisation (ESI) mass spectrometry
Flow rate = 1.5 mL min^{-1}
Flow profile:

0 → 7.5 mins	98 % A, 2 % B
7.5 → 8.5 mins	25 % A, 75 % B
8.5 → 9.0 mins	0 % A, 100 % B
9.0 → 10.0 mins	98 % A, 2 % B

Analysis of Reactions 1c and 1d: The extent of reaction was calculated by ^{1}H NMR spectroscopy, by comparing the integrals of peaks arising from the aromatic protons of Cl-AcPh with those of Cl-PhEtOH (Supplementary Figure 11). Here it was found that the conversion was very high (> 93%) in both experiments, which was further confirmed by GC-FID (Supplementary Figure 10). Chiral-phase GC-FID demonstrated that only a single enantiomer had formed in each case, with the (*R*)-ADH giving only (*R*)-Cl-PhEtOH (RT = 15.0 min), and (*S*)-ADH giving only (*S*)-Cl-PhEtOH (RT = 15.1 min), within the limits of detection.

Analysis of the ^{1}H NMR spectra of the product Cl-PhEtOH could be conducted in an identical manner to that described for PhEtOH previously (Supplementary Figure 11), with the non-aromatic peak shapes and positions remaining virtually identical. This analysis showed that the extent of ^{2}H labelling at the α-position was very high (> 97%) for both the (*R*)-Cl-PhEtOH and (*S*)-Cl-PhEtOH. Additional deuteration on the $-CH_3$ group by tautomeric exchange of the substrate with the solvent was found to be < 5%. Analysis by LC-MS (Supplementary Figure 12) showed the +1.0 mass shift associated with single deuteration of the Cl-PhEtOH product. Here, the signals of commercial natural-abundance Cl-PheEtOH found between m/z 139–142 (corresponding to the [$C_8H_8Cl^{+\bullet}$] fragment with the expected isotopic distribution arising from chlorine) are shifted to 140–143 in the isotopically labelled reaction products.

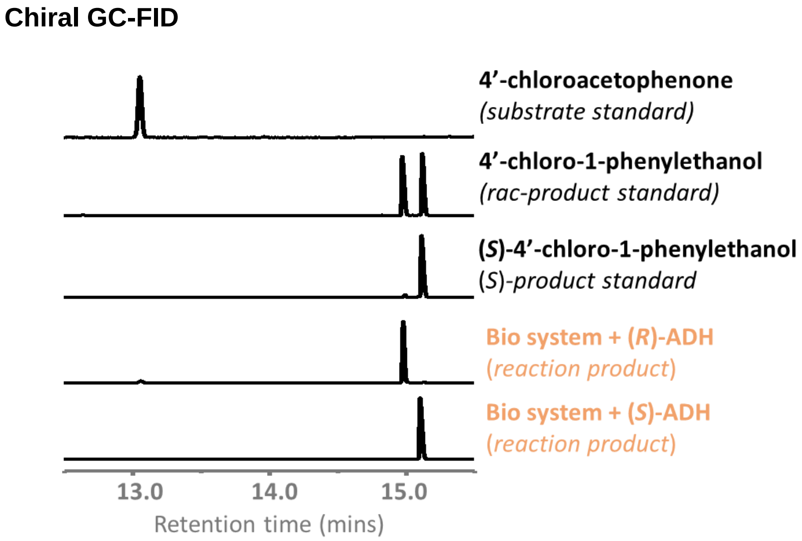

Supplementary Figure 10: Chiral GC-FID analysis of products of reductive deuteration of 4’-chloroacetophenone. Comparison of the reaction products against standards of racemic Cl-PhEtOH and (*S*)-Cl-PhEtOH demonstrates that only a single enantiomer is made by each biocatalyst.

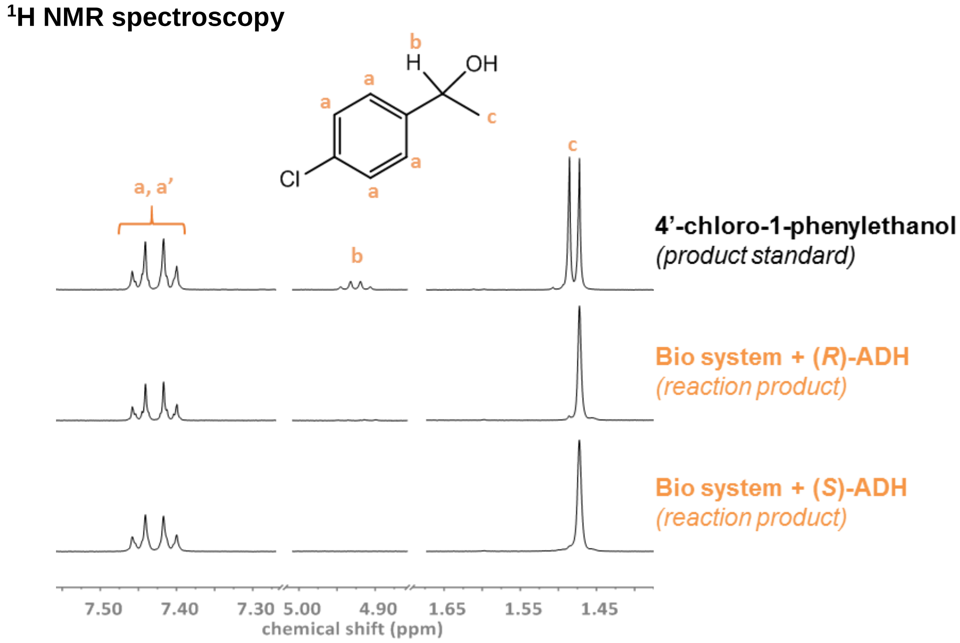

Supplementary Figure 11: ^{1}H NMR analysis of products of reductive deuteration of 4’chloroacetopheone (^2H_2O, p^{2}H 8.0, 500 MHz, 293 K). Comparison of the reaction products with a commercial standard of isotopically unenriched Cl-PhEtOH shows that high %^{2}H incorporation was achieved by both of the biocatalysts.

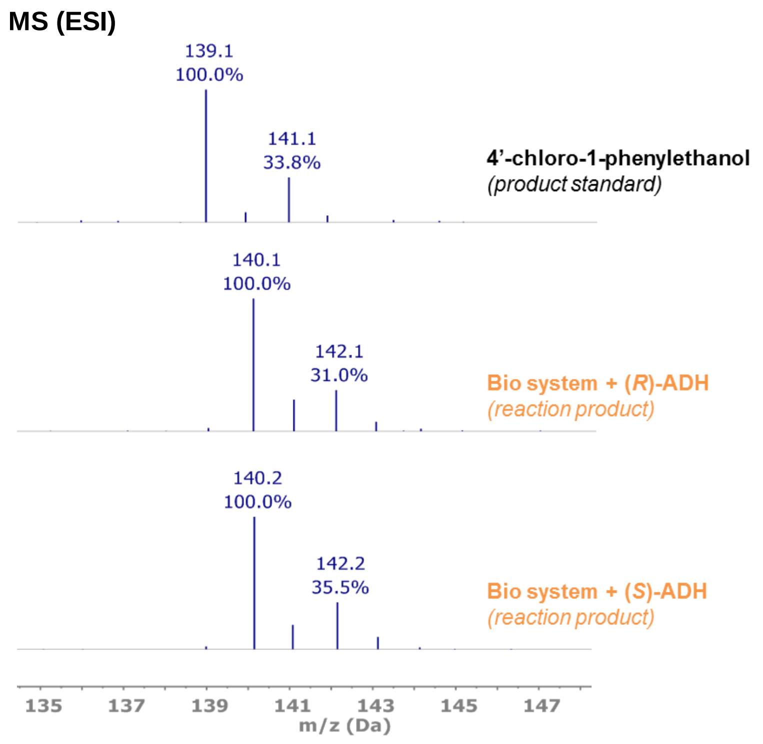

Supplementary Figure 12: Mass spectra (from LC-MS) of products of reductive deuteration of 4'-chloroacetophenone (ESI, positive mode). The increase in molecular weight by +1.0 of the biocatalytic reaction products (compared to a commercial standard of isotopically unenriched Cl-PhEtOH) demonstrates the expected ^{2}H incorporation.

Reductive deuteration of 4′-bromoacetophenone (1e, 1f)

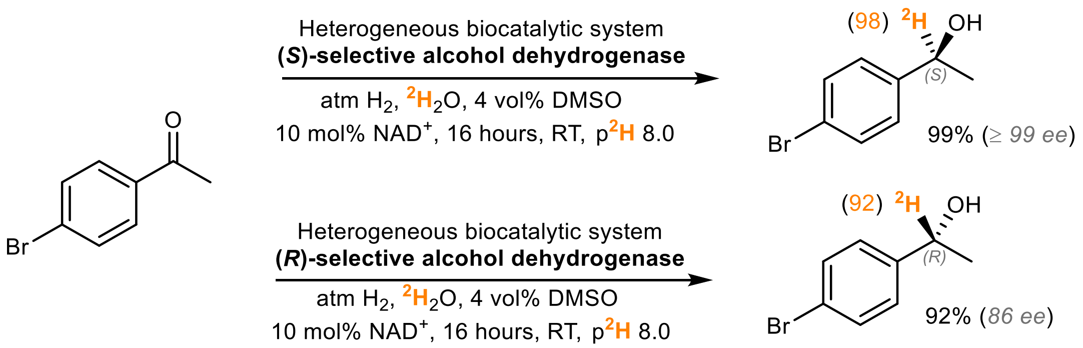

Supplementary Figure 13: Reductive deuteration of 4′-bromoacetophenone. The H_2-driven biocatalytic system was used to convert 4′-bromoacetophenone to (*S*)-[1-^{2}H]-4′-bromo-1-phenylethanol (**1e**) or (*R*)-[1-^{2}H]-4′-bromo-1-phenylethanol (**1f**)

Reaction conditions: The reaction was conducted in 0.5 mL of ($^2H_5$)-Tris-^{2}HCl (100 mM, p^{2}H 8.0) containing 5 mM 4′-bromoacetophenone (Br-AcPh), 4 vol% [$^2H_6$]-DMSO, 0.5 mM NAD$^+$, and 500 μg of (*S*)- or (*R*)-alcohol dehydrogenase (ADH) in solution. The mixture was presaturated with H_2 gas prior to addition of the heterogeneous biocatalytic system at a loading of 500 μg. The reactions were shaken at 500 rpm under a steady flow of H_2 for 16 hours. A racemic standard of the product 4′-bromo-1-phenylethanol was also prepared by shaking a 500 μL aqueous solution containing 5 mM Br-AcPh and 2 vol% [$^2H_6$]-DMSO with $NaBH_4$ (5 mg).

Analytic procedures: Analytical procedures were identical to those described for Cl-AcPh/Cl-PhEtOH in previously. The data arising from analysis by Chiral GC-FID, ^{1}H NMR spectroscopy, and LC-MS are depicted in Supplementary Figure 14, Supplementary Figure 15, and Supplementary Figure 16, respectively.

Analysis of Reactions 1e and 1f: The extent of reaction was calculated by ^{1}H NMR spectroscopy, by comparing the integrals of peaks arising from the aromatic protons of Br-AcPh with those of Br-PhEtOH (Supplementary Figure 15). Here it was found that the conversion was very high (> 92%) in both, which was further confirmed by GC-FID (Supplementary Figure 14). Chiral-phase GC-FID demonstrated that only a single enantiomer had been formed by the (*S*)-ADH giving a peak for only (*S*)-Br-PhEtOH (RT = 15.8 min). The (*R*)-ADH produced mostly (*R*)-Br-PhEtOH (RT = 15.7 min) as expected, but also produced a small amount of (*S*)-Br-PhEtOH, giving an *ee* of 86%. The diminished enantiomeric purity in this case was attributed to the increased size of the halogen on the aromatic ring.

Analysis of the ^{1}H NMR spectra of the product Br-PhEtOH could be conducted in an identical manner to that described for PhEtOH and Cl-PhEtOH previously, with the non-aromatic peak shapes and positions remaining virtually identical (Supplementary Figure 15). This analysis showed that the extent of ^{2}H labelling at the α-position was very high (> 92%) for both the (*R*)-Br-PhEtOH and (*S*)-Br-PhEtOH. Additional deuteration on the $-CH_3$ group by tautomeric exchange of the substrate with the solvent was found to be < 4% for the (*S*)-ADH, and around 10% for the (*R*)-ADH. Analysis by LC-MS (ESI) showed the +1.0 mass shift associated with single deuteration of the Br-PhEtOH product (Supplementary Figure 16). Here, the signals of commercial natural-abundance Br-PheEtOH found between m/z 183–186 (corresponding to the [$C_8H_8Br^{+\bullet}$] fragment with the expected isotopic distribution arising from bromine) are shifted to m/z 184–187 in the isotopically labelled reaction products.

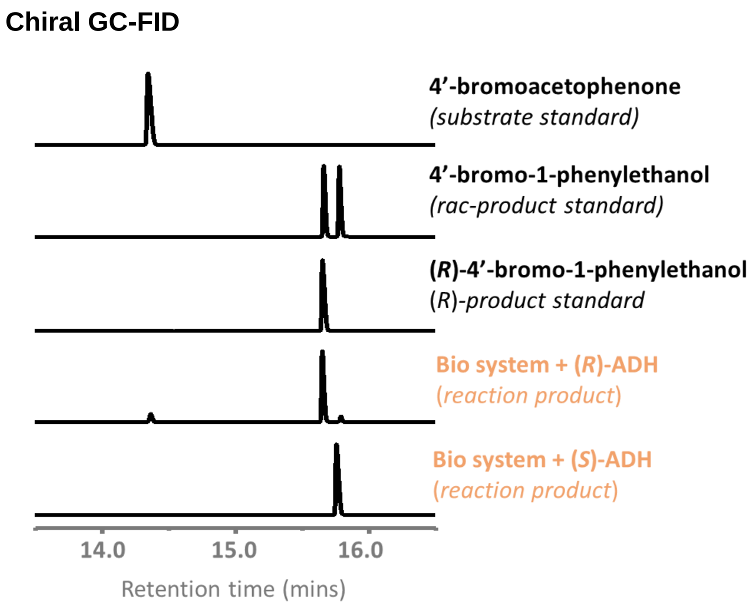

Supplementary Figure 14: Chiral GC-FID analysis of products of reductive deuteration of 4'-bromoacetophenone. Comparison of the reaction products against standards of racemic Br-PhEtOH and (*R*)-Br-PhEtOH demonstrates that only a single enantiomer is made by each biocatalyst.

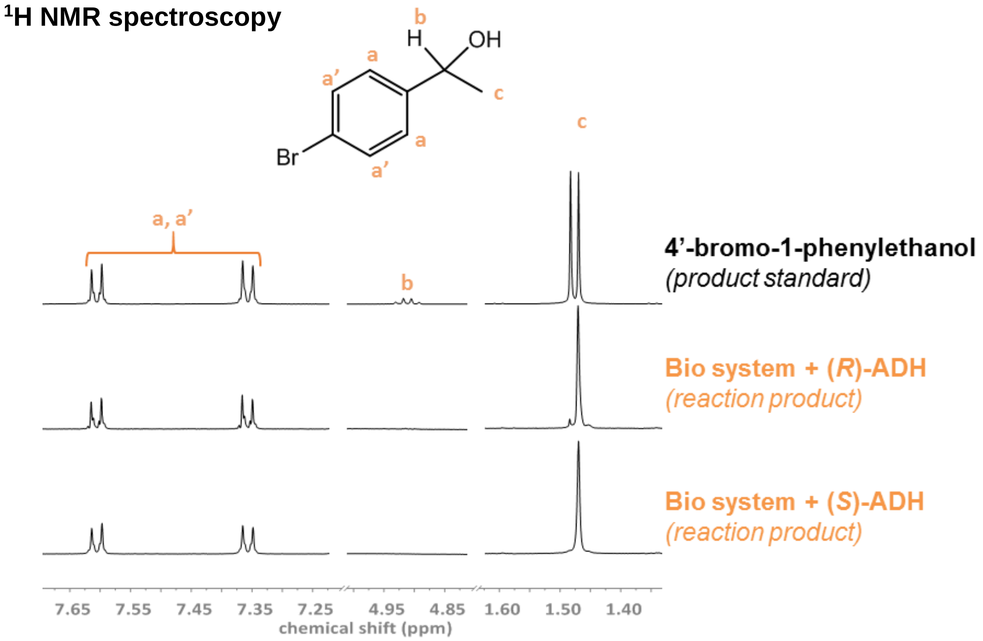

Supplementary Figure 15: ^{1}H NMR analysis of products of reductive deuteration of 4'-bromoacetopheone (^2H_2O, p^{2}H 8.0, 500 MHz, 293 K). Comparison of the reaction products with a commercial standard of isotopically unenriched Br-PhEtOH shows that high %^{2}H incorporation was achieved by both of the biocatalysts.

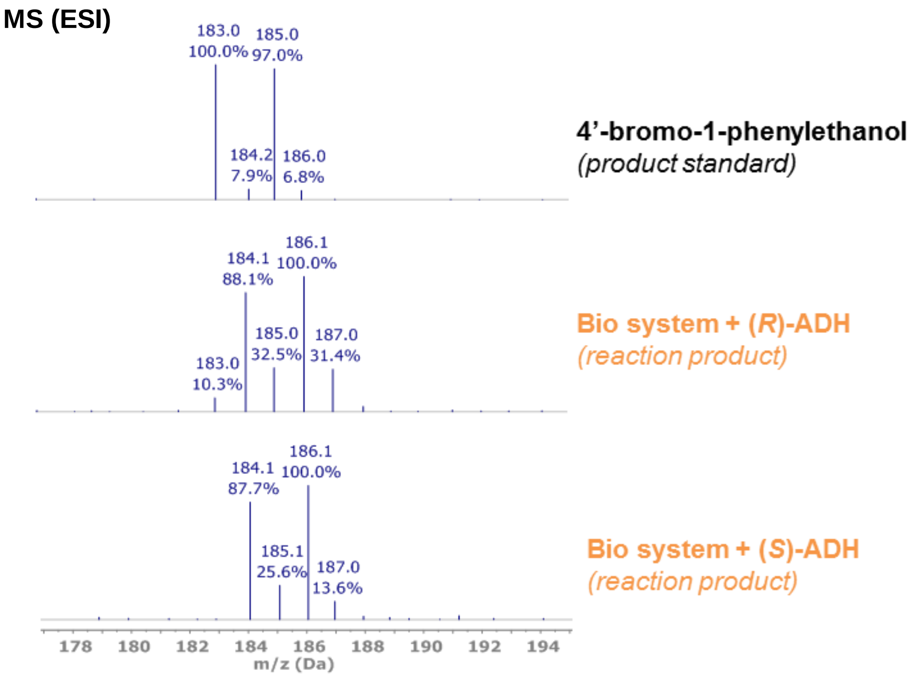

Supplementary Figure 16: Mass spectra (from LC-MS) of products of reductive deuteration of 4'-bromoacetophenone (ESI, positive mode). The increase in molecular weight by +1.0 of the biocatalytic reaction products (compared to a commercial standard of isotopically unenriched Br-PhEtOH) demonstrates the expected ^{2}H incorporation.

Reductive deuteration of pyruvate (2)

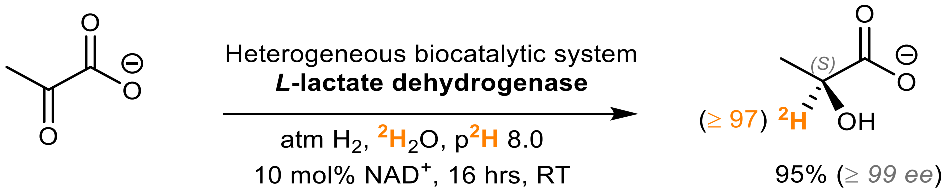

2

Supplementary Figure 17: Reductive deuteration of pyruvate. The H_2-driven biocatalytic system was used to convert pyruvate to (*L*)-[2-^{2}H]-lactate (**2**)

Reaction conditions: Reactions was conducted in 0.5 mL of ($^2H_{11}$)-Tris-^{2}HCl (100 mM, p^{2}H 8.0) containing 5 mM sodium pyruvate, 0.5 mM NAD$^+$, and 500 μg of *L*-lactate dehydrogenase (Merck). The mixture was presaturated with H_2 gas prior to addition of the heterogeneous biocatalytic system at a loading of 400 μg. The reactions were shaken at 500 rpm under a steady flow of H_2 for 16 hours.

GC-FID: In order to determine the conversion of pyruvate to lactate, the analytes were first derivatised and then subjected to analysis by GC-FID according to a modified version of the method of Bertrand *et al.*[6] The derivatisation scheme is shown in Supplementary Figure 18 and the results are depicted in Supplementary Figure 19. A sample of the reaction mixture (100 μL) was combined with 23 μL of EtOH and 13 μL of pyridine by means of a vortex mixer. Subsequently, 15 μL of ethyl chloroformate was added, and the reaction allowed to proceed until no further CO_2 was evolved. The derivatised pyruvate/lactate was extracted with 100 μL of C^2HCl_3, and transferred to a glass vial for analysis by GC according to the following method:

Column: DB-1701 (Agilent), 30 m length, 0.25 mm diameter, 0.25 μm film thickness
Carrier: He (CP grade), 1.1 mL minute (constant flow)
Inlet temperature: 250 ˚C
Injection gases: Splitless with split flow 50 mL min^{-1}, splitless time 0.7 min, purge 5 mL min^{-1}
Injection volume: 0.5 μL
Detection: FID
Detector temperature: 200 ˚C
Detection gases: H_2 (35 mL min^{-1}), air (350 mL min^{-1}), makeup N_2 (40 mL min^{-1})
Oven heating profile:

0 → 2 mins	Hold at 45˚C
2 → 15 mins	Ramp to 250 ˚C at 16 ˚C min^{-1}
15 → 20 mins	Hold at 250 ˚C for 5 min

Chiral GC-FID: The *ee* of the lactate product was determined by first derivatising with a chiral alcohol ((2*R*,3*R*)-(-)-2,3-butanediol) and then analysing the reaction solution by GC-FID according to a modified version of the method of Bertrand *et al.*[6] The derivatisation scheme is shown in Supplementary Figure 18 and the results are depicted in Supplementary Figure 19. A sample of the reaction mixture (100 μL) was combined with 35 μL of (2*R*,3*R*)-(-)-2,3-butanediol and 13 μL of pyridine by means of a vortex mixer. Subsequently, 15 μL of ethyl chloroformate was added, and the reaction allowed to proceed until no further CO_2 was evolved. Commercial standards of *L*- and *D*-lactate were similarly derivatised for comparison. The derivatised analytes were then extracted with 100 μL of C^2HCl_3, and transferred to a glass vial for analysis

by GC. The formation of diastereomeric products allowed for the analysis to be conducted on an achiral GC column according to the following method:

Column: DB-1701 (Agilent), 30 m length, 0.25 mm diameter, 0.25 μm film thickness
Carrier: He (CP grade), 170 kPa (constant pressure)
Inlet temperature: 230 ˚C
Injection gases: Splitless with split flow 50 mL min^{-1}, splitless time 0.7 min, purge 5 mL min^{-1}
Injection volume: 0.5 μL
Detection: FID
Detector temperature: 250 ˚C
Detection gases: H_2 (35 mL min^{-1}), air (350 mL min^{-1}), makeup N_2 (40 mL min^{-1})
Oven heating profile:

0 → 5 mins	Hold at 100˚C
5 → 30 mins	Ramp to 150 ˚C at 2 ˚C min^{-1}
30 → 35 mins	Hold at 150˚C for 5 minutes
35 → 41.5 mins	Ramp to 280 ˚C at 2 ˚C min^{-1}
41.5 → 46.5 mins	Hold at 280 ˚C for 5 minutes

GC-MS: Following analysis by GC-FID (see above) the EtOH/ethyl chloroformate derivatised reaction solutions were further analysed by GC-MS in order to determine the extent of isotopic labelling. A commercial sample of sodium lactate of natural isotopic abundance was derivatised and analysed in an identical manner for comparison, with the results depicted in Supplementary Figure 20. The GC-MS analysis was conducted according to the following method:

Column: DB-1701 (Agilent), 30 m length, 0.25 mm diameter, 0.25 μm (film thickness)
Carrier: He (CP grade), 100 kPa (constant pressure)
Inlet temperature: 250 ˚C
Injection gases Split (10:1) with split flow 12 mL min^{-1}, splitless time 0.7 min, purge 5 mL min^{-1}
Detection: Mass Spec, EI (70 eV), source temperature 230 ˚C. Scan range 50 – 650 amu with a scan rate of 5 Hz. Transfer line 300 ˚C
Oven heating profile:

0 → 5 mins	Hold at 50˚C
5 → 16.3 mins	Ramp to 275 ˚C at 20 ˚C min^{-1}
16.3 → 18.8 mins	Hold at 275 ˚C for 2.5 min

^{1}H NMR spectroscopy: After removal of the catalyst, the unmodified reaction solutions were analysed by ^{1}H NMR spectroscopy at 500 MHz and 298 K, with the results being shown in Supplementary Figure 21.

Analysis of Reaction 2: The very high conversion of pyruvate to lactate was confirmed by GC-FID analysis of the derivatised reaction solution (Supplementary Figure 19). Here, the peak corresponding to the pyruvate starting material (detected as ethyl pyruvate, RT = 5.85 minutes) disappeared almost entirely in the bio-hydrogenation reaction compared to a control in which no catalyst was included. The appearance of a peak at RT = 12.55 minutes, corresponding to the derivatised form of lactate, provided confirmation that the reaction had proceeded to 95 % completion. Similarly, derivatisaition of *L*- and *D*- lactate with a second chiral alcohol affords

diastereomeric products with differing retention times (*L* = 25.5 min, *R* = 25.3 min. Hence, by comparison, it could be confirmed that the lactate produced in the biohydrogenation was entirely of the *L*-form, within the limits of detection (> 99%).

The site and extent of ^{2}H labelling on the *L*-lactate product was determined through a combination of GC-MS and ^{1}H NMR spectroscopy (Supplementary Figure 20 and Supplementary Figure 21). In the ^{1}H NMR spectrum, the signal corresponding to the $-CH_3$ protons could be used to detect ^{2}H labelling on the *α*-carbon of *L*-lactate, forming a doublet (δ = 1.34 ppm, J = 9.2 Hz) when a ^{1}H nucleus was at the *α*-position, and a broad singlet (δ = 1.34 ppm) when a ^{2}H was present. Similarly, a quartet (δ = 4.13 ppm, J = 8.7 Hz) corresponding to the *α*-^{1}H signal was present in non-deuterated samples, but absent in those with a ^{2}H-label. Deuterium exchange on the $-CH_3$ group to yield $-CH_2{}^2H$ was also evidenced by a smaller, broad shoulder at δ = 1.32 (although it is not possible to determine the extent of complete deuterium exchange of the $-CH_3$ group *via* this method). Analysis of the ethoxycarbonyl ethylester-derivatised lactate by mass spectroscopy gave a series of diagnostic peaks, notably the molecular ions at m/z = 190.1 [$C_8H_{15}O_5{}^{+\bullet}$] and m/z = 191.1 [$^2H$-$C_8H_{14}O_5{}^{+\bullet}$], which could be used to determine the extent of ^{2}H incorporation at the *α*-position as > 97%. The peak at m/z = 192.1 could also be used to estimate the extent of formation of [$^2H_2$-$C_8H_{13}O_5{}^{+\bullet}$], shown in Supplementary Figure 21(b).

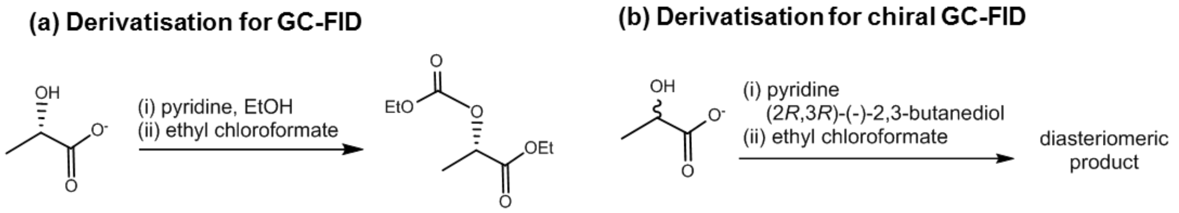

Supplementary Figure 18: Reactions schemes for the derivatisation of lactate for GC-FID analysis. (a) Ethyl chloroformate was used to produce an ethoxycarbonyl ethylester derivative for analysis by GC-FID. **(b)** A second procedure using a chiral alcohol was used to produce diastereomeric lactate derivatives that could be analysed on an achiral column.

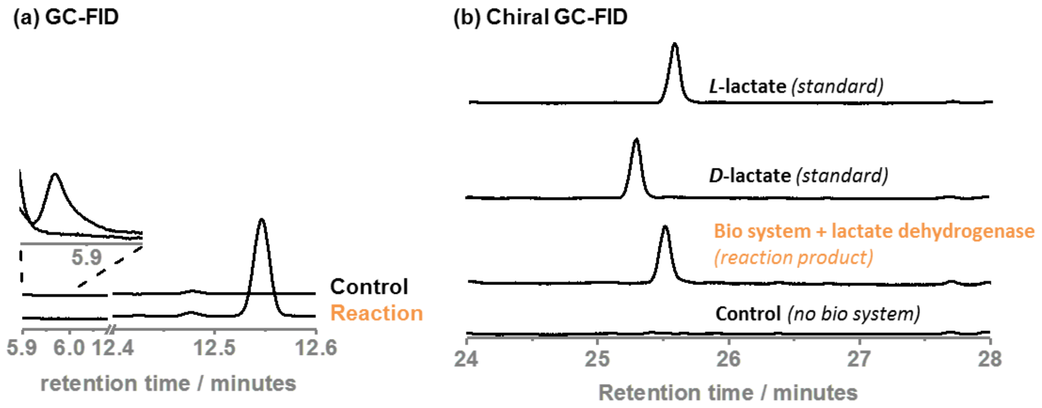

Supplementary Figure 19: GC-FID analysis of derivatised products from the reductive deuteration of pyruvate. **(a)** A peak at 5.85 minutes (pyruvate) is present in the control reaction but not in the reaction containing the biocatalyst. A peak at 12.55 minutes (lactate) is seen in the biocatalytic reaction, but not in the control. The presence/absence of these signals confirm the conversion of pyruvate to lactate by the biocatalyst. **(b)** Comparison of the reaction product against standards of *L*- and *D*-lactate demonstrate that only a single enantiomer is made by the biocatalyst.

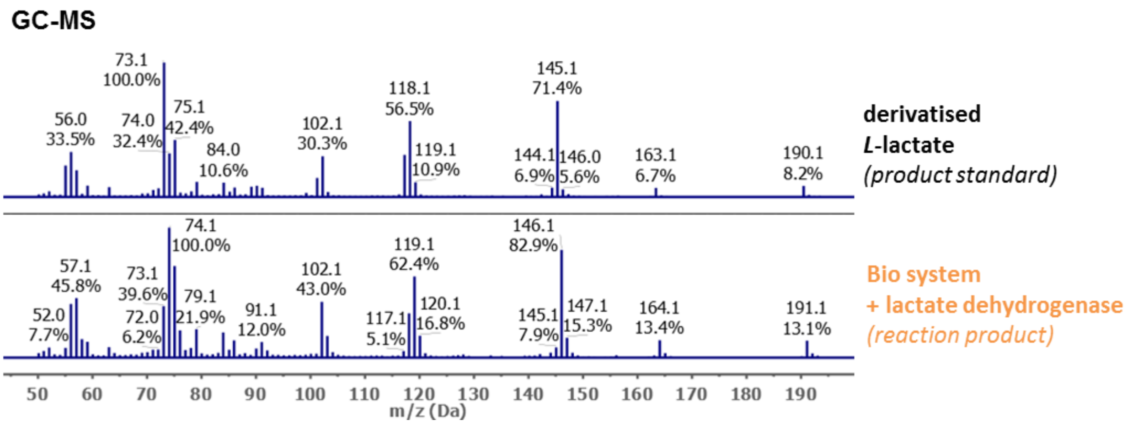

Supplementary Figure 20: Mass spectrum (from GC-MS) of the product of reductive deuteration of pyruvate (EI, 70 eV). The increase in molecular weight by +1.0 of the lactate product (compared to an isotopically unenriched commercial standard) demonstrates the expected ^{2}H incorporation by the biocatalyst.

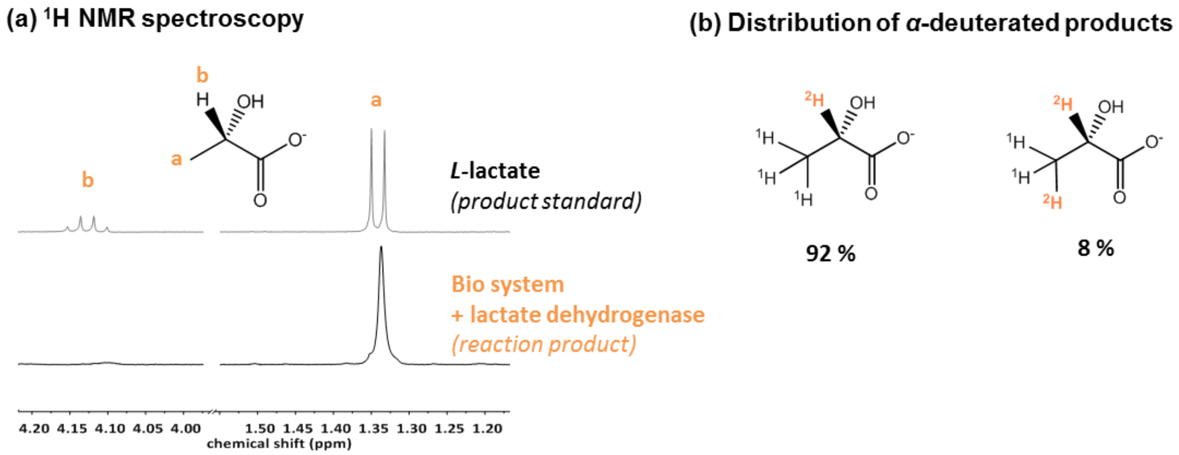

Supplementary Figure 21: ^{1}H NMR analysis of products of reductive deuteration of pyruvate ($^{2}H_2O$, p^{2}H 8.0, 500 MHz, 293 K). **(a)** Comparison of the reaction product with a commercial standard of isotopically unenriched lactate shows that high %^{2}H incorporation was achieved by the biocatalyst. **(b)** Distribution of lactate products deuterated in the *α*-position.

Reductive amination and deuteration of pyruvate (3)

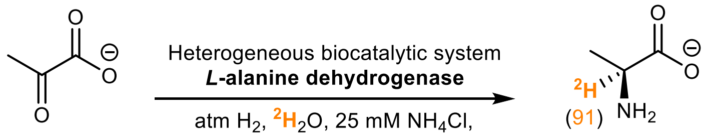

3

≥ 95% (≥ 99 *ee*)

Supplementary Figure 22: Reductive amination and deuteration of pyruvate. The H_2-driven biocatalytic system was used for the reductive amination and deuteration of pyruvate to form *L*-[2-^{2}H]-alanine (**3**).

Reaction conditions: Reaction was conducted in 0.5 mL of ($^2H_{11}$)-Tris-^{2}HCl (100 mM, p^{2}H 8.0) containing 5 mM sodium pyruvate, 25 mM NH_4Cl, 0.5 mM NAD^+, and 500 μg of *L*-alanine dehydrogenase in solution. The mixture was presaturated with H_2 gas prior to addition of the heterogeneous biocatalytic system at a loading of 400 μg. The reactions were shaken at 500 rpm under a steady flow of H_2 for 16 hours.

GC-FID: In order to determine the conversion of pyruvate to alanine, the analytes were first derivatised and then subjected to analysis by GC-FID according to a modified version of the method of Bertrand *et al.*[6] The derivatisation scheme is shown in Supplementary Figure 23 and the results are depicted in Supplementary Figure 24(a). A sample of the reaction mixture (100 μL) was combined with 23 μL of EtOH and 13 μL of pyridine by means of a vortex mixer. Subsequently, 15 μL of ethyl chloroformate was added, and the reaction allowed to proceed until no further CO_2 was evolved. The derivatised pyruvate/alanine was extracted with 100 μL of C^2HCl_3, and transferred to a glass vial for analysis by GC according to the following method:

Column: DB-1701 (Agilent), 30 m length, 0.25 mm diameter, 0.25 μm film thickness
Carrier: He (CP grade), 1.1 mL minute (constant flow)
Inlet temperature: 250 ˚C
Injection gases: Splitless with split flow 50 mL min^{-1}, splitless time 0.7 min, purge 5 mL min^{-1}
Injection volume: 1.0 μL
Detection: FID
Detector temperature: 200 ˚C
Detection gases: H_2 (35 mL min^{-1}), air (350 mL min^{-1}), makeup N_2 (40 mL min^{-1})
Oven heating profile:

0 → 2 mins	Hold at 45˚C
2 → 15 mins	Ramp to 250 ˚C at 16 ˚C min^{-1}
15 → 20 mins	Hold at 250 ˚C for 5 min

Chiral GC-FID: The same samples from the GC-FID analysis described above were then subjected to analysis on a chiral-phase GC column according to the method described below in order to determine the *ee* of the alanine product. Similarly derivatised commercial standards of *L*- and *D*-alanine standards were also run for comparison, with the results being depicted in Supplementary Figure 24(b). The GC method was as follows:

Column: CP-Chirasil-Dex CB (Agilent), 25 m length, 0.25 mm diameter, 0.25 μm (film thickness), fitted with a guard of 10 m deactivated fused silica of the same diameter
Carrier: He (CP grade), 170 kPa (constant pressure)
Inlet temperature: 200 ˚C
Injection gases: Splitless with split flow 60 mL min^{-1}, splitless time 0.8 min, purge 5 mL min^{-1}
Injection volume = 0.5 μL
Detection: FID
Detector temperature: 200 ˚C
Detection gases: H_2 (35 mL min^{-1}), air (350 mL min^{-1}), makeup N_2 (40 mL min^{-1})
Oven heating profile:

0 → 5 mins	Hold at 70˚C
5 → 30 mins	Ramp to 120 ˚C at 2 ˚C min^{-1}
30 → 36 mins	Ramp to 180 ˚C at 10 ˚C min^{-1}
36 → 41 mins	Hold at 180 ˚C for 5 minutes

GC-MS: The N-ethoxycarbonyl ethylester-derivatised samples from the GC-FID analysis described above were subsequently analysed by GC-MS to determine the extent of isotopic labelling. A commercial sample of alanine of natural isotopic abundance was derivatised and analysed in an identical manner for comparison, with the results depicted in Supplementary Figure 25. The GC-MS method employed was as follows:

Column: DB-1701 (Agilent), 30 m length, 0.25 mm diameter, 0.25 μm (film thickness)
Carrier: He (CP grade), 100 kPa (constant pressure)
Inlet temperature: 250 ˚C
Injection gases Split (25:1) with split flow 30 mL min^{-1}, splitless time 0.7 min, purge 5 mL min^{-1}
Detection: Mass Spec, EI (70 eV), source temperature 230 ˚C. Scan range 50 – 650 amu with a scan rate of 5 Hz. Transfer line 300 ˚C
Oven heating profile:

0 → 5 mins	Hold at 50˚C
5 → 16.3 mins	Ramp to 275 ˚C at 20 ˚C min^{-1}
16.3 → 18.8 mins	Hold at 275 ˚C for 2.5 min

^{1}H NMR spectroscopy: After removal of the catalyst, the unmodified reaction solutions were analysed by ^{1}H NMR spectroscopy at 500 MHz and 298 K, with the results being shown in Supplementary Figure 26(a).

Analysis of Reaction 3: The very high conversion of pyruvate to alanine was confirmed by GC-FID analysis of the derivatised reaction solution (Supplementary Figure 24(a)). Here, the peak corresponding to the pyruvate starting material (detected as ethyl pyruvate, RT = 5.95 minutes) disappeared almost entirely in the bio-hydrogenation reaction compared to a control in which no catalyst was included. The appearance of a peak at RT = 10.53 minutes, corresponding to the N-ethoxycarbonyl ethylester derivatised form of alanine, provided confirmation that the reaction had proceeded to 95 % completion. Similarly, derivatisation of commercial samples of *L*- and *D*-alanine and analysis on a chiral GC-column (Supplementary Figure 24(b)) gave rise to two peaks (*L* = 25.5 minutes, *D* = 25.3 minutes). Hence, by comparison, it could be confirmed that the alanine produced in the biohydrogenation was entirely of the *L*-form, within the limits of detection (> 99 %).

The site and extent of ^{2}H labelling on the *L*-alanine product was determined through a combination of GC-MS and ^{1}H NMR spectroscopy (Supplementary Figure 25 and Supplementary Figure 26). In the ^{1}H NMR spectrum, the signal corresponding to the $–CH_3$ protons could be used to detect ^{2}H labelling on the α-carbon of *L*-alanine, forming a doublet (δ = 1.47 ppm, J = 7.4 Hz) when a ^{1}H nucleus was at the α-position, and a broad singlet (δ = 1.46 ppm) when a ^{2}H was present. Similarly, a quartet (δ = 3.77 Hz, J = 7.4 Hz) corresponding to the α-^{1}H signal was present in non-deuterated samples, but absent in those with a ^{2}H-label. Integration of the relevant peaks indicated a ^{2}H-incorporation of 97 % on the α-carbon. Single and double deuterium exchange on the $–CH_3$ group to yield $–CH_2{}^{2}H$ and $CH^{2}H_2$ was also evidenced by smaller, broad shoulders at δ = 1.44 and δ = 1.43 ppm respectively (although it was not possible to quantify the extent of $–C^{2}H_3$ formation *via* this method). Analysis by mass spectroscopy gave rise to a high intensity peak corresponding to the derivatised alanine following cleavage of the N-ethoxycarbonyl group, with m/z = 116.1 (corresponding to $[C_5H_{10}NO_2{}^{+\bullet}]$) and m/z = 117.1 (corresponding to $[^{2}H\text{-}C_5H_9NO_2{}^{+\bullet}]$). The peaks at m/z = 116.1 and 117.1 could therefore be used to verify the high %^{2}H incorporation at the α-position discerned previously by NMR spectroscopy. Similarly, the signals with m/z 118–120 could also be used to better quantify the extent of ^{2}H-exchange that had occurred on the $–CH_3$ carbon of the α-deuterated alanine, which was subsequently shown to be minimal Supplementary Figure 26(b).

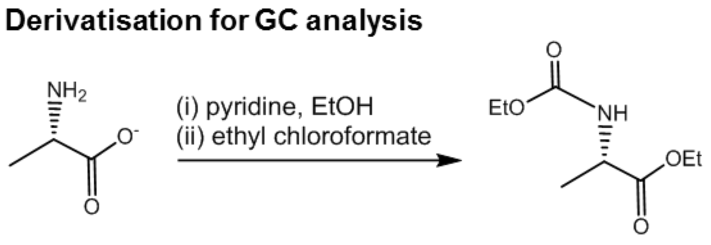

Supplementary Figure 23: Reaction scheme for the derivatisation of alanine for GC-FID analysis. Ethyl chloroformate was used to produce a carbamate derivative for GC-FID analysis.

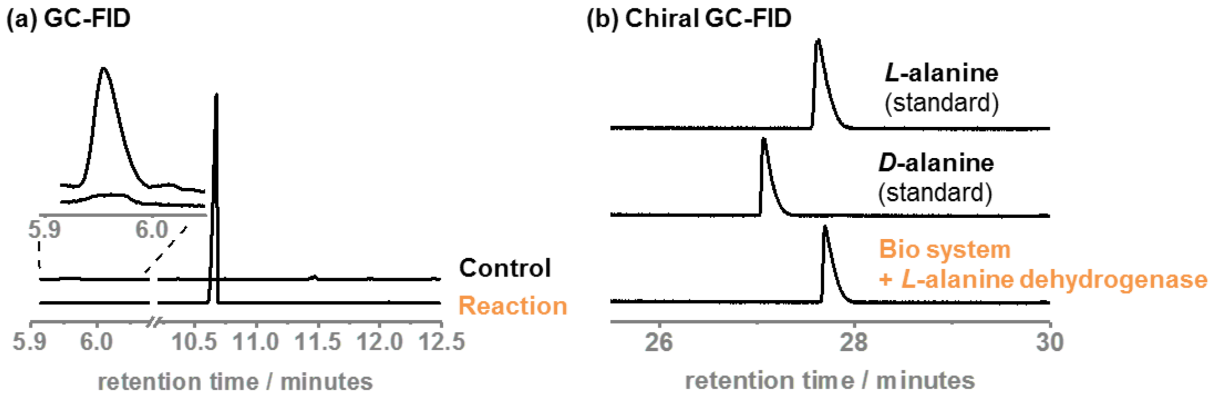

Supplementary Figure 24: GC-FID analysis of derivatised products from the reductive amination and deuteration of pyruvate. (a) A peak at 5.85 minutes (derivatised pyruvate) is present in the chromatogram of the control reaction but not in the chromatogram of the reaction containing the biocatalyst. A peak at 10.53 minutes (derivatised alanine) is seen in the chromatogram of the biocatalytic reaction, but not in the control. The presence/absence of these signals confirm the conversion of pyruvate to alanine by the biocatalyst. **(b)** Comparison of the reaction product against standards of *L*- and *D*-alanine demonstrate that only a single enantiomer is made by the biocatalyst.

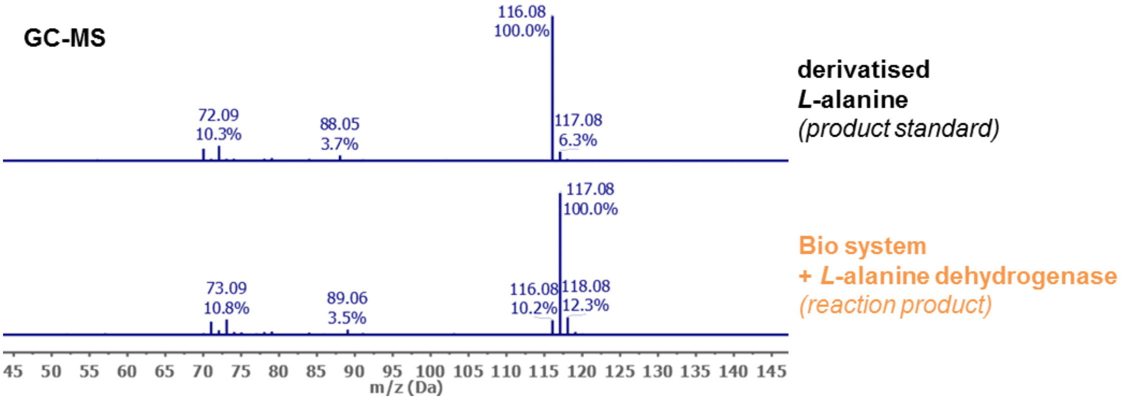

Supplementary Figure 25: Mass spectrum (from GC-MS) of the product of reductive amination and deuteration of pyruvate (EI, 70 eV). The increase in molecular weight by +1.0 of the alanine product (compared to an isotopically unenriched commercial standard) demonstrates the expected ^{2}H incorporation by the biocatalyst.

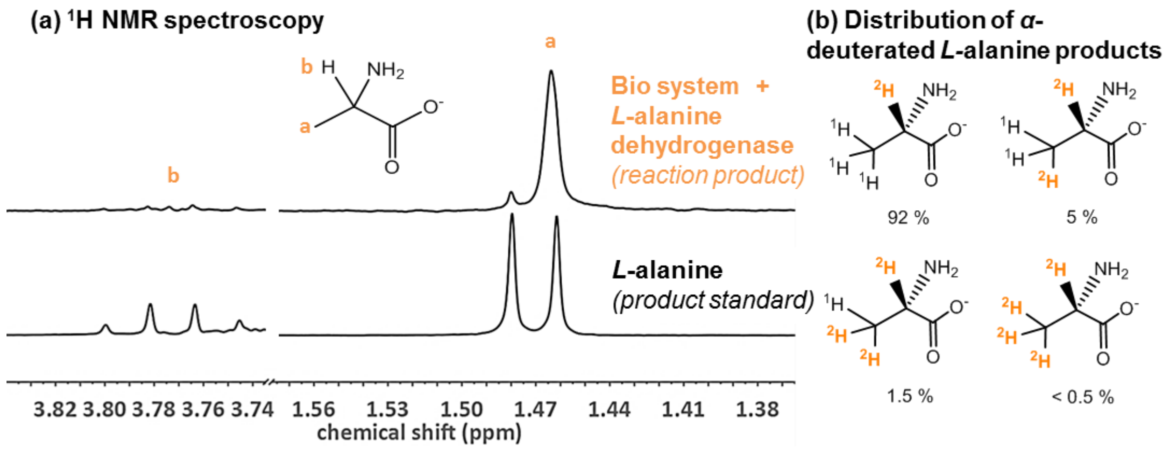

Supplementary Figure 26: ^{1}H NMR analysis of products of reductive amination and deuteration of pyruvate (^2H_2O, p^{2}H 8.0, 500 MHz, 293 K). **(a)** Comparison of the reaction product with a commercial standard of isotopically unenriched alanine shows that high %^{2}H incorporation was achieved by the biocatalyst. **(b)** Distribution of lactate products deuterated in the α-position.

Reductive amination and deuteration of phenylpyruvate (4)

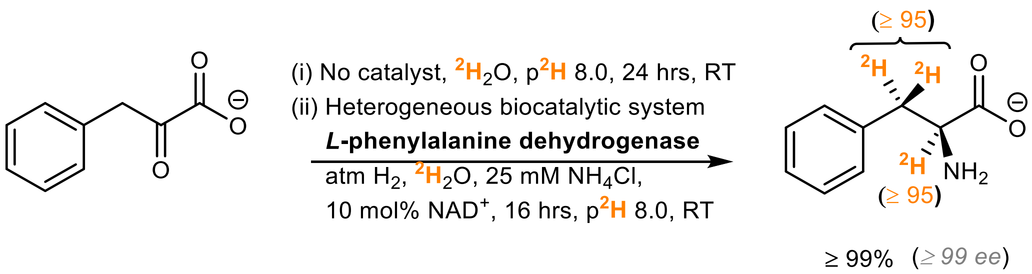

4

Supplementary Figure 27: Reductive amination and deuteration of phenylpyruvate. The H_2-driven biocatalytic system was used for the reductive amination and deuteration of phenylpyruvate to form [2,2,3-$^2H_3$]-phenylalanine (**4**).

Reaction conditions: Phenylpyruvic acid (5 mM) in 0.5 mL of ($^2H_{11}$)-Tris-^{2}HCl (100 mM, p^{2}H 8.0) was first allowed to stand for 24 hours in order to exchange labile protons for deuterons, before the addition of 25 mM NH_4Cl, 0.5 mM NAD^+, and 500 μg of *L*-phenylalanine dehydrogenase. The mixture was presaturated with H_2 gas prior to addition of the biocatalyst systems at a loading of 400 μg. The reaction was then shaken at 500 rpm under a steady flow of H_2 for 16 hours.

GC-FID: In order to determine the conversion of phenylpyruvate to alanine, the analytes were first derivatised and then subjected to analysis by GC-FID according to a modified version of the method of Bertrand *et al.*[6] The derivatisation scheme is shown in Supplementary Figure 28(a) and the results are depicted in Supplementary Figure 28(b). A sample of the reaction mixture (100 μL) was combined with 23 μL of EtOH and 13 μL of pyridine by means of a vortex mixer. Subsequently, 15 μL of ethyl chloroformate was added, and the reaction allowed to proceed until no further CO_2 was evolved. The derivatised pyruvate/alanine was extracted with 100 μL of C^2HCl_3, and transferred to a glass vial for analysis by GC according to the following method:

Column: DB-1701 (Agilent), 30 m length, 0.25 mm diameter, 0.25 μm film thickness
Carrier: He (CP grade), 170 kPa (constant pressure)
Inlet temperature: 230 ˚C
Injection gases: Splitless with split flow 50 mL min^{-1}, splitless time 0.7 min, purge 5 mL min^{-1}
Injection volume: 0.5 μL
Detection: FID
Detector temperature: 250 ˚C
Detection gases: H_2 (35 mL min^{-1}), air (350 mL min^{-1}), makeup N_2 (40 mL min^{-1})
Oven heating profile:

0 → 5 mins	Hold at 100˚C
5 → 30 mins	Ramp to 150 ˚C at 2 ˚C min^{-1}
30 → 35 mins	Hold at 150˚C for 5 minutes
35 → 41.5 mins	Ramp to 280 ˚C at 20 ˚C min^{-1}
41.5 → 46.5 mins	Hold at 280 ˚C for 5 minutes

GC-MS: The N-ethoxycarbonyl ethylester-derivatised samples from the GC-FID analysis described (see above) were subsequently analysed by GC-MS in order to determine the extent of the isotopic labelling. A commercial sample of *L*-phenylalanine of natural isotopic abundance was derivatised and analysed in an identical manner for comparison, with the results depicted in Supplementary Figure 30. The GC-MS method employed was as follows:

Column: DB-1701 (Agilent), 30 m length, 0.25 mm diameter, 0.25 μm (film thickness)
Carrier: He (CP grade), 100 kPa (constant pressure)
Inlet temperature: 250 ˚C
Injection gases Split (10:1) with split flow 12 mL min^{-1}, splitless time 0.7 min, purge 5 mL min^{-1}
Detection: Mass Spec, EI (70 eV), source temperature 230 ˚C. Scan range 50 – 650 amu with a scan rate of 5 Hz. Transfer line 300 ˚C
Oven heating profile:

0 → 5 mins	Hold at 50˚C
5 → 16.3 mins	Ramp to 275 ˚C at 20 ˚C min^{-1}
16.3 → 18.8 mins	Hold at 275 ˚C for 2.5 min

Chiral GC-FID: Derivatisation of the samples for analysis by chiral GC-FID to establish the enantiomeric excess of the reaction, was carried out according to the procedure described in the previous section, except with *n*PrOH (30 μL) used in the place of EtOH, as shown by the reaction scheme in Supplementary Figure 29(a). The results are depicted in Supplementary Figure 29(b). The chiral GC-FID analysis was carried out using the following parameters:

Column: CP-Chirasil-Dex CB (Agilent), 25 m length, 0.25 mm diameter, 0.25 μm (film thickness), fitted with a guard of 10 m deactivated fused silica of the same diameter
Carrier: He (CP grade), 2 mL min^{-1} (constant flow)
Inlet temperature: 200 ˚C
Injection gases: Splitless with split flow 80 mL min^{-1}, splitless time 0.8 min, purge 5 mL min^{-1}
Injection volume = 0.3 μL
Detection: FID
Detector temperature: 200 ˚C
Detection gases: H_2 (35 mL min^{-1}), air (350 mL min^{-1}), makeup N_2 (40 mL min^{-1})
Oven heating profile:

0 → 5 mins	Hold at 70˚C
5 → 115 mins	Ramp to 180 ˚C at 1˚C min^{-1}
115 → 130 mins	Hold at 180 ˚C for 15 minutes

^{1}H NMR spectroscopy: After removal of the catalyst, the unmodified reaction solutions were analysed by ^{1}H NMR spectroscopy at 500 MHz and 298 K, with the results being shown in Supplementary Figure 31.

Analysis of Reaction 4: The very high conversion of phenylpyruvate to phenylalanine was confirmed by GC-FID analysis of the derivatised reaction solution (Supplementary Figure 28). Here, the peak corresponding to the derivatised phenylpyruvate starting material (RT = 15.05 minutes) disappeared almost entirely in the bio-hydrogenation reaction compared to a control in which no catalyst was included. The appearance of a peak (RT = 14.94 minutes) corresponding to the N-ethoxycarbonyl ethylester derivatised form of phenylalanine, provided confirmation that the reaction had proceeded to ≥ 99% completion.

Similarly, derivatisation of commercial standards of *L*- and *D*-phenylalanine and analysis on a chiral GC-column (Supplementary Figure 29) gave rise to two peaks (*L* = 91.78 minutes, *D* = 92.00 minutes). Hence, it could be confirmed that the alanine produced in the biohydrogenation was entirely of the *L*-form, within the limits of detection.

The site and extent of ^{2}H labelling on the *L*-phenylalanine product was determined through a combination of GC-MS and ^{1}H NMR spectroscopy (Supplementary Figure 30 and Supplementary Figure 31). In this case, the phenylpyruvate starting material was left in the reaction solution to allow exchange of the labile protons for deuterons in the solvent. Following reduction by the biocatalyst system and *L*-phenylalanine dehydrogenase, the resulting product was labelled on both α- and β-positions. Accordingly, the signals at δ = 4.01 ppm (dd, 1H, αH, J = 4.1 Hz, J = 6.2 Hz), δ = 3.31 ppm (dd, 1H, βH, J = 4.1 Hz, J = 11.7 Hz), and δ = 3.14 ppm (dd, 1H, βH, J = 6.4 Hz, J = 11.7 Hz) which were all observed in the commercial standard of *L*-phenylalanine, were found to be absent in the reaction product. Similarly, GC-MS analysis of the ethoxycarbonyl ethylester-derivative of phenylalanine demonstrated the expected +3.0 mass shift of the molecular mass from m/z 220.1 to 223.1.

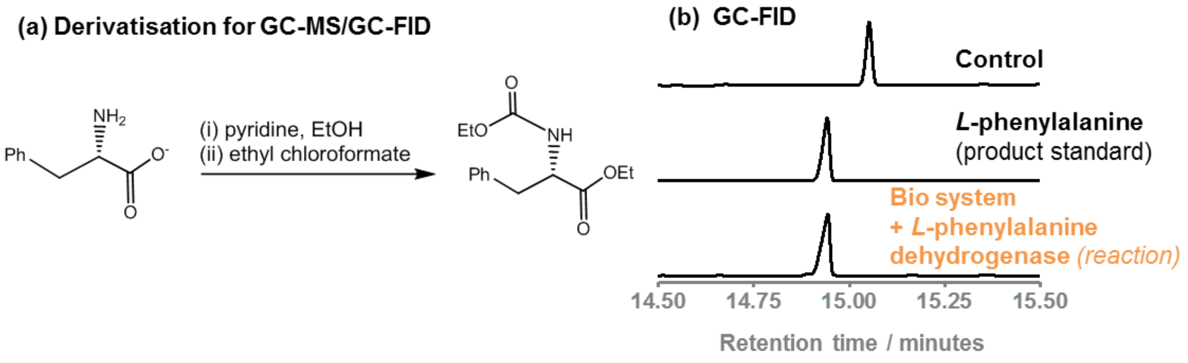

Supplementary Figure 28: Analysis of the reductive amination and deuteration of phenylpyruvate by GC. (a) Reaction mixtures were derivatised by treatment with ethyl chloroformate to facilitate analysis by GC. **(b)** A peak at 15.05 minutes (derivatised phenylpyruvate) is present in the chromatogram of the control reaction but not in the reaction containing the biocatalyst. A peak at 14.94 minutes (derviatised phenylalanine) is seen for the biocatalytic reaction, but not for the control. The presence/absence of these signals confirm the conversion of phenylpyruvate to phenylalanine by the biocatalyst.

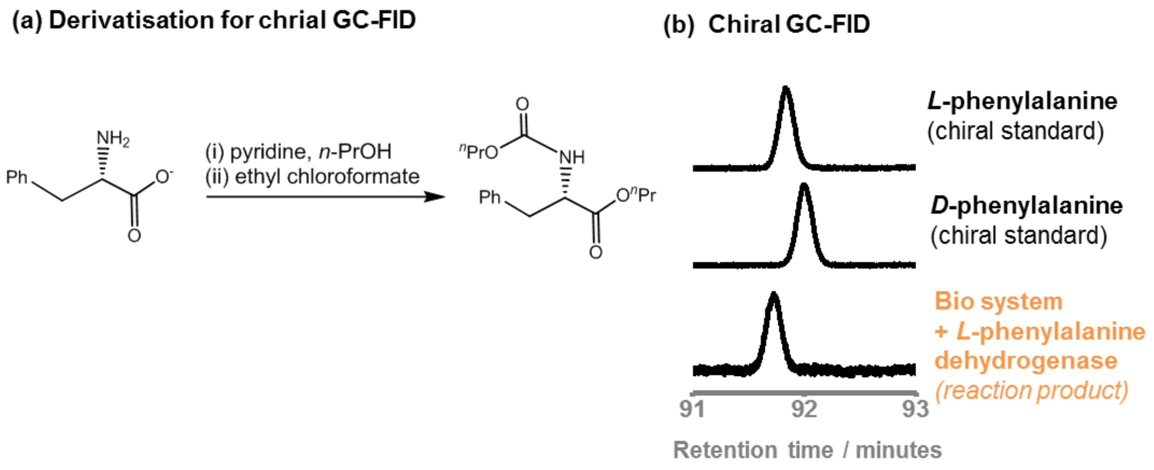

Supplementary Figure 29: Analysis of the reductive amination and deuteration of phenylpyruvate by chiral GC-FID. (a) Reaction mixtures were derivatised by treatment with ethyl chloroformate to facilitate analysis by chiral GC-FID. **(b)** Comparison of the reaction product against standards of *L*- and *D*-phenylalanine demonstrate that only a single enantiomer is made by the biocatalyst.

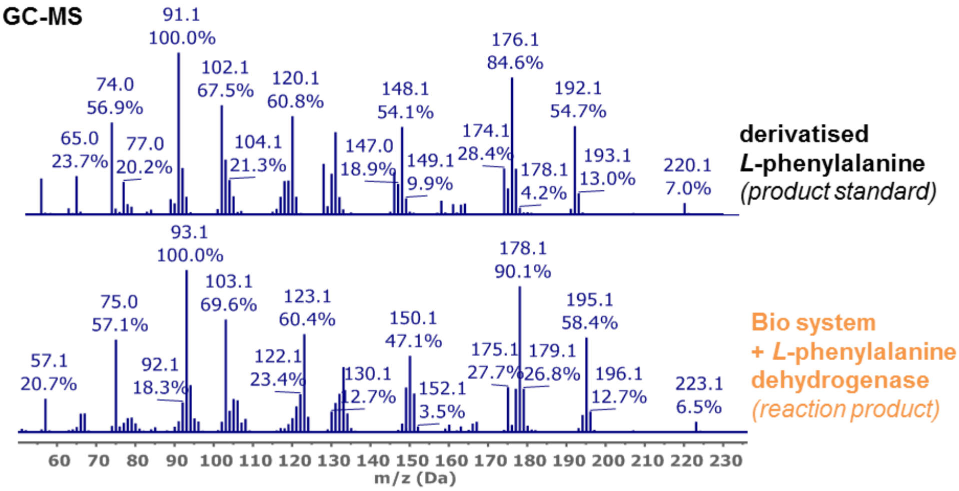

Supplementary Figure 30: Mass spectrum (from GC-MS) of the product of reductive amination and deuteration of phenylpyruvate (EI, 70 eV). The increase in molecular weight by +3.0 of the phenylalanine product (compared to an isotopically unenriched commercial standard) demonstrates the expected ^{2}H incorporation by the biocatalyst.

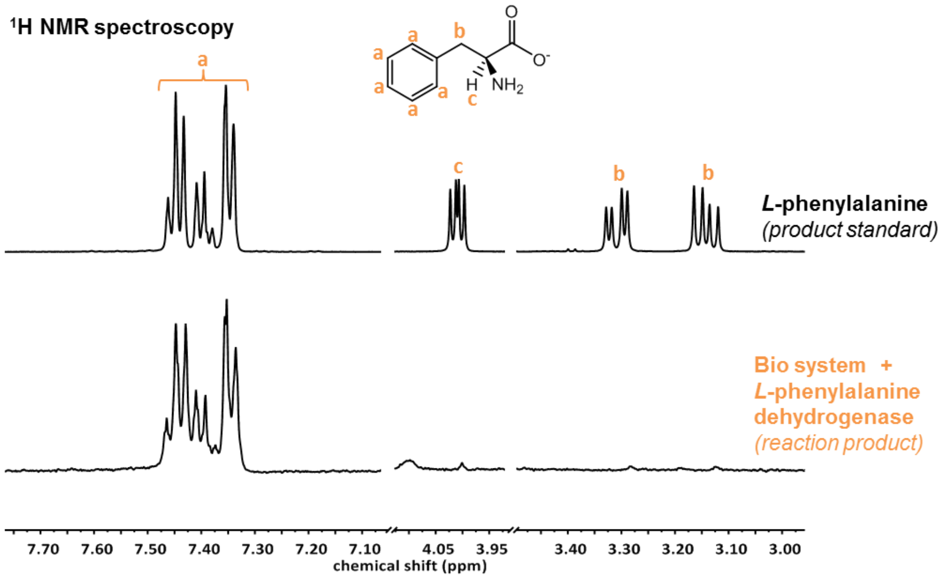

Supplementary Figure 31: ^{1}H NMR analysis of products of reductive amination and deuteration of phenylpyruvate (^2H_2O, p^{2}H 8.0, 500 MHz, 293 K). Comparison of the reaction product with a commercial standard of isotopically unenriched phenylalanine shows that high %^{2}H incorporation was achieved by the biocatalyst.

Reductive deuteration of carvones (5a, 5b)

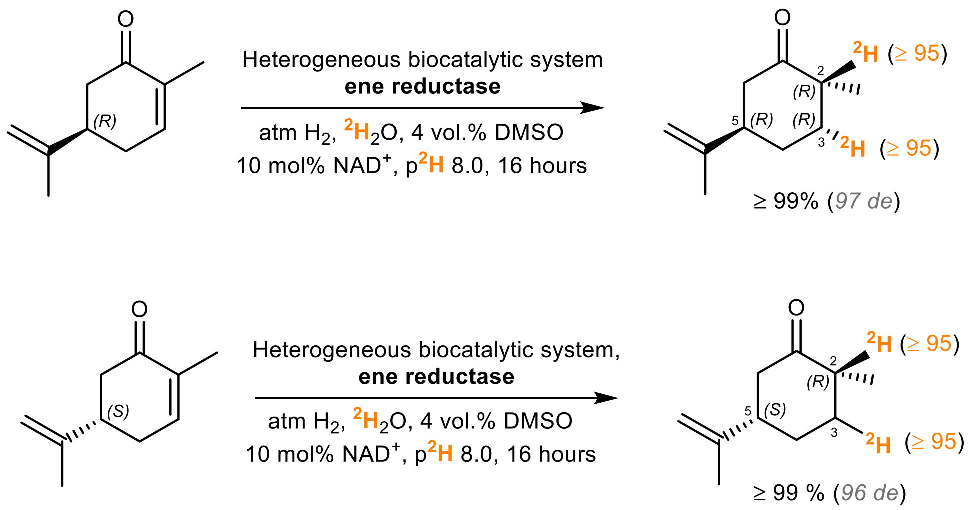

Supplementary Figure 32: Reductive deuteration of carvones. The H_2-driven biocatalytic system was used for the reductive deuteration of (5*R*)- and (5*S*)-carvone to the corresponding (2*R*,5*R*)- and (2*R*,5*S*)-[2,3-$^2H_2$]-dihydrocarvones (**5a**, **5b**). It was also possible to assign the 3-position of (2*R*,5*R*)-[2,3-$^2H_2$]-dihydrocarvone as (*R*) in reaction **5a**.

Reaction conditions: Reaction was conducted in 0.5 mL of ($^2H_5$)-Tris-^{2}HCl (100 mM, p^{2}H 8.0) containing 5 mM (5*R*)-carvone (Reaction **5a**) or (5*S*)-carvone (Reaction **5b**), 4 vol% [$^2H_6$]-DMSO, 0.5 mM NAD$^+$, and 500 μg of ene reductase (JM, ENE101). The mixture was presaturated with H_2 gas prior to addition of the heterogeneous bio-system at a loading of 400 μg. The reactions were shaken at 500 rpm under a steady flow of H_2 for 16 hours. Evaporation of the product dihydrocarvones proved to more problematic than in other cases so alternative reaction setups were trialled, including a pressure vessel (Büchi Tinyclave) sealed under 2 bar H_2 (50 mL headspace), and a reaction in a 1.5 mL glass GC-vial sealed under H_2 balloon pressure.

Whilst a commercial standard of mixed of (2*R*,5*R*)- and (2*S*,5*R*)-dihydrocarvone was available, the 5*S*-epimer was unavailable. Instead, this was synthesised by reduction of (5*S*)-carvone by Zn metal, according to the method of Leiva de Faria *et al.*[7] Here, Zn metal (625 mg, 9.6 mmol) and KOH (250 mg, 4.5 mmol) were combined in 3.5 mL of MeOH/H_2O (95/5 v/v) and the slurry bought to reflux under stirring. (5*S*)-carvone (500 mg, 3.3 mmol) was dissolved in 1 mL of MeOH/H_2O (95/5 v/v) and added dropwise to the Zn-solution over 6 hours. Following the reaction, the solution was filtered through a syringe filter, extracted into pentane (3 × 1 mL) and dried over Na_2SO_4. The solvent was removed *in vacuo* to give (5*S*)-dihydrocarvone (400 mg, 80%) as a mixture of *cis*-2,5 and *trans*-2,5 epimers in the ratio 83:17 (by GC).

In order to assist in the assignment of the ^{1}H NMR spectra of the deuterated samples, (2*R*,5*R*)- and (2*R*,5*S*)-dihydrocarvones of natural isotopic abundance were also prepared at high *de* from their parent (5*R*)- and (5*S*)-carvones by reaction with an ene reductase. Here, the relevant carvone (5 mM) was shaken with 500 μg of ene reductase (JM, ENE101) in 500 μL of non-deuterated Tris buffer (100 mM, pH 8.0) containing 2 vol% DMSO and 10 mM NADH for 6 hours at room temperature. ^2H_2O (100 μL) was added to the sample for field locking purposes, and the reaction mixture was analysed immediately by ^{1}H NMR spectroscopy.

<u>GC-FID:</u> Following the reactions, an aliquot of the solution was extracted with a 2 × volume of C^2HCl_3, and then centrifuged at 18,800 × g for 5 minutes before being transferred to glass vials for analysis by GC-FID in order to determine conversion. The results for reactions **5a** and **5b** are shown in Supplementary Figure 33 and Supplementary Figure 36, respectively. The GC-FID was carried out using the following parameters:

Column: DB-1701 (Agilent), 30 m length, 0.25 mm diameter, 0.25 μm film thickness
Carrier: He (CP grade), 0.5 mL minute (constant flow)
Inlet temperature: 230 ˚C
Injection gases: Splitless with split flow 60 mL min^{-1}, splitless time 0.8 min, purge 5 mL min^{-1}
Injection volume: 0.5 μL
Detection: FID
Detector temperature: 150 ˚C
Detection gases: H_2 (35 mL min^{-1}), air (350 mL min^{-1}), makeup N_2 (40 mL min^{-1})
Oven heating profile:

0 → 5 mins	Hold at 50˚C
5 → 25 mins	Ramp to 250 ˚C at 10 ˚C min^{-1}
25 → 35 mins	Hold at 250 ˚C for 10 min

<u>GC-MS:</u> Following extraction and analysis by GC-FID (above) the reaction solutions were analysed by GC-MS in order to determine the extent of isotopic labelling. The results for reactions **5a** and **5b** are shown in Supplementary Figure 34 and Supplementary Figure 37, respectively. The parameters used for the GC-MS analysis were as follows:

Column: DB-1701 (Agilent), 30 m length, 0.25 mm diameter, 0.25 μm (film thickness)
Carrier: He (CP grade), 100 kPa (constant pressure)
Inlet temperature: 250 ˚C
Injection gases Split (10:1) with split flow 12 mL min^{-1}, splitless time 0.7 min, purge 5 mL min^{-1}
Detection: Mass Spec, EI (70 eV), source temperature 230 ˚C. Scan range 50 – 650 amu with a scan rate of 5 Hz. Transfer line 300 ˚C.
Oven heating profile:

0 → 5 mins	Hold at 50˚C
5 → 16.3 mins	Ramp to 275 ˚C at 20 ˚C min^{-1}
16.3 → 18.8 mins	Hold at 275 ˚C for 2.5 min

<u>^{1}H NMR spectroscopy:</u> After removal of the catalyst, the unmodified reaction solutions were analysed by ^{1}H NMR spectroscopy at 500 MHz and 298 K, with the results for reactions **5a** and **5b** being shown in Supplementary Figure 35 and Supplementary Figure 38, respectively. In order to confirm assignments, ^{1}H-^{1}H correlation spectroscopy (COSY) was also conducted on the samples as required.

Analysis of Reaction 5a: Analysis by GC-FID confirmed the high conversion of substrate to product, and the high selectivity for the *trans*-2,5 diastereomer (Supplementary Figure 33). The slightly compromised diastereomeric excess (97%) is most likely a consequence of the racemisation of the dihydrocarvone product, which occurs slowly in aqueous solutions. Quantitative analysis revealed that there was a considerable loss of product from the reaction mixture when H_2 was flowed across the headspace, and so a mass balance was not achieved in this case. Sealing the reaction under 2 bar H_2 minimised product losses due to evaporation, but still resulted in recovery of only about 30 % of the theoretical quantity.

The site and extent of ^{2}H labelling was demonstrated by ^{1}H NMR spectroscopy (Supplementary Figure 35), the assignment of which correlated well with those of previous authors.[7] Changes to the spectrum following the deuteration reaction confirmed both the highly specific ^{2}H incorporation, and high diastereoselectivity that was achieved. Disappearance of the multiplet at δ = 2.61 ppm (labelled **b** in the diagram) and collapse of the coupling on the doublet at δ = 0.99 ppm with J = 6.4 Hz, were clear evidence of the ^{2}H installation on C2. Similarly, loss of the *pseudo*-quartet peak at δ = 1.41 ppm (labelled $\mathbf{c_b}$ in the diagram), with simultaneous loss of coupling on signals representing neighbouring protons ($\mathbf{d_a}$ and $\mathbf{d_b}$ at δ = 1.94 ppm and δ = 1.72 ppm, respectively), provided evidence of a ^{2}H nucleus at C3. The preservation of the peak at δ = 2.18 ppm (labelled $\mathbf{c_a}$ in the diagram), which collapses from a multiplet to a broad singlet, is evidence that a single ^{2}H nucleus is installed stereoselectively on C3.[8] As the ^{2}H on C3 has higher chiral priority than the ^{1}H, the product may be assigned as (2*R,*3*R*,5*R*)-(2,3-$^2H_2$)-dihydrocarvone.[9] Hence, the two ^{2}H nuclei are added in an *anti*-relationship across C2 and C3, which is in accordance with previous mechanistic studies of similar ene reductase enzymes.[8,9]

Finally, GC-MS analysis of the reaction solution further confirms the addition of two ^{2}H nuclei across the molecule (Supplementary Figure 34), with the molecular ion being seen to shift from m/z = 152.1 for [$C_{10}H_{16}O^{+\bullet}$], to 154.1 for [$^2H_2$-$C_{10}H_{14}O^{+\bullet}$].

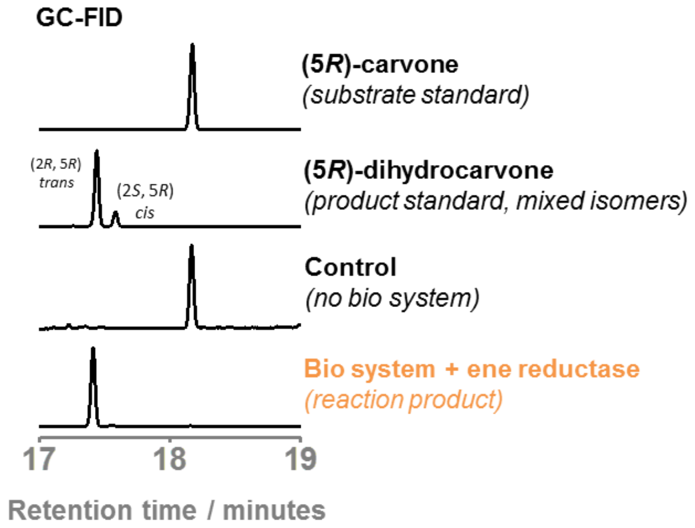

Supplementary Figure 33: GC-FID analysis of the reductive deuteration of (5*R*)-carvone. The dihydrocarvone product standard contains a mixture of *cis* (minor) and *trans* (major) isomers, and the GC-FID shows that the biocatalyst system only produces the *trans*-form.

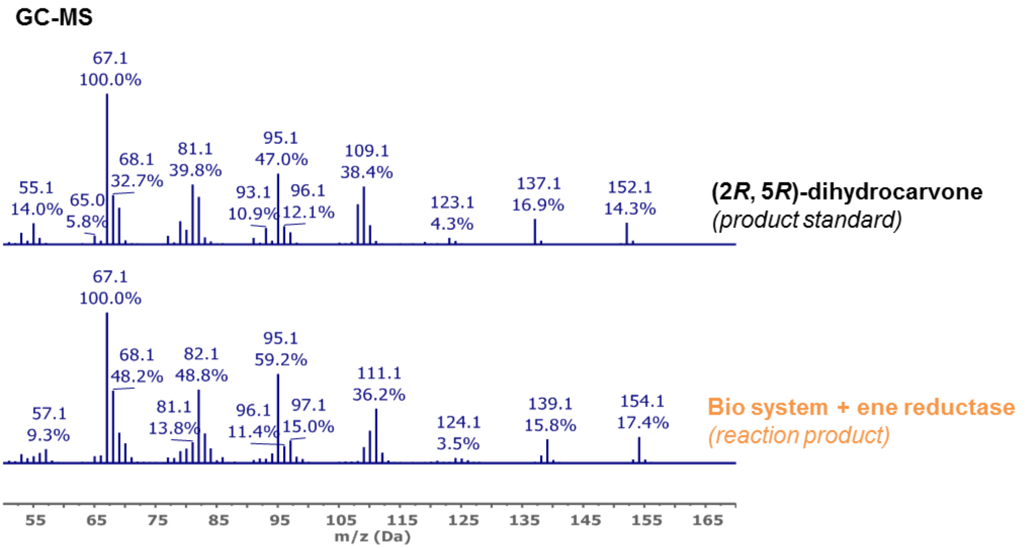

Supplementary Figure 34: Mass spectrum (from GC-MS) of the product of reductive deuteration of (5*R*)-carvone (EI, 70 eV). The increase in molecular weight by +2.0 of the dihydrocarvone product (compared to an isotopically unenriched commercial standard) demonstrates the expected ^{2}H incorporation by the biocatalyst.

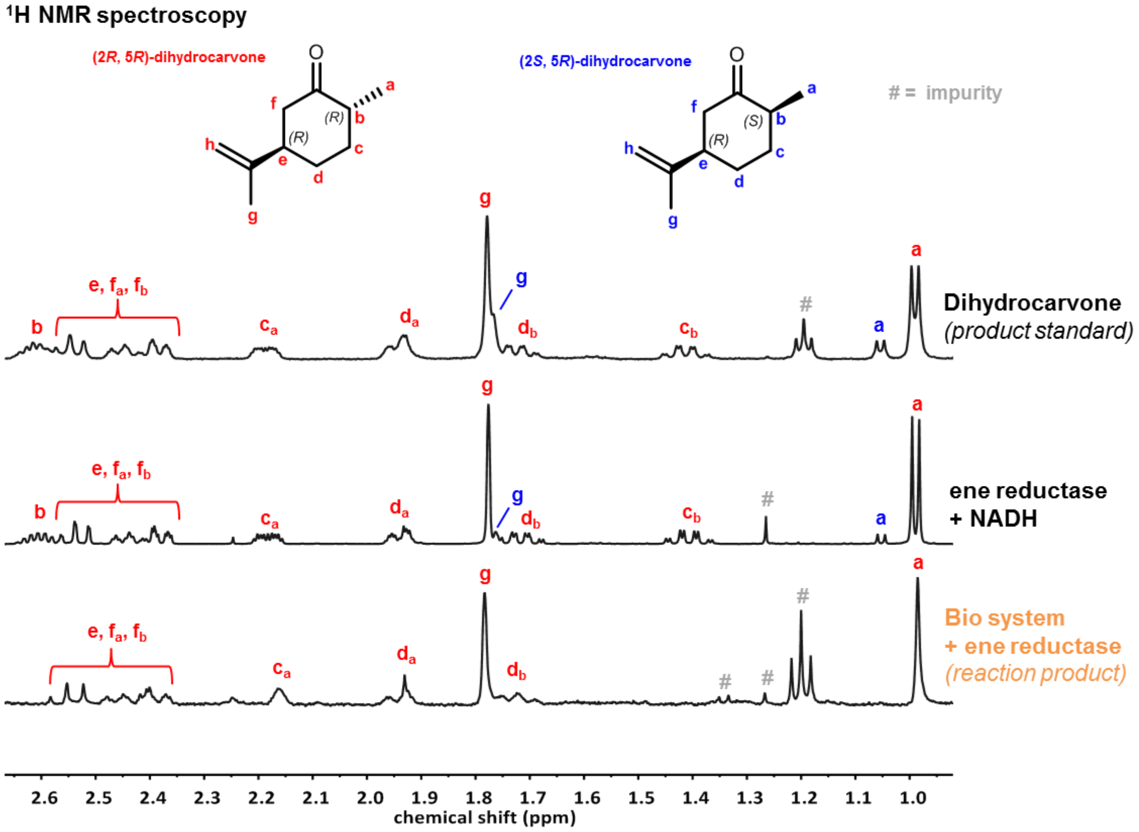

Supplementary Figure 35: ^{1}H NMR analysis of products of the reductive deuteration of (5*R*)-carvone (^2H_2O, p^{2}H 8.0, 500 MHz, 293 K). The reaction mixture from the heterogeneous biocatalyst was compared to two unlabeled standards of (5*R*)-dihydrocarvone (a commercial sample and a sample prepared from the reaction of (5*R*)-carvone with ene reductase and NADH). The ^{1}H NMR spectra demonstrate the high selectivity of the biocatalytic system for (2*R*,5*R*)-dihydrocarvone, and the high %^{2}H incorporation on C2 and C3.

Analysis of Reaction 5b: Analysis by GC-FID (Supplementary Figure 36) yielded results that were similar to those for Reaction **5a** described above. Again, conversion from (5*S*)-carvone to (2*R*,5*S*)-dihydrocarvone was found to be virtually quantitative, though evaporation meant that recovery of the product was quite poor. Conducting the reaction in a sealed vial under a balloon pressure of H_2 (as opposed to a dynamic flow of H_2 across the headspace) helped to improve the product yield to around 30% of the theoretical quantity. Unlike reaction **5a**, the *cis*-(2*R*,5*S*)-isomer if formed, which represents the least stable of the two possible diastereomeric products (with racemisation giving rise to a slightly diminished *de* of 96%).

^{1}H NMR spectroscopic analysis of the reaction solution provided insight into the extent and location of ^{2}H incorporation into the product (Supplementary Figure 38). Here, the dihydrocarvone standard prepared by a conventional Zn reduction was not found to be useful, as it contained mostly the thermodynamically favoured *trans*-(2*R*,5*R*)-dihydrocarvone isomer. Instead, a standard prepared by supplying the ene reductase with (5*S*)-carvone and a super-stoichiometric quantity of NADH in non-deuterated buffer was found to generate a suitable sample of (2*R*,5*S*)-dihydrocarvone of natural isotopic abundance for comparison. The ^{1}H NMR spectrum of the deuterated product is less well resolved than in Reaction **5a**, but the disappearance of signals at δ = 2.56 ppm and δ = 1.58 ppm (labelled **b** and $\mathbf{c_b}$ respectively) along with the collapse of coupling on the doublet at δ = 1.05 ppm can be clearly seen. The convoluted peak at δ = 1.93 ppm retains a relative integration of 3H on going from the standard to the deuterated reaction, confirming that the area of signal $\mathbf{c_a}$ is not diminished. All of these factors provide evidence for two ^{2}H nuclei being installed across the double bond, although the likely *anti*-arrangement cannot be confirmed unambiguously.

Finally, GC-MS analysis of the reaction solution further indicates the addition of two ^{2}H nuclei across the molecule (Supplementary Figure 37), with the molecular ion being seen to shift from m/z = 152.1 for [$C_{10}H_{16}O^{+\bullet}$], to 154.1 for [$^2H_2$-$C_{10}H_{14}O^{+\bullet}$].

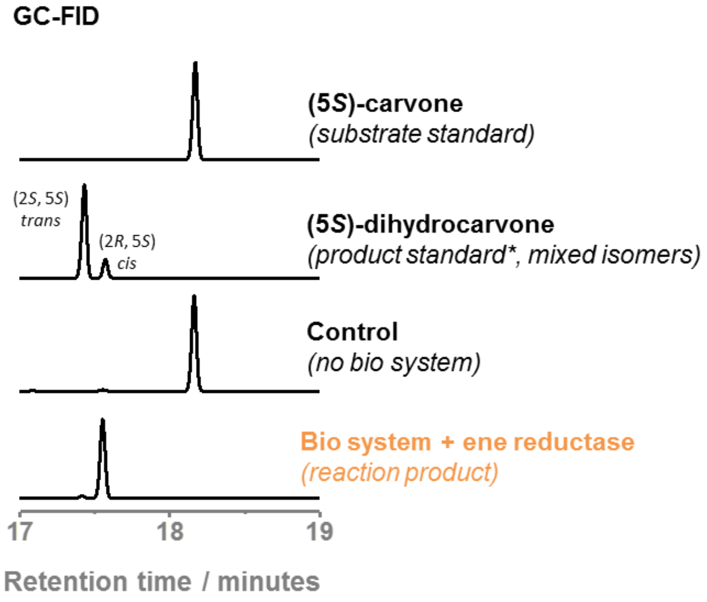

Supplementary Figure 36: GC-FID analysis of the reductive deuteration of (5*S*)-carvone. The dihydrocarvone product standard contains a mixture of *cis* (minor) and *trans* (major) isomers, and the GC-FID shows that the biocatalyst has very high selectivity for the *cis*-form.

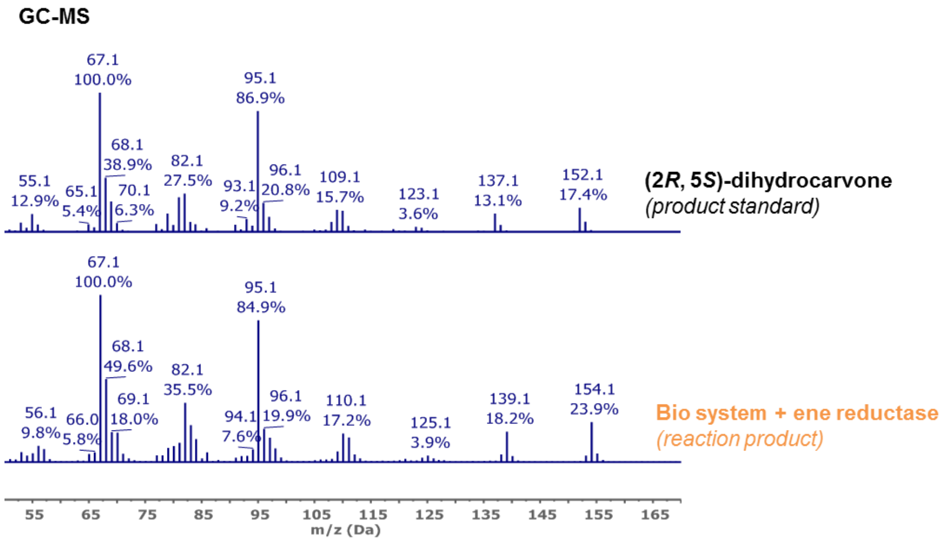

Supplementary Figure 37: Mass spectrum (from GC-MS) of the product of reductive deuteration of (5*S*)-carvone (EI, 70 eV). The increase in molecular weight by +2.0 of the dihydrocarvone product (compared to an isotopically unenriched commercial standard) demonstrates the expected ^{2}H incorporation by the biocatalyst.

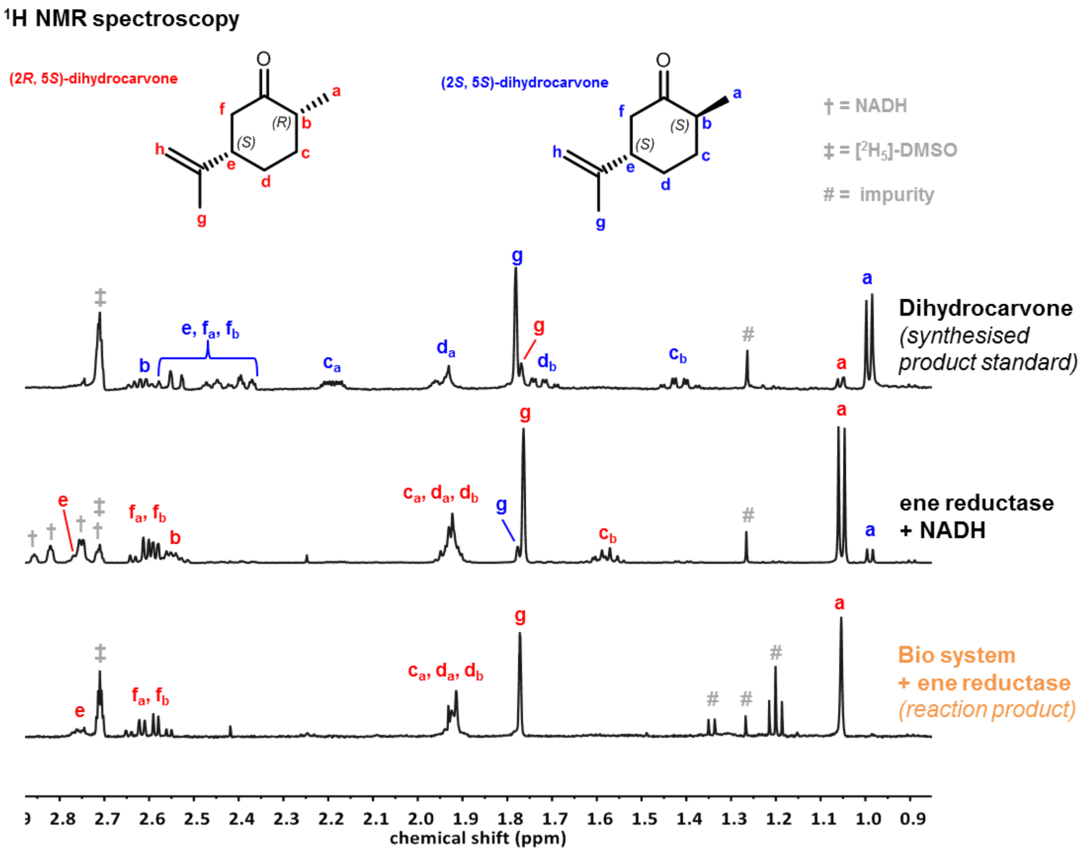

Supplementary Figure 38: ^{1}H NMR analysis of products of the reductive deuteration of (5*S*)-carvone (^2H_2O, p^{2}H 8.0, 500 MHz, 293 K). The reaction mixture from the heterogeneous biocatalyst was compared to two unlabeled standards of (5*S*)-dihydrocarvone (a sample prepared from the reaction of (5*R*)-carvone with zinc and another from the reaction with ene reductase and NADH). The ^{1}H NMR spectra demonstrate the high selectivity of the biocatalytic system for (2*R*,5*S*)-dihydrocarvone, and the high %^{2}H incorporation on C2 and C3.

Reductive deuteration of cinnamaldehyde (6a, 6b, 6c)

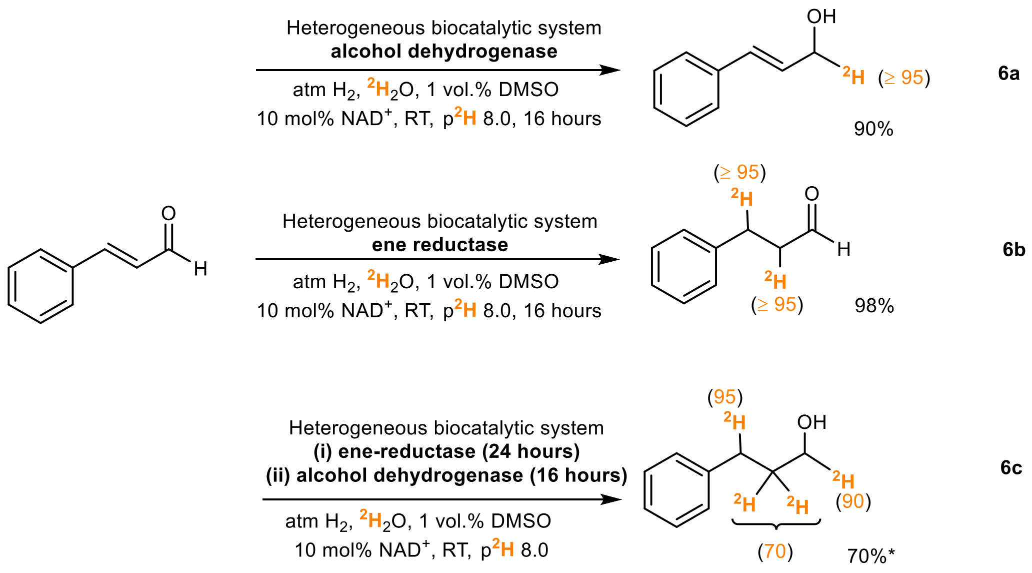

Supplementary Figure 39: Chemoselective reductive deuteration of cinnamaldehyde. The H_2-driven biocatalytic system was used to produce, singly, doubly and multiply deuterated products (**6a**, **6b**, **6c**). *Although ≥ 98% of the substrate was consumed in reaction **6c**, a side reaction was observed that accounted for around 30 % of the total product.

__Reaction conditions:__ Reactions were conducted in 0.5 mL of ($^2H_{11}$)-Tris-^{2}HCl (100 mM, p^{2}H 8.0) containing 5 mM cinnamaldehyde, 2 vol% DMSO, 0.5 mM NAD$^+$, and 500 μg of (**6a**) alcohol dehydrogenase (JM, ADH 105), and (**6b**, **6c**) ene reductase (JM, ENE101). The mixture was presaturated with H_2 gas prior to addition of the heterogeneous biocatalytic system at a loading of 400 μg. The reactions were shaken at 500 rpm under a steady flow of H_2 for 16 hours. In the case of reaction **6c**, the initial step was allowed to proceed for 24 hours before addition of a further 200 μg of the heterogeneous biocatalytic system and 500 μg of alcohol dehydrogenase (JM, ADH 105).

__GC-FID:__ Following the reactions, an aliquot of the solution was extracted with a 2 × volume of C^2HCl_3, and then centrifuged at 18,800 × g for 5 minutes before being transferred to glass vials for analysis by GC-FID to determine conversion. The results for reactions **6a**, **6b**, and **6c** are shown in Supplementary Figure 40, Supplementary Figure 43, and Supplementary Figure 46, respectively. The parameters used for GC-FID analysis were as follows:

Column: DB-1701 (Agilent), 30 m length, 0.25 mm diameter, 0.25 μm film thickness
Carrier: He (CP grade), 0.5 mL minute (constant flow)
Inlet temperature: 230 ˚C
Injection gases: Splitless with split flow 60 mL min^{-1}, splitless time 0.8 min, purge 5 mL min^{-1}
Injection volume: 0.5 μL
Detection: FID
Detector temperature: 150 ˚C
Detection gases: H_2 (35 mL min^{-1}), air (350 mL min^{-1}), makeup N_2 (40 mL min^{-1})
Oven heating profile:

0 → 5 mins	Hold at 50˚C
5 → 25 mins	Ramp to 250 ˚C at 10 ˚C min^{-1}
25 → 35 mins	Hold at 250 ˚C for 10 min

^{1}H NMR spectroscopy: After removal of the catalyst, the unmodified reaction solutions were analysed by ^{1}H NMR spectroscopy at 500 MHz and 298 K, with the results for reactions **6a**, **6b**, and **6c** being shown in Supplementary Figure 41, Supplementary Figure 44, and Supplementary Figure 47, respectively. In the case of reactions **6a** and **6c**, it was necessary to acquire additional spectra under non-aqueous conditions by extracting the product into an equal volume of $C^{2}HCl_3$ and re-analysing it.

GC-MS: Following extraction and analysis by GC-FID (above) the reaction solutions were analysed by GC-MS in order to determine the extent of isotopic labelling. The results for reactions **6a**, **6b**, and **6c** are shown in Supplementary Figure 42, Supplementary Figure 45, and Supplementary Figure 48, respectively. The parameters used for GC-MS analysis were as follows:

Column: DB-1701 (Agilent), 30 m length, 0.25 mm diameter, 0.25 μm (film thickness)
Carrier: He (CP grade), 100 kPa (constant pressure)
Inlet temperature: 250 ˚C
Injection gases Split (25:1) with split flow 30 mL min^{-1}, splitless time 0.7 min, purge 5 mL min^{-1}
Detection: Mass Spec, EI (70 eV), source temperature 230 ˚C. Scan range 50 – 650 amu with a scan rate of 5 Hz. Transfer line 300 ˚C
Oven heating profile:

0 → 5 mins	Hold at 50˚C
5 → 16.3 mins	Ramp to 275 ˚C at 20 ˚C min^{-1}
16.3 → 18.8 mins	Hold at 275 ˚C for 2.5 min

Analysis of Reaction 6a: Analysis by GC-FID (Supplementary Figure 40) shows a broad peak for the cinnamyl alcohol product (RT = 19.9 min) and one for unreacted cinnamaldehyde (RT = 19.4 min), with no additional products being detected (including hydrocinnamalehyde and 3-phenyl-1-propanol, which are common side products in the hydrogenation of cinnamaldehyde).[10] Integration of the peak areas compared to a set of standard solutions indicated a conversion of 90 %, which could be confirmed by comparison of clearly resolved peaks in the ^{1}H NMR spectrum of the crude reaction mixture.

The site and extent of ^{2}H labelling in the cinnamyl alcohol product was determined through ^{1}H NMR spectroscopy (Supplementary Figure 41). The intensity of the doublet at δ = 4.30 ppm (J = 5.5 Hz) in the unlabelled cinnamyl alcohol standard (corresponding to the α-protons) halves in intensity in the spectrum of the biocatalytic reaction product. Overlapping peaks from the ribose ring of the cofactor make a precise integration of the peak from the α-proton difficult, though extracting the product into $C^{2}HCl_3$ and re-acquiring the spectrum enabled the measurement to be conducted unambiguously. Hence, it was confirmed that the α-site had been labelled with a single ^{2}H nucleus. Accordingly, the peak corresponding to the neighbouring C2 proton is also found to change from a doublet of triplets in the unlabelled standard (δ = 6.47 ppm, dt, J = 16.1 Hz, J = 5.8 Hz), to a doublet of doublets in the deuterated product (δ = 6.47 ppm, dd, J = 16.1 Hz, J = 5.8 Hz).

Unfortunately, mass spectroscopic analysis could not be used to unambiguously evaluate the extent of %^{2}H labelling, as such compounds are known to re-arrange upon ionization, causing large differences in the fragmentation pattern of different isotopomers.[11] However, the spectra were still acquired, and are shown in Supplementary Figure 42 for completeness. Here, the molecular ion peak in the commercial cinnamyl alcohol standard ([$C_9H_{10}O^{+\bullet}$], m/z 134.1) is seen to increase by +1.0 for the product of the biocatalytic hydrogenation, consistent with the addition of a ^{2}H nucleus ([$^{2}H_1$-$C_9H_9O^{+\bullet}$], m/z 135.1).

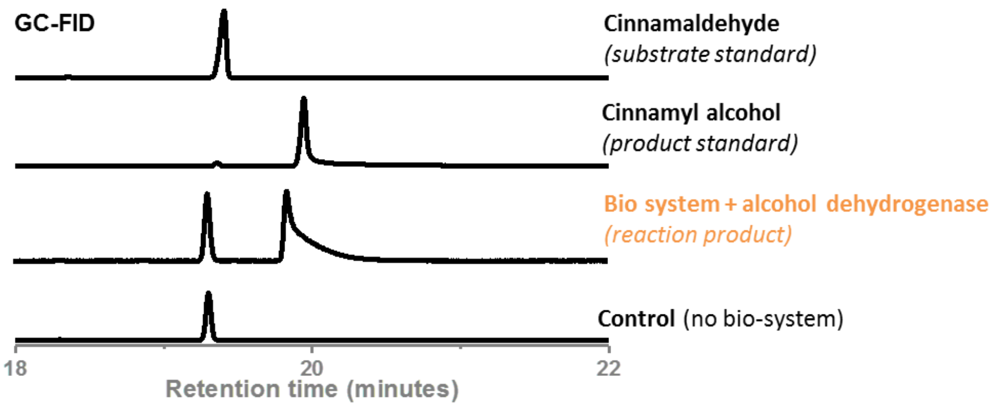

Supplementary Figure 40: GC-FID analysis of products of reductive deuteration of cinnamaldehyde by the heterogeneous biocatalytic system coupled to an alcohol dehydrogenase. Comparison of the reaction mixture to a sample of commercial cinnamyl alcohol demonstrates that the reduction of the carbonyl group took place as expected, with no side-product formation.

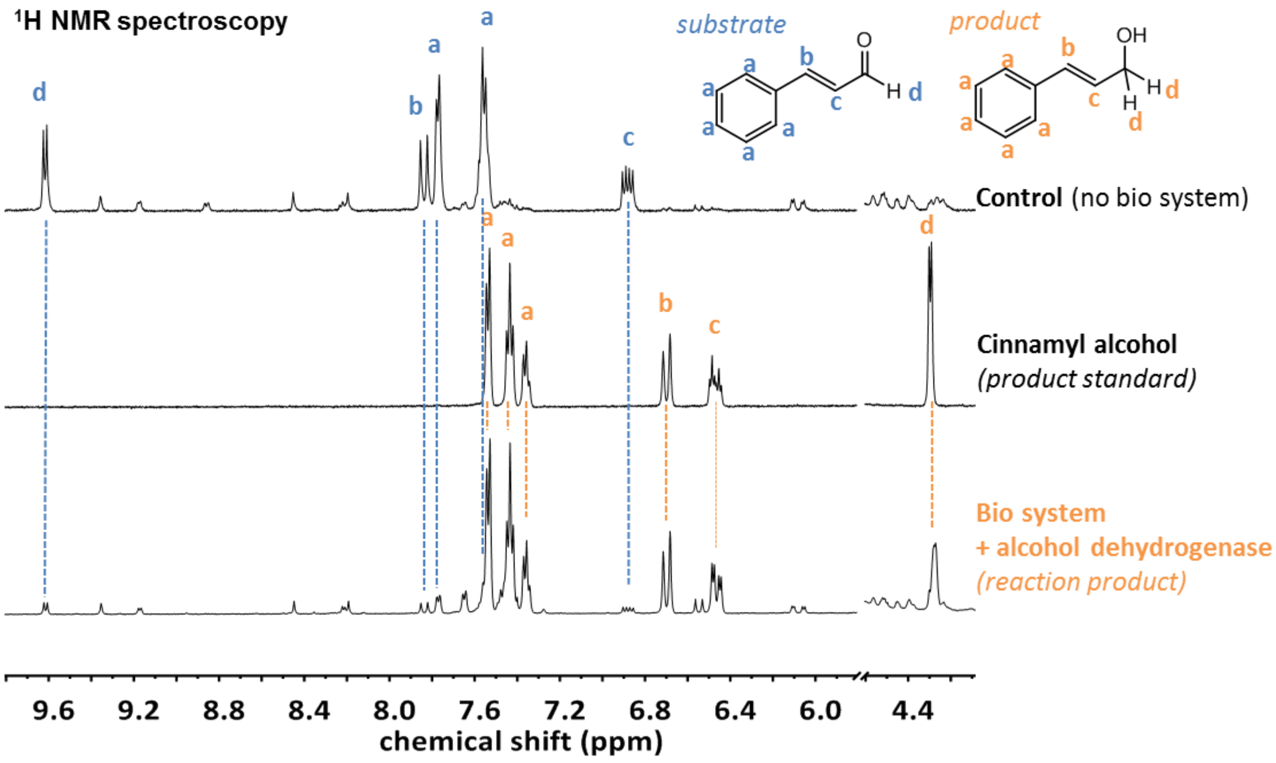

Supplementary Figure 41: ^1H NMR analysis of products of reductive deuteration of cinnamaldehyde by the heterogeneous biocatalytic system coupled to an alcohol dehydrogenase. (^2H_2O, p^{2}H 8.0, 500 MHz, 293 K). Comparison of the reaction products with a commercial standard of isotopically unenriched cinnamyl alcohol shows that high %^{2}H incorporation was achieved by the biocatalytic system.

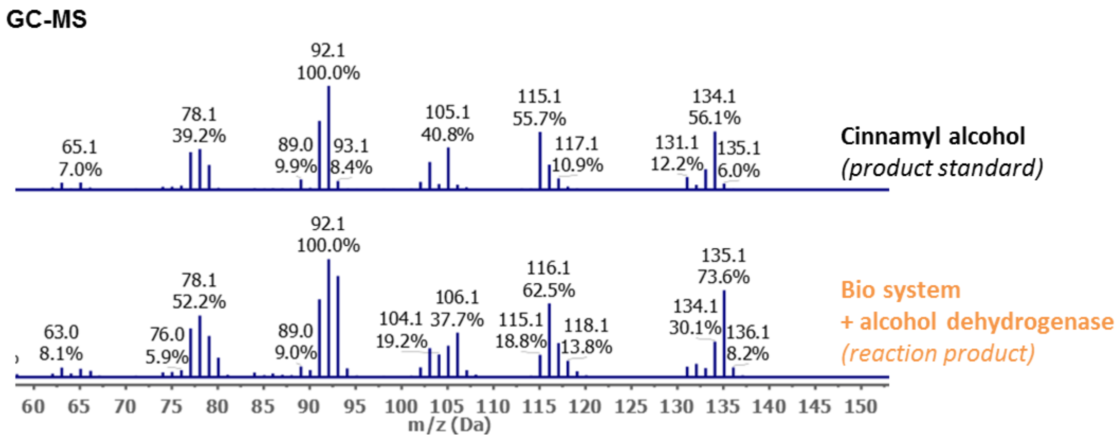

Supplementary Figure 42: Mass spectrum (from GC-MS) of the product of reductive deuteration of cinnamaldehyde by the heterogeneous biocatalytic system coupled to an alcohol dehydrogenase (EI, 70 eV). The increase in molecular weight by +1.0 of the cinnamyl alcohol product (compared to an isotopically unenriched commercial standard) demonstrates the expected ^{2}H incorporation by the biocatalyst.

Analysis of Reaction 6b: The near quantitative conversion of cinnamaldehyde to hydrocinnamaldehyde could was demonstrated by GC-FID analysis (Supplementary Figure 43). Here, the cinnamaldehyde substrate (RT = 19.4 min) was absent from the reaction solution, which showed a peak only for hydrocinnamaldehyde (RT = 17.5 min), without the formation of other possible side products (namely cinnamyl alcohol and/or 3-phenyl-1-propanol). This result was further confirmed by ^{1}H NMR analysis of the crude reaction mixture (see Supplementary Figure 44).

The site and extent of ^{2}H labelling in the hydrocinnamaldehyde product could also be determined through ^{1}H NMR spectroscopy, (see Supplementary Figure 44), although the interpretation is complicated by the speciation of the molecule in Tris-buffer. As such, each of the aliphatic proton environments on the molecule (denoted **a**, **b**, and **c** in Supplementary Figure 44) give rise to three separate peaks in the corresponding ^{1}H spectrum, which must be summed together in order to determine the extent of ^{2}H incorporation at each site. Accordingly, signals corresponding to sites **a** and **b** lose half of their intensity when the molecule is prepared by biocatalytic deuteration, whilst proton **c** (and the aromatic protons) remains unchanged. The extent of the collapse in coupling across all of the peaks is also commensurate with the addition of single deuterons at positions **a** and **b**.

Unfortunately, mass spectroscopic analysis could not be used unambiguously to evaluate the extent of %^{2}H labelling as such compounds are known to re-arrange upon ionization, thereby causing large differences in the fragmentation pattern of different isotopomers.[11] However, the spectra were still acquired, and are shown in Supplementary Figure 45 for completeness. Here, the molecular ion peak in the commercial standard of hydrocinnamaldehyde ([$C_9H_{10}O^{+\bullet}$], m/z 134.1) is seen to increase by +2.0 for the product of the biocatalytic hydrogenation, consistent with the addition of two ^{2}H nuclei ([$^{2}H_2$-$C_9H_8O^{+\bullet}$], m/z 136.1).

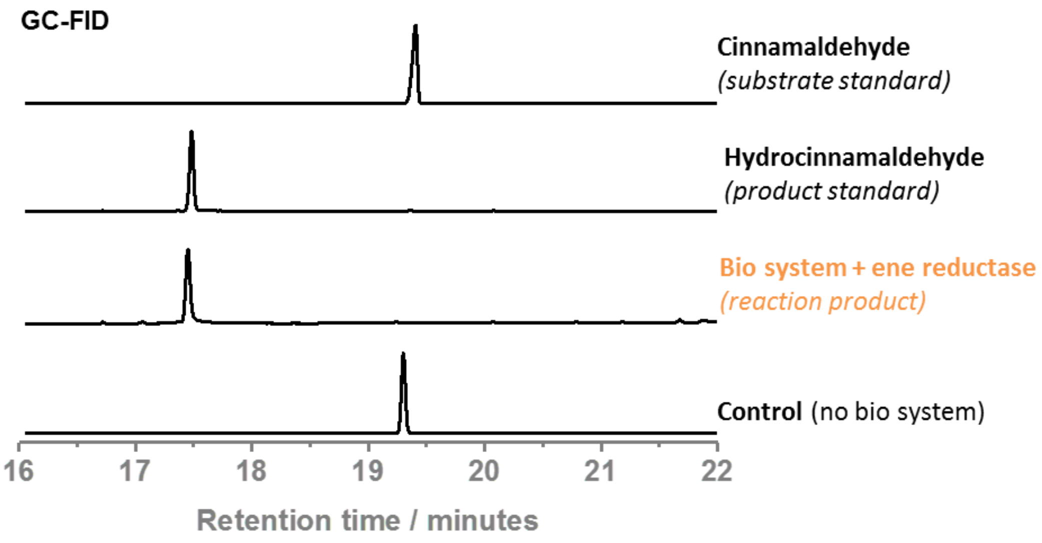

Supplementary Figure 43: GC-FID analysis of products of reductive deuteration of cinnamaldehyde by the heterogeneous biocatalytic system coupled to an ene reductase. Comparison of the reaction mixture to a sample of commercial hydrocinnamaldehyde demonstrates that the reduction of the ene group took place as expected, with no side-product formation.

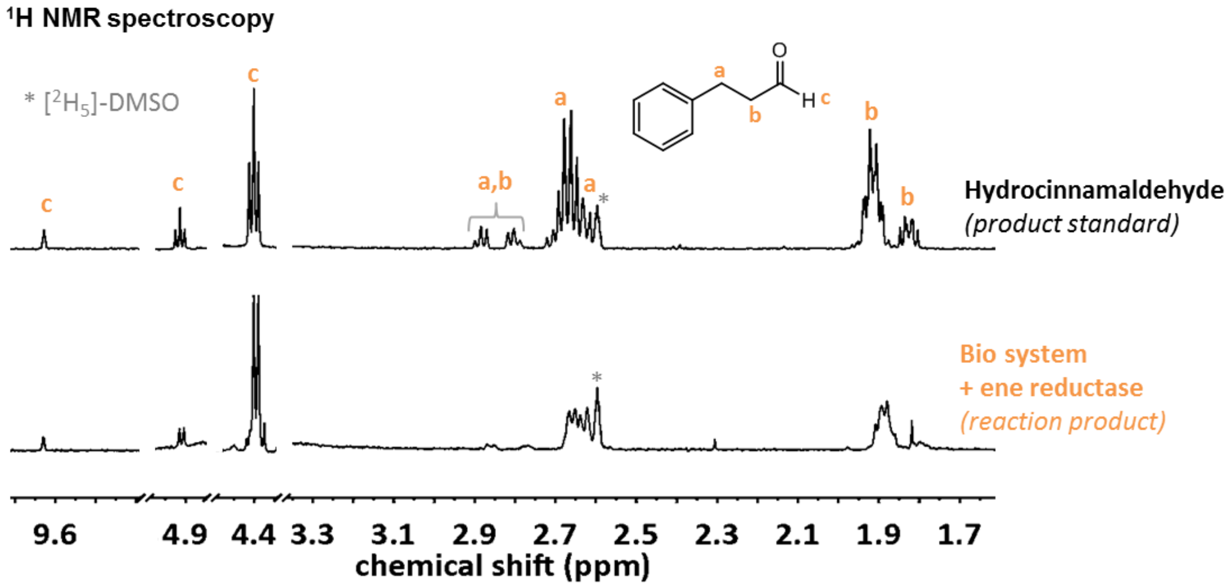

Supplementary Figure 44: ^1H NMR analysis of products of reductive deuteration of cinnamaldehyde by the heterogeneous biocatalytic system coupled to an ene reductase (^2H_2O, p^2H 8.0, 500 MHz, 293 K). Comparison of the reaction products with a commercial standard of isotopically unenriched hydrocinnamaldehyde shows that high %^2H incorporation was achieved by the biocatalytic system.

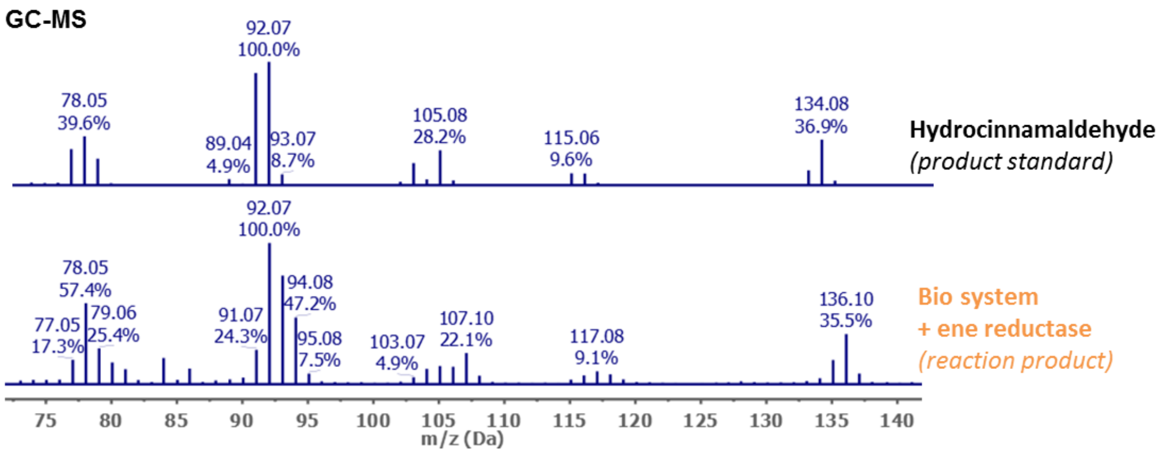

Supplementary Figure 45: Mass spectrum (from GC-MS) of the product of reductive deuteration of cinnamaldehyde by the heterogeneous biocatalytic system coupled to an ene reductase (EI, 70 eV). The increase in molecular weight by +2.0 of the hydrocinnamaldehyde product (compared to an isotopically unenriched commercial standard) demonstrates the expected ^{2}H incorporation by the biocatalyst.

<u>**Analysis of Reaction 6c:**</u> The conversion of cinnamaldehyde to hydrocinnamaldehyde by the action of the heterogeneous biocatalytic system and an ene reductase was shown to proceed quantitatively in Reaction **6b**. The second half of Reaction **6c**, in which the hydrocinnamaldehyde was reduced further to 3-phenyl-1-propanol by the introduction of an alcohol dehydrogenase, was shown to proceed to a similarly high degree by GC-FID analysis (see Supplementary Figure 46). Here, signals corresponding to the substrate (RT = 19.4 min) and intermediate (RT = 17.5 min) compounds were replaced in the chromatogram of the reaction mixture by the product peak (RT = 18.7 min), indicating a substrate conversion of ≥ 98%. Mass balance was not achieved however, with around 30 % of the material having been lost to evaporation over the course of the reaction. Further analysis by ^{1}H NMR spectroscopy (Supplementary Figure 47(a)) revealed that, in addition to the desired 3-phenyl-1-propanol, a side-product was formed that was not detected by GC-FID analysis. This product was determined to be [$^2H_2$]-phenylpropanoic acid, and forms from the disproportionation of [$^2H_2$]-hydrocinnamaldehyde, which can be catalyzed by some alcohol dehydrogenase enzymes.[12,13] The alcohol and acid products were formed in the approximate molar ratio 70:30, and were easily separated by extraction of the former compound into organic solvent.

The site and extent of ^{2}H labelling in the 3-phenyl-1-propanol product was determined through ^{1}H NMR spectroscopy, following extraction into C^2HCl_3, see Supplementary Figure 47(b). Here the intensity of signals corresponding to protons in position **b** (δ = 2.72 ppm) and **d** (δ = 3.68 ppm) are reduced by 45 - 50% following the deuteration reaction (compared to a standard sample of natural isotopic abundance), consistent with the addition of a single deuteron at each of these sites. The signal for the proton at position **c** (δ = 1.90 ppm) was found to decrease by 70% however, indicating that 1.4 ^{2}H units had been installed at this site. The additional ^{2}H incorporation in this latter case most likely occurred through tautomeric solvent exchange following the reduction of the C=C double bond. Upon deuteration, the clear splitting patterns for peaks **b** (a *pseudo*-triplet), **c** (a *pseudo*-quintet), and **d** (a *pseudo*-quartet), are replaced by broad, poorly defined signals, consistent with the influence of much weaker proton-deuteron coupling.

Mass spectroscopy was also used to study the isotopic substitution pattern along the 3-phenyl-1-propanol product, see Supplementary Figure 48. Here the molecular ion peak was found to be diagnostic, with the m/z 136.1 representing the unfragmented molecule.[14] Accordingly, peaks at m/z 139.1 and 140.1 in the analysis of the reaction mixture could be attributed to the inclusion of three or four ^2H nuclei, respectively, consistent with the various levels of labelling on C2 described above.

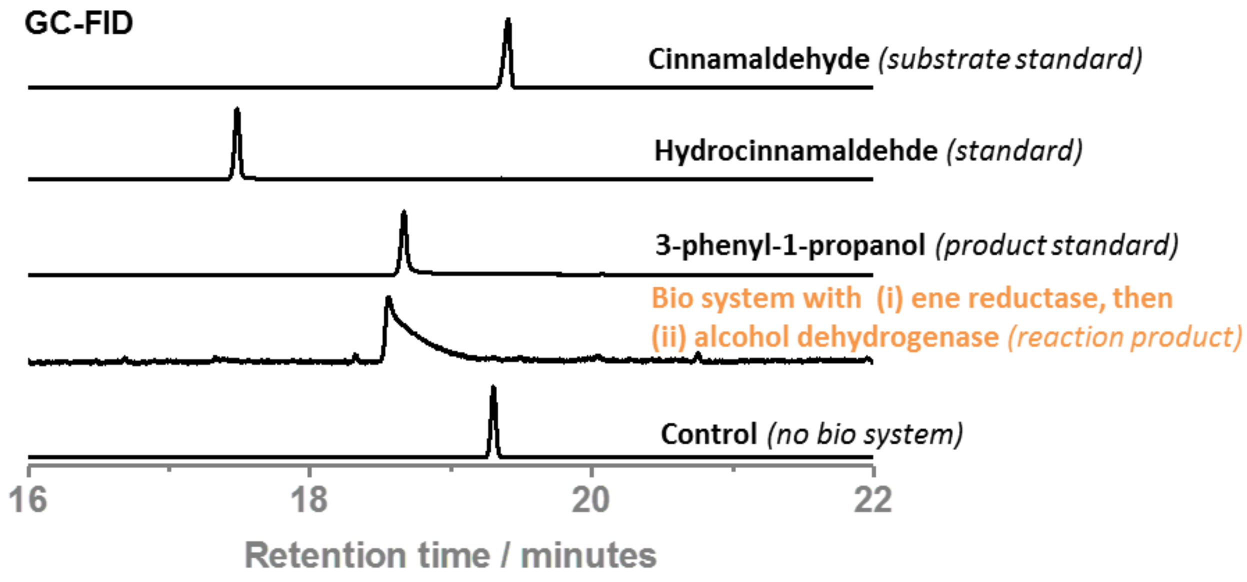

Supplementary Figure 46: GC-FID analysis of products of reductive deuteration of cinnamaldehyde by the heterogeneous biocatalytic system, an ene reductase, and an alcohol dehydrogenase. Comparison of the reaction mixture to a sample of commercial 3-phenyl-1-propanol demonstrates that the reduction of the ene and carbonyl groups took place as expected.

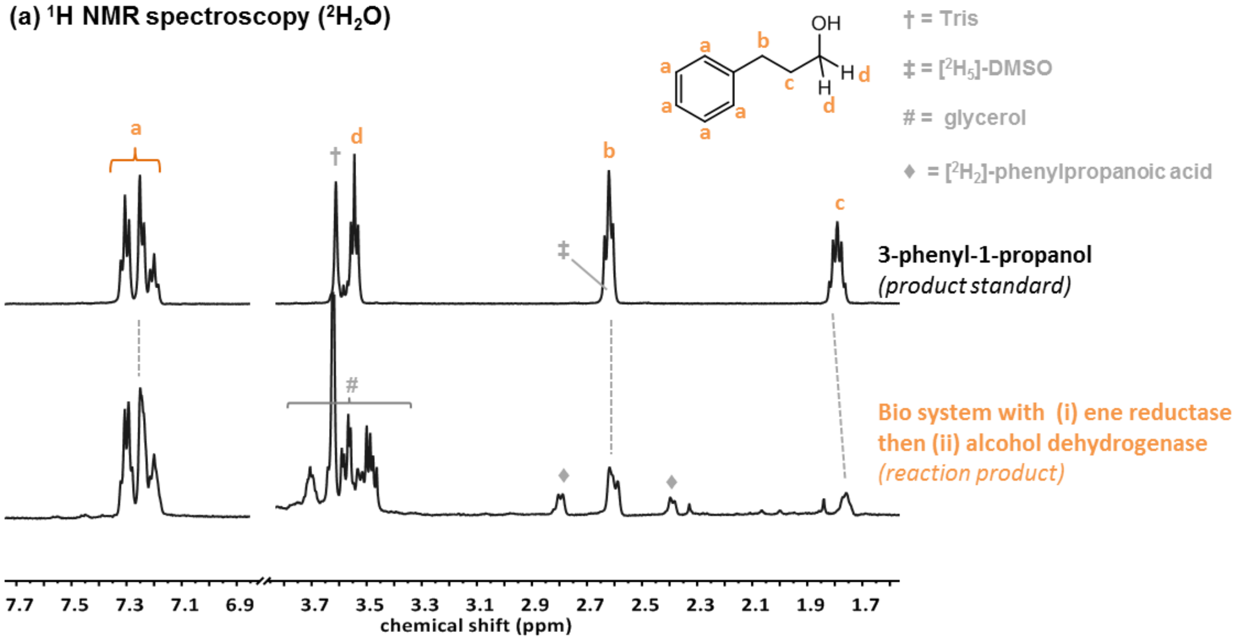

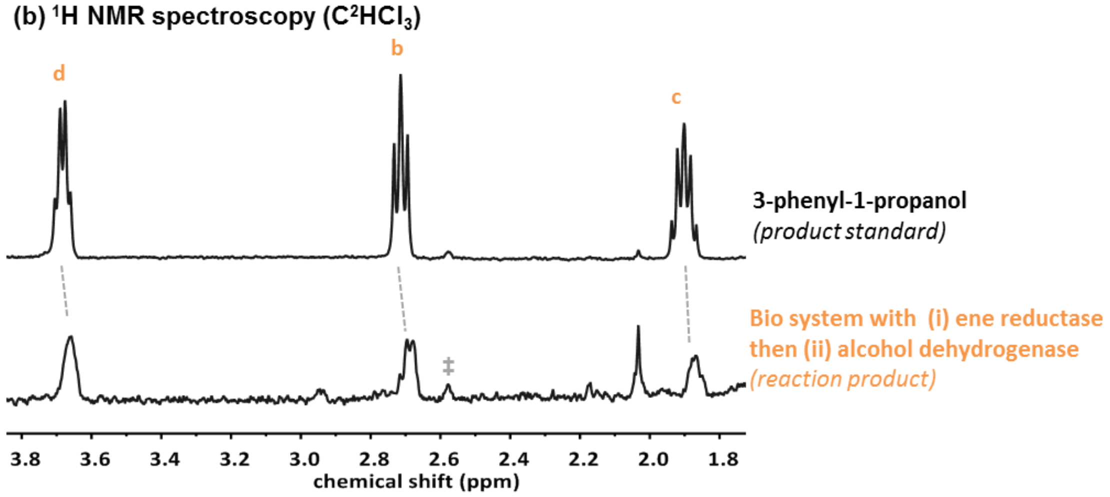

Supplementary Figure 47: ^{1}H NMR analysis of products of reductive deuteration of cinnamaldehyde by the heterogeneous biocatalytic system, an ene reductase, and an alcohol dehydrogenase (500 MHz, 293 K). **(a)** Product standard and reaction mixture in ^2H_2O (p^{2}H 8.0), **(b)** Product standard and reaction mixture extracted into $C^2H_2Cl_3$. Comparison of the reaction products with a commercial standard of isotopically unenriched 3-phenyl-1-propanol shows that high %^{2}H incorporation was achieved by the biocatalytic system.

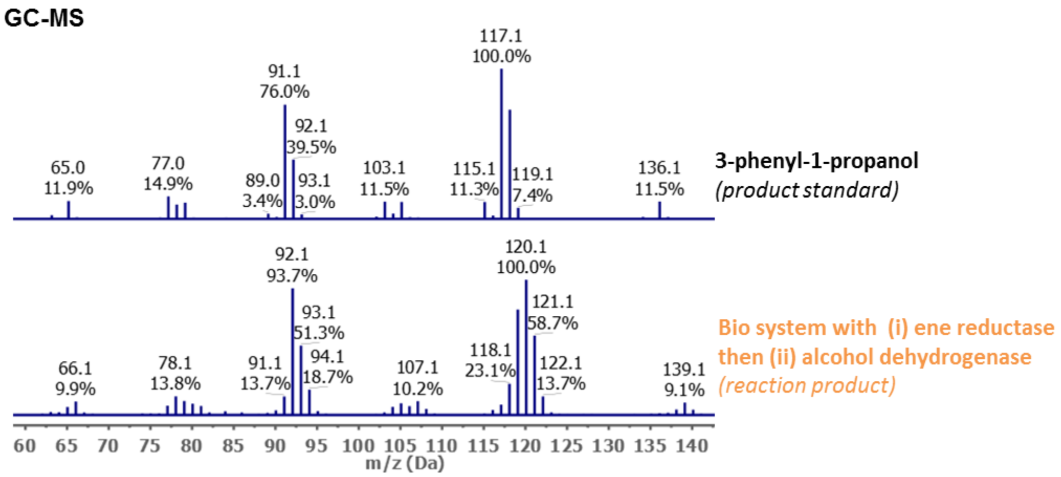

Supplementary Figure 48: Mass spectrum (from GC-MS) of the product of reductive deuteration of cinnamaldehyde by the heterogeneous biocatalytic system, an ene reductase, and an alcohol dehydrogenase (EI, 70 eV). The increase in molecular weight of the 3-phenyl-1-propanol product (compared to an isotopically unenriched commercial standard) demonstrates the multiple deuteration achieved by the biocatalyst system.

Synthesis of (3*R*)-[2,2,3-$^2H_3$]-quinuclidinol

Synthetic procedure: (3*R*)-[2,2,3-$^2H_3$]-Quinuclidinol was prepared according to the scheme outlined in Supplementary Figure 49, and described below:

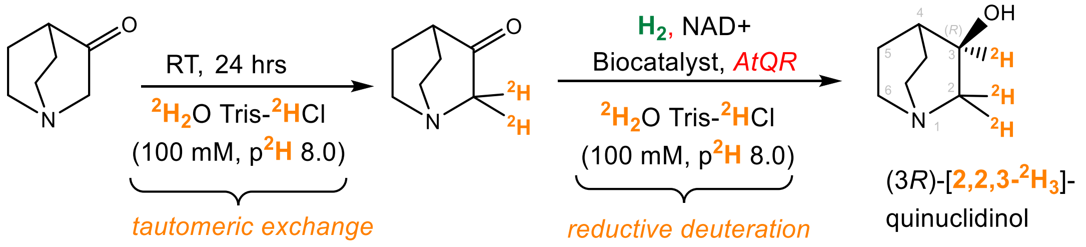

Supplementary Figure 49: Conversion of 3-quinuclidinone to (3*R*)-[2,2,3-$^2H_3$]-quinuclidinol. Two deuterium atoms were added to the molecule by tautomeric exchange with the ^2H_2O solvent. The asymmetric deuterium centre was created by using the H_2-driven heterogeneous biocatalyst with a quinuclidinone reductase (*At*QR).

An aqueous solution of Tris-^{2}HCl (10 mL, 100 mM, p^{2}H 8.0) was sparged with N_2 for 60 minutes in a 25 mL round-bottom flask sealed with a Subaseal® septum. To the solution was added 3-quinuclidinone hydrochloride (48.5 mg, 30 mM) under an atmosphere of N_2. The solution stood at room temperature for 24 hours to allow for complete tautomeric exchange of the protons on C2. To the solution was added NAD^+ (3.4 mg, 0.5 mM) and it was then sparged with H_2 for a further 30 minutes. A frozen aliquot (8 mg) of carbon-supported biocatalyst was added to the solution, followed by an excess of *At*QR enzyme (500 μg, 60 U). The solution was stirred under a flow of H_2 for 20 hours, with the full conversion of the substrate to product being confirmed by GC-FID analysis. The catalyst was subsequently removed by filtration of the reaction mixture through celite. The solution was basified to pH 11–12 by the addition of K_2CO_3 (515 mg), and the solvent was removed *in vacuo* to leave a white solid. To recover the product, the solid was stirred in acetone (10 mL), which was then filtered and evaporated to dryness and the subsequent solid dissolved into C^2HCl_3 (5 × 1 mL), filtered and evaporated to dryness. (3*R*)-[2,2,3-$^2H_3$]-quinuclidinol (35 mg) was isolated as a white solid in quantitative yield. Whilst no further purification was carried out, subsequent NMR analysis revealed the presence of a small amount of glycerol, which could be avoided by more thorough washing of the enzymes prior to their use.

Elemental $C_7H_{10}{}^2H_3NO$: Found (%) C 63.1, H 11.1, N 10.3; Calc (%) C 64.6, H 12.4, N 10.8
^{1}H NMR (500 MHz, [^{2}H]-$CHCl_3$): 1.32 (m, 1H), 1.43 (m, 1H), 1.64 (m, 1H), 1.76 (*quint*, ^2J(H-H) = 3.1 Hz, 1H, H4), 1.91 (m, 1H), 2.60 (m, 1H), 2.67 – 2.72 (m, 2H)
^{13}C NMR (125.8 MHz, [^{2}H]-$CHCl_3$): 58.96 (s, CH_2), 24.85 (s, CH_2), 28.32 (s, CH), 46.31 (s, CH_2(N)), 47.36 (s, CH_2(N)), 57.09 (*quint*, ^1J(C-^{2}H) = 21.3 Hz, C^2H_2), 66.94 (*t*, ^1J(C-^{2}H) = 21.9 Hz, C^2H(OH))
^{2}H NMR (76.7 MHz, [^{2}H]-$CHCl_3$): 2.55 (s, 1^{2}H, ^{2}H2); 3.05 (s, 1^{2}H, ^{2}H2); 3.79 (s, 1^{2}H, ^{2}H3)
Enantiomeric purity (by chiral GC-FID): (3*R*)-isomer ≥ 99%, (3*S*) = not detected

Chiral GC-FID: The *ee* of the 3-quinuclidinol product was determined by chiral-phase GC-FID, by comparison of the reaction mixture with a racemic standard and a commercial enantiomerically pure standard. Samples were first basified with NaOH (10 M), extracted with a 2 × volume of EtOAc, dried over $MgSO_4$, and then centrifuged at 18,800 × g for 5 minutes before being transferred to glass vials for chiral GC-FID according to the following method:

Column: CP-Chirasil-Dex CB (Agilent), 25 m length, 0.25 mm diameter, 0.25 μm (film thickness), fitted with a guard of 10 m deactivated fused silica of the same diameter
Carrier: He (CP grade), 170 kPa (constant pressure)
Inlet temperature: 200 ˚C
Injection gases: Splitless with split flow 60 mL min^{-1}, splitless time 0.8 min, purge 5 mL min^{-1}
Injection volume: 0.1 μL
Detection: FID
Detector temperature: 250 ˚C
Detection gases: H_2 (35 mL min^{-1}), air (350 mL min^{-1}), makeup N_2 (40 mL min^{-1})
Oven heating profile:

0 → 5 mins	Hold at 70 ˚C
5 → 14.5 mins	Ramp to 165 ˚C at 10 ˚C min^{-1}
14.5 → 15.2 mins	Ramp to 200 ˚C at 50 ˚C min^{-1}
15.2 → 20.2 mins	Hold at 200 ˚C

Analysis of (3*R*)-[2,2,3-$^2H_3$]-quinuclidinol: Initially, the isolated (3*R*)-[2,2,3-$^2H_3$]-quinuclidinol was evaluated for its enantiomeric purity by chiral-phase GC-FID (see Supplementary Figure 50). The sample was compared against a racemic standard of (3*S*)- and (3*R*)-quinuclidinol (RT = 14.99 and 15.05 min, respectively) and a commercial standard of enantiomerically pure (3*R*)-quinuclidinol. Here it was established that, within the limits of detection, the *At*QR gave only the (*R*)-enantiomer. The position and extent of ^2H-labelling were then established by a combination of mass spectrometry (Supplementary Figure 51), and NMR spectroscopy (Supplementary Figure 52 and Supplementary Figure 53). The MS analysis shows the associated +3.0 mass shift from the unlabelled (m/z $[M+H]^+$ = 128.2) and tri-deuterated (m/z $[M+H]^+$ = 131.2) samples. The ^1H, ^2H, and ^{13}C NMR spectra collectively demonstrate deuteration at the targeted C3 and C2 sites. Particularly, the ^{13}C NMR spectra of the deuterated sample shows splitting of the signals at δ = 66.94 ppm and δ = 57.09 ppm, corresponding to C3 and C2, respectively. The splitting of the C3 signal into a triplet, and the C2 signal into a quintet, are consistent with coupling to one and two deuterium atoms respectively. Similarly, the ^1H NMR spectra shows a loss of signal intensity at δ = 2.54, 3.06, and 3.77 ppm upon deuteration, with appearance of peaks in these positions in the corresponding ^2H spectrum. The disappearance of the signals in the ^1H-spectrum is also accompanied by a general decrease in the *J*-coupling, and the fine structures of the other complex multiplets become better resolved. Taken together, these changes are all evidence for the high levels of deuteration reported on the C2 and C3 positions of the quinuclidinol molecule.

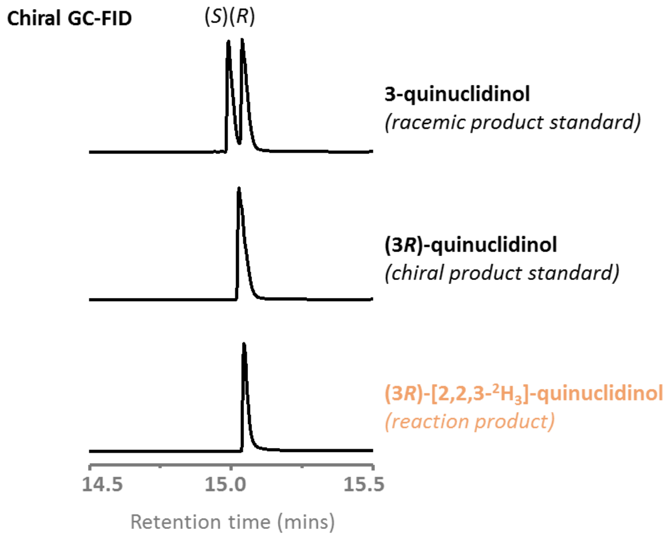

Supplementary Figure 50: Chiral GC-FID analysis of the product of the reductive deuteration of 3-quinuclidinone. Comparison of the reaction product against standards of racemic 3-quinuclidinol and (3*R*)-quinuclidinol demonstrates that only a single enantiomer is made by the biocatalytic system.

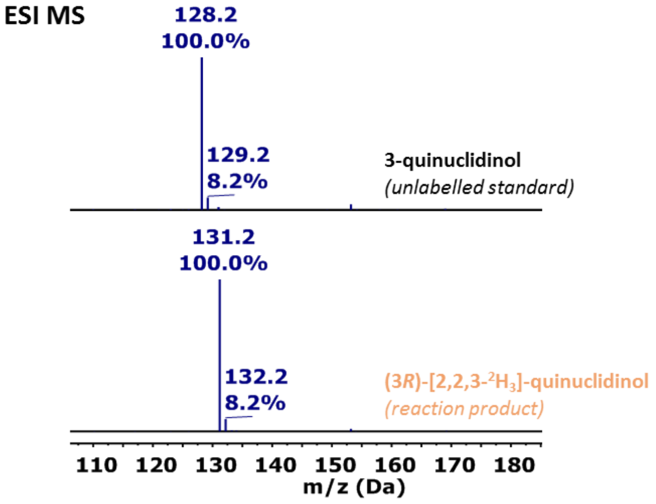

Supplementary Figure 51: Mass spectroscopic analysis of the product of the reductive deuteration of 3-quinuclidinone (ESI, positive mode). The increase in molecular weight by +3.0 of the 3-quinuclidinol product (compared to a commercial standard of isotopically unenriched 3-quinuclidinol) demonstrates the expected ^{2}H incorporation.

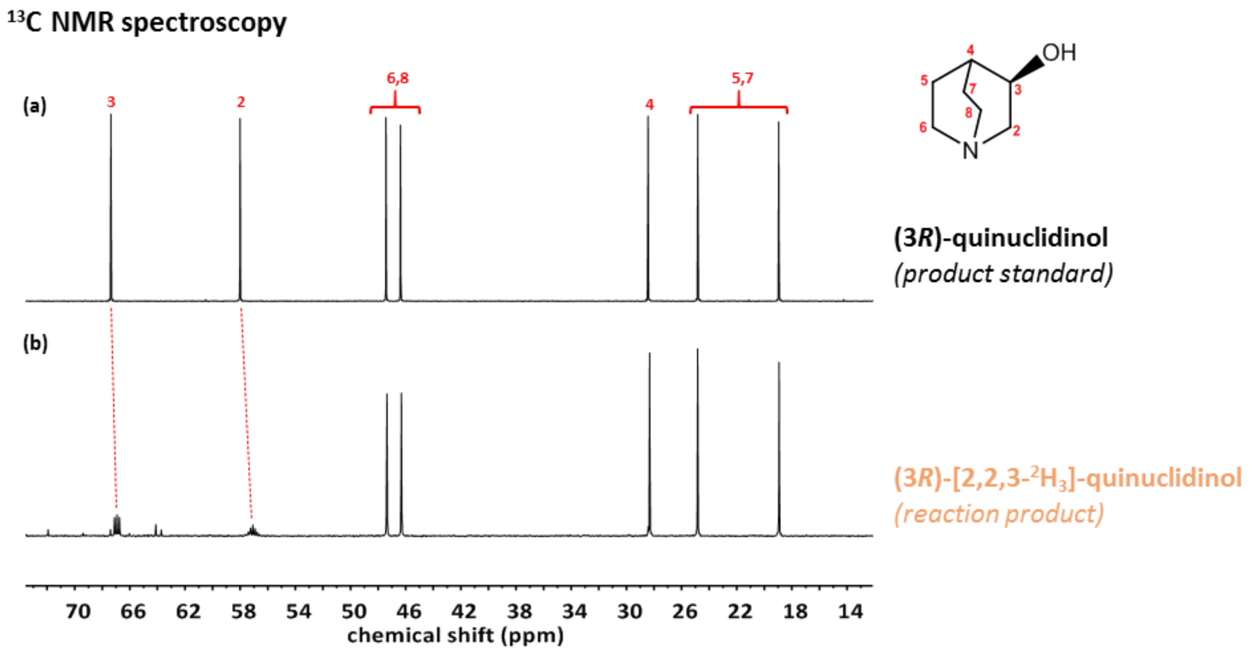

Supplementary Figure 52: ^{13}C NMR spectroscopic analysis of the product of the reductive deuteration of 3-quinuclidinone (C^2HCl_3, 125.7 MHz, 293 K). **(a)** Spectrum of isotopically unenriched standard of (3*R*)-quinuclidinol. **(b)** Spectrum of the deuterated (3*R*)-quinuclidinol product isolated from the reaction showing the ^{2}H incorporation at the C2 and C3 positions.

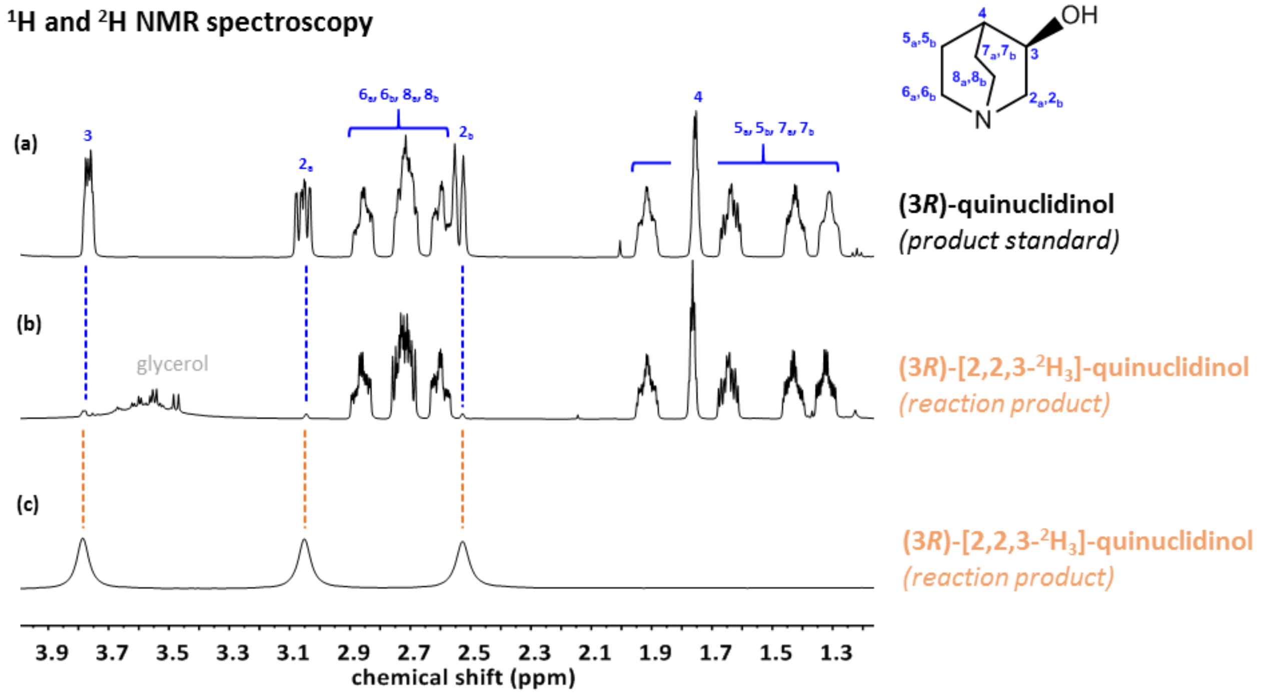

Supplementary Figure 53: ^{1}H and ^{2}H NMR spectroscopic analysis of the product of the reductive deuteration of 3-quinuclidinone (C^2HCl_3, 500 MHz, 293 K). **(a)** ^{1}H spectrum of isotopically unenriched standard of (3*R*)-quinuclidinol. **(b)** ^{1}H spectrum and (c) ^{2}H spectrum of the deuterated (3*R*)-quinuclidinol product isolated from the reaction. The absence of peaks for $H2_a$, $H2_b$, and H3 in **(b)**, and presence of these peaks in **(c)** is indicative of the targeted deuteration having been achieved.

Synthesis of (1*S*,3'*R*)-[2',2',3'-$^2H_3$]-solifenacin fumarate

(1*S*,3'*R*)-[2',2',3'-$^2H_3$]-Solifenacin fumarate was prepared according to the general scheme in Supplementary Figure 54, where (3*R*)-[2,2,3-$^2H_3$]-quinuclidinol was coupled to (1*S*)-1-phenyl-1,2,3,4-tetrahydroisoquinoline through the formation of a carbamate. Commercial (*R*)-3-quinuclidinol (Sigma Aldrich) was used to generate an isotopically unenriched sample of (1*S*,3'*R*)-solifenacin fumarate, whilst the (3*R*)-[2,2,3-$^2H_3$]-quinuclidinol (prepared in the previous section) was used to give the desired trideuterated form. In both cases, a commercial sample of (1*S*)-1-phenyl-1,2,3,4-tetrahydroisoquinoline was used to provide the second stereocentre. The procedure for both syntheses is described below:

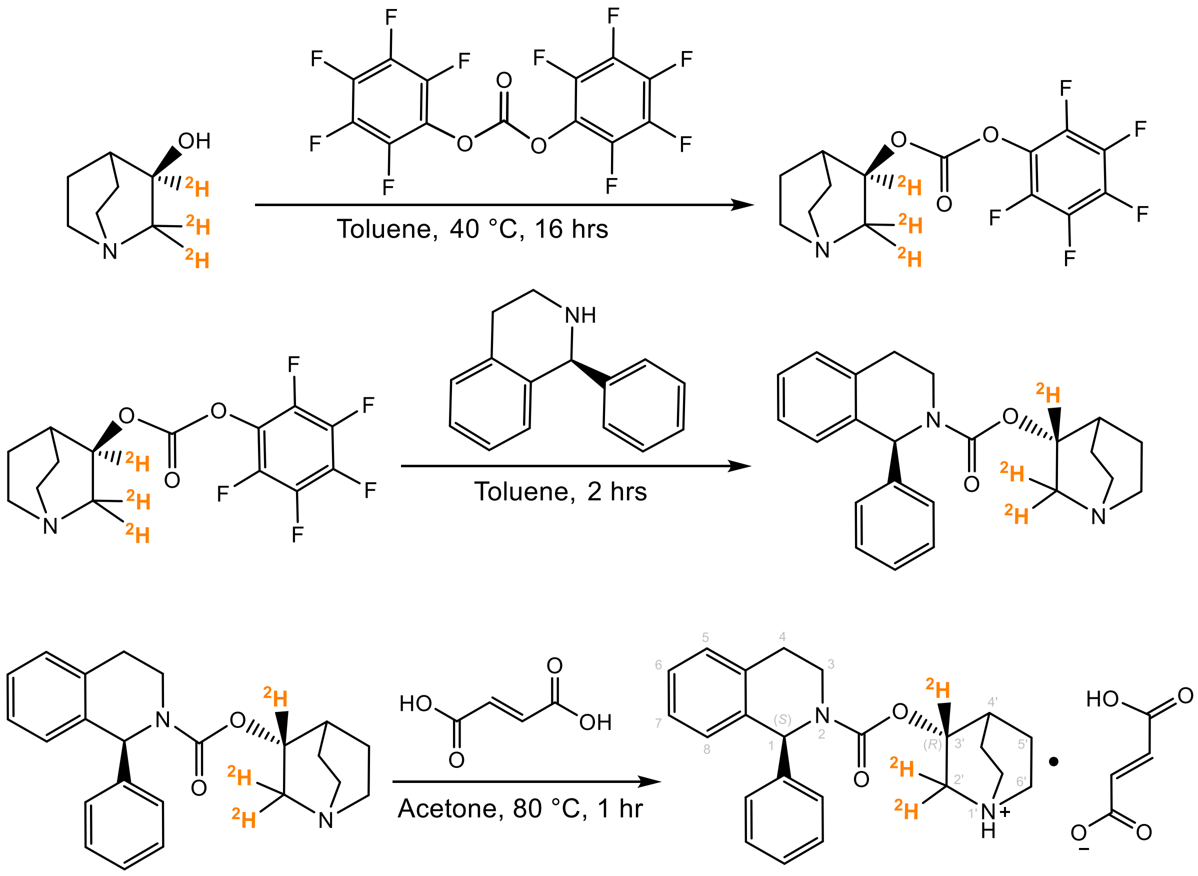

Supplementary Figure 54: Synthesis of (1*S*,3'*R*)-[2',2',3'-$^2H_3$]-solifenacin fumarate from (3*R*)-[2,2,3-$^2H_3$]-quinuclidinol. The quinuclidinol unit is coupled to (1*S*)-1-phenyl-1,2,3,4-tetrahydroisoquinoline by reaction with bis(pentafluorophenyl)carbonate, prior to the formation of the fumarate salt. The asymmetric deuterium centre created by the biocatalytic system is preserved in the final product.

(*R*)-3-Quinuclidinol or (3*R*)-[2,2,3-$^2H_3$]-quinuclidinol (30 mg, 0.23 mmol) was suspended in dry toluene (0.5 mL) containing bis(pentafluorophenyl)carbonate (102 mg, 0.26 mmol), then was stirred at 40 °C for 16 hours. After confirming the completion of the reaction by GC-FID, (1*S*)-1-phenyl-1,2,3,4-tetrahydroisoquinoline (54 mg, 0.26 mmol) was added, and the reaction stirred for a further 2 hours. The toluene was removed *in vacuo*, and the sticky yellow material was resuspended in acetone (10 mL). Fumaric acid (25 mg, 0.22 mmol) was added and the solution heated to 80 °C for 1 hour until all of the solid had dissolved. The solvent was removed *in vacuo*, and the solid resuspended in H_2O:acetone (90:10, 10 mL). The aqueous solution was washed with hexane (3 × 10 mL) and evaporated to a sticky yellow residue. The residue was suspended in toluene:acetone (70:30), evaporated to dryness, then was dissolved in MeOH and re-evaporated to provide a hygroscopic white solid. This route yielded approximately 72–75 mg of solifenacin fumarate (84–88 % yield). The ^{1}H and ^{13}C spectra could not be fully assigned unambiguously, but were consistent with those recorded elsewhere.[15,16] An epimeric mixture of (1*S*,3’*R*)- and (1*S*,3’*S*)-solifenacin fumarate was prepared in a similar manner starting from (*rac*)-3-quinuclidinol, and used as a reference for chiral-HPLC.

Data for (1*S*,3’*R*)-solifenacin fumarate

Melting point Melting upon degradation at 165 °C
HRMS $[M + H]^+$ Found 363.2065, Calc. 363.2067
Elemental $C_{27}H_{30}N_2O_6$: Found (%) C 63.0, H 6.1, N 5.2; Calc. (%) C 67.8, H 6.3, N 5.9
^{1}H NMR (500 MHz, [$^2H_4$]-MeOH): 1.72–2.22 (m, 4H); 2.38 (brs, 1H); 2.7–3.7 (m, 9H); 3.92 (brs, 1H); 5.03 (brs, 1H, H3’); 6.20 and 6.33 (2brs, 1H); 6.77 (s, 2H, $H_{fumarate}$); 7.00–7.40 (m, 9H, H_{Ar})
^{13}C NMR (125.8 MHz, [$^2H_4$]-MeOH): 18.1 (s, CH_2); 21.2 (s, CH_2); 25.4 (s, CH), 29.2 (s, CH_2); 46.7 (s, CH_2); 47.6 (s, CH_2); 54.7 (s, CH_2, C2’); 59.3 (brs, CH_2); 69.7 (s, CH, C3’); 127.3 – 130.0 (m, 10 x C_{Ar}); 136.1 (s, $CH_{fumarate}$), 156.2 (s, $COOH_{fumarate}$); 170.8 (s, $C{=}O_{carbamate}$)
Diastereotopic purity (by chiral HPLC): (1*S*,3’*R*)-isomer ≥ 99%, (1*S*,3’*S*)-isomer = not detected.

Data for (1*S*,3’*R*)-[2’,2’,3’-$^2H_3$]-solifenacin fumarate

Melting point: Melting upon degradation at 165 °C
HRMS: $[M + H]^+$ Found 366.2254, Calc. 366.2255
Elemental: $C_{27}H_{27}{}^2H_3N_2O_6$: Found (%) C 63.2, H 6.1, N 5.4; Calc. (%) C 67.3, H 5.7, N 5.8
^{1}H NMR (500 MHz, [$^2H_4$]-MeOH): 1.72–2.22 (m, 4H); 2.38 (brs, 1H); 2.7–3.7 (m, 7H); 3.92 (brs, 1H); 6.20 and 6.33 (2brs, 1H); 6.77 (s, 2H, $H_{fumarate}$); 7.00–7.40 (m, 9H, H_{Ar})
^{13}C NMR (125.8 MHz, [$^2H_4$]-MeOH): 18.1 (s, CH_2); 21.2 (s, CH_2); 25.4 (s, CH), 29.2 (s, CH_2); 46.7 (s, CH_2); 47.6 (s, CH_2); 59.3 (brs, CH_2); 127.3 – 130.0 (m, 10 x C_{Ar}); 136.1 (s, $CH_{fumarate}$), 156.2 (s, $COOH_{fumarate}$); 170.8 (s, $C{=}O_{carbamate}$).
Diastereotopic purity (by chiral HPLC): (1*S*,3’*R*)-isomer ≥ 99 %, (1*S*,3’*S*)-isomer = not detected.

Chiral HPLC-UV: The diastereotopic purity of the solifenacin product was determined by chiral-phase HPLC-UV, by comparison of the reaction mixture with a commercial standard of (1*S*,3’*R*)-solifenacin succinate, and an epimeric mixture of (1*S*,3’*S*)- and (1*S*,3’*R*)-solifenacin fumarate prepared in-house. Samples were dissolved in MilliQ H_2O and analysed on a Shimadzu UFLC LC-20AD Prominence liquid chromatograph according to the following method:

Column: Chiralpak IA column (15 cm × 4.6 mm, 5 μm particle size) with a 20 × 2.1 mm guard
Buffer: MeCN : MilliQ H_2O (90:10 vol/vol) with 20 mM NH_4OAc
Column temperature = 20 ˚C
Flow rate = 1 mL min^{-1} (isocratic)
Injection volume = 10 μL
Detection = 220 nm

Analysis of (3*R*)-[2,2,3-$^2H_3$]-quinuclidinol: Initially, the isolated (1*S*,3’*R*)-[2’,2’,3’-$^2H_3$]-solifenacin fumarate was evaluated for its diastereotopic purity by chiral-phase HPLC (Supplementary Figure 55). The sample was compared against an epimeric mixture of (1*S*,3’*S*)- and (1*S*,3’*R*)-solifenacin fumarate (RT = 11.7 and 12.6 min, respectively) and a commercial standard of (1*S*,3’*R*)-solifenacin succinate. Here it was established that, within the limits of detection, no (1*S*,3’*S*)-solifenacin was present in the final compound. The position and extent of ^2H-labelling was then established by a combination of mass spectrometry (Supplementary Figure 56), and NMR spectroscopy (see Supplementary Figure 57 and Supplementary Figure 58). The MS analysis shows the associated +3.0 mass shift from the unlabelled (m/z $[M+H]^+$ = 363.2) and tri-deuterated (m/z $[M+H]^+$ = 366.2) samples. The small residual peak at m/z = 365.2 in the (1*S*,3’*R*)-[2’,2’,3’-$^2H_3$]-solifenacin fumarate trace is consistent with a < 3% residual ^1H content across the three deuterated sites. The high signal density of the ^1H NMR spectrum in this case made identification of the deuterated sites less clear, although the disappearance of the signal at δ = 5.1 ppm (corresponding to H3’) is evidence of the high level of deuteration at the targeted α-carbon of the quinuclidinol moiety. The signals corresponding to the two H2’ protons are buried in the 2.7–3.7 ppm region, making them harder to interpret. The ^{13}C NMR spectra are much clearer however. Notably, the disappearance of the signals corresponding to C3’ (δ = 69.7 ppm) and C2’ (δ = 64.7 ppm) are consistent with the splitting of these peaks due to coupling with one and two deuterons, respectively (and subsequently being lost to the noise).

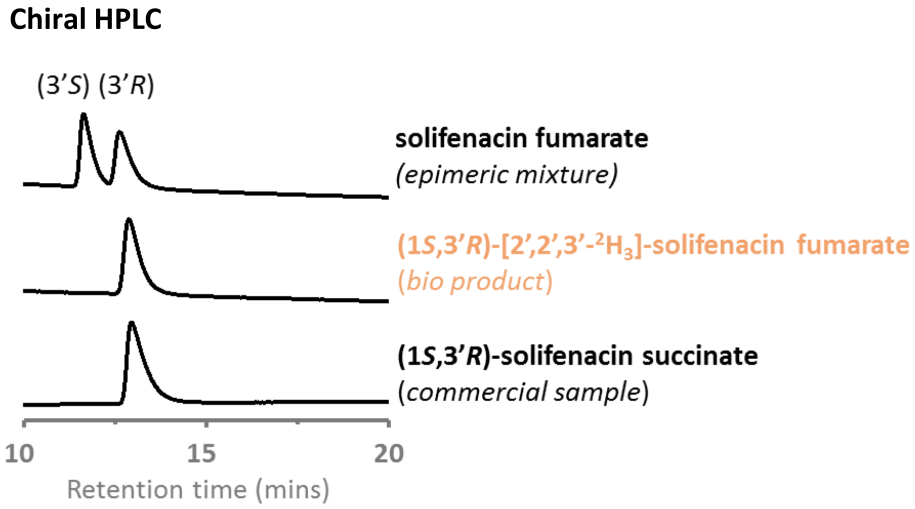

Supplementary Figure 55: Characterisation of deuterated solifenacin fumarate by chiral HPLC. Comparison of the product synthesised using the biocatalytic system against standards of epimeric solifenacin fumarate (produced in-house) and a commercial sample of (1*S*,3’*R*)-solifenacin succinate demonstrates that only the desired (3’*R*)-epimer was formed.

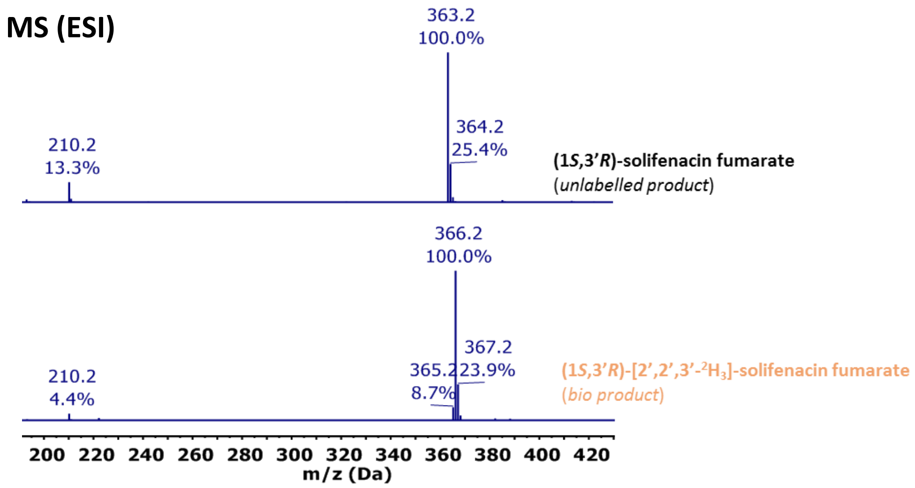

Supplementary Figure 56: Mass spectroscopic analysis of deuterated solifenacin fumarate (ESI, positive mode). Comparison of the (1*S*,3'*R*)-[2',2',3'-$^{2}H_{3}$]-solifenacin fumarate product to an isotopically unenriched standard (produced in-house) demonstrates the expected ^{2}H incorporation.

^{13}C NMR spectroscopy

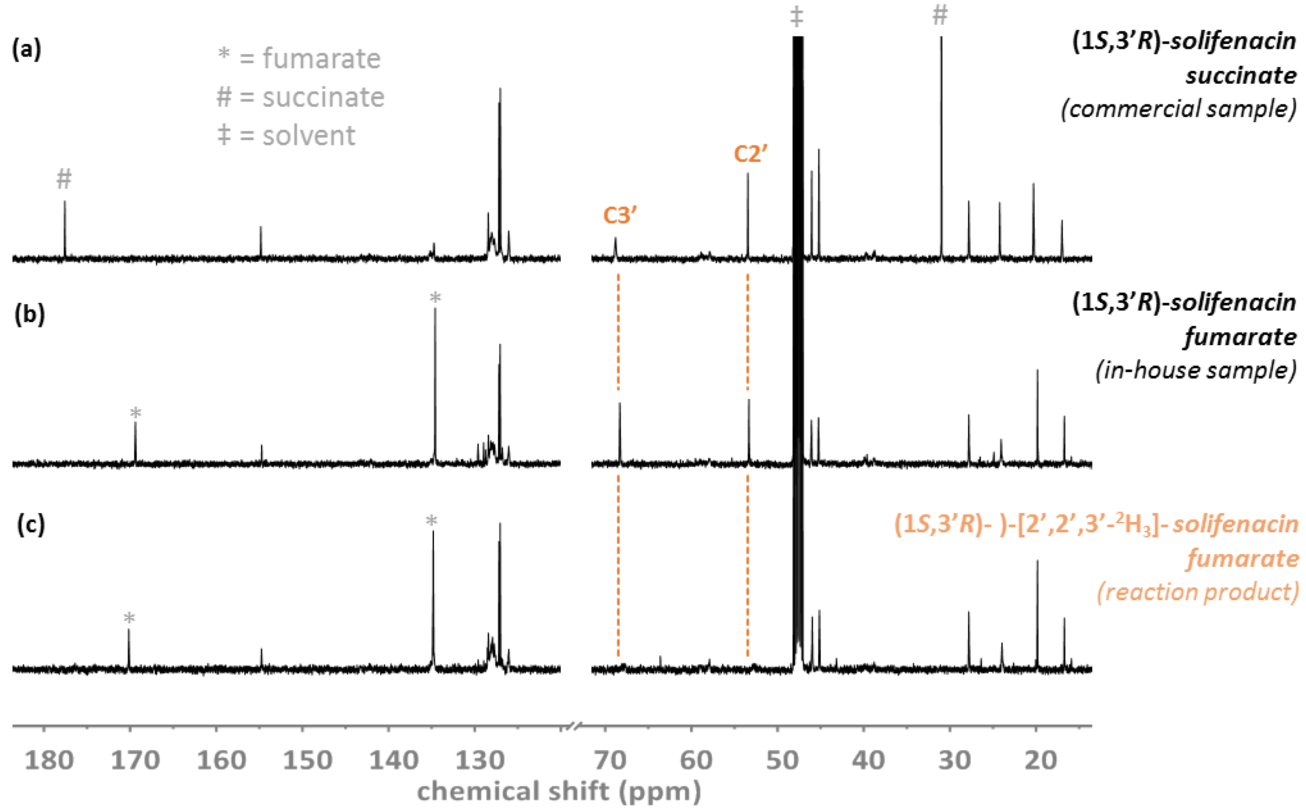

Supplementary Figure 57: ^{13}C NMR spectroscopic analysis of deuterated solifenacin fumarate ([$^{2}H_{4}$]-CH_3OH, 125.7 MHz, 293 K). **(a)** Spectrum of isotopically unenriched standard of (1*S*,3'*R*)-solifenacin succinate (commercial). **(b)** Spectrum of (1*S*,3'*R*)-solifenacin fumarate (prepared in house). **(c)** Spectrum of (1*S*,3'*R*)-[2',2',3'-$^{2}H_{3}$]-solifenacin fumarate prepared using the biocatalytic system. Comparing **(c)** with **(a)** and **(b)** demonstrates the ^{2}H incorporation at the C2 and C3 positions

^{1}H NMR spectroscopy

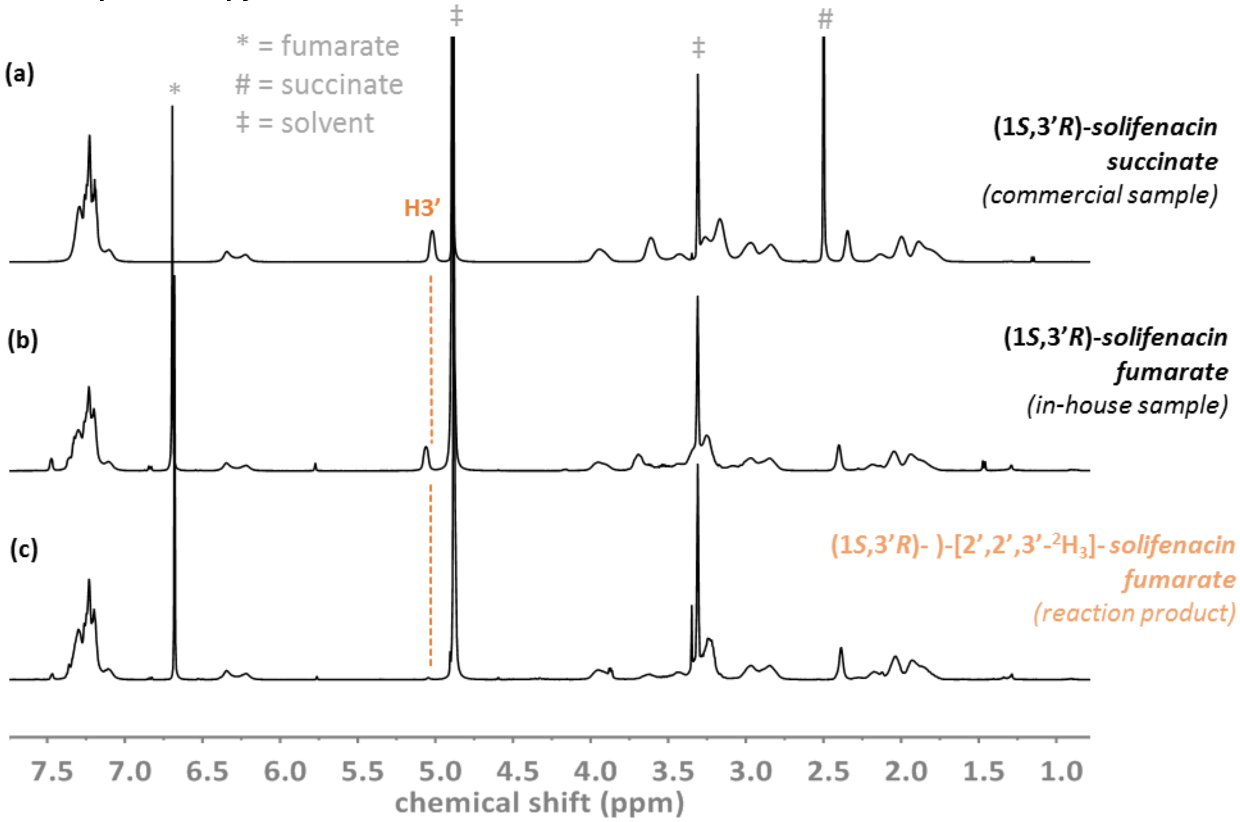

Supplementary Figure 58: ^{1}H NMR spectroscopic analysis of deuterated solifenacin fumarate ([$^{2}H_4$]-CH_3OH, 500 MHz, 293 K). **(a)** Spectrum of isotopically unenriched standard of (1*S*,3'*R*)-solifenacin succinate (commercial). **(b)** Spectrum of (1*S*,3'*R*)-solifenacin fumarate (prepared in house). **(c)** Spectrum of (1*S*,3'*R*)-[2',2',3'-$^{2}H_3$]-solifenacin fumarate prepared using the biocatalytic system. The absence of the signal of H3' in spectrum **(c)** is indicative of the deuteration of C3'.

Supplementary References

1. Reeve, H. A. *et al.* Enzymes as modular catalysts for redox half reactions in H_2-powered chemical synthesis: From biology to technology. *Biochem. J.* **474,** 215–230 (2017).
2. Lauterbach, L., Idris, Z., Vincent, K. A. & Lenz, O. Catalytic Properties of the Isolated Diaphorase Fragment of the NAD^+-Reducing [NiFe]-Hydrogenase from Ralstonia eutropha. *PLoS One* **6,** e25939 (2011).
3. Birrell, J. A. & Hirst, J. Investigation of NADH Binding, Hydride Transfer, and NAD^+ Dissociation during NADH Oxidation by Mitochondrial Complex I Using Modified Nicotinamide Nucleotides. *Biochemistry* **52,** 4048–4055 (2013).
4. Mostad, S. B. & Glasfeld, A. Using High Field NMR to Determine Dehydrogenase Stereospecificity with Respect to NADH. *J. Chem. Educ.* **70**, 504–506 (1993).
5. Hou, F. *et al.* Expression, purification, crystallization and X-ray analysis of 3-quinuclidinone reductase from *Agrobacterium tumefaciens*. *Acta Crystallogr. Sect. F Struct. Biol. Cryst. Commun.* **68,** 1237–1239 (2012).
6. Bertrand, M., Chabin, A., Brack, A. & Westall, F. Separation of amino acid enantiomers VIA chiral derivatization and non-chiral gas chromatography. *J. Chromatogr. A* **1180,** 131–137 (2008).
7. Leiva de Faria, M. *et al.* Enantiodivergent syntheses of cycloheptenone intermediates for guaiane sesquiterpenes. *Tetrahedron: Asymmetry* **11,** 4093–4103 (2000).
8. Shimoda, K. & Hirata, T. Biotransformation of enones with biocatalysts — two enone reductases from *Astasia longa*. *J. Mol. Catal. B Enzym.* **8,** 255–264 (2000).
9. Knaus, T., Toogood, H. S. & Scrutton, N. S. Ene-reductases and their Applications in *Green Biocatalysis*, 473–488 (John Wiley & Sons, Inc, 2016).
10. Durndell, L. J. *et al.* Selectivity control in Pt-catalyzed cinnamaldehyde hydrogenation. *Sci. Rep.* **5,** 9425 (2015).
11. Venema, A., Nibbering, N. M. M. & De Boer, T. J. Mass spectrometry of aralkyl compounds with a functional group—IX: Hydrogen scrambling in the molecular ion of hydrocinnamaldehyde. *Org. Mass Spectrom.* **3,** 583–595 (1970).
12. Könst, P. *et al.* Enantioselective Oxidation of Aldehydes Catalyzed by Alcohol Dehydrogenase. *Angew. Chemie Int. Ed.* **51,** 9914–9917 (2012).
13. Wuensch, C. *et al.* Asymmetric Biocatalytic Cannizzaro-Type Reaction. *ChemCatChem* **5,** 1744–1748 (2013).
14. Nibbering, N. M. M. & de Boer, T. J. Mass spectrometry of aralkyl compounds with a functional group—III: Specific exchange between some hydrogen atoms in the molecular ion of γ-phenylpropanol. *Tetrahedron* **24,** 1415–1426 (1968).
15. Trinadhachari, G. N. *et al.* An improved process for the preparation of highly pure solifenacin succinate via resolution through diastereomeric crystallisation. *Org. Process Res. Dev.* **18,** 934–940 (2014).
16. Fuentes, G. G., Bonde-Larsen, A. L., Jaime del Campo López-Bachiller, Celso Sandoval Rodríguez & Sainz, Y. F. Solifenacin salts. US Patent 8,765,785 B2 (2014).
